# Functional phases define the response of the soil microbiome to environmental change

**DOI:** 10.1101/2024.03.15.584851

**Published:** 2024-03-28

**Authors:** Kiseok Keith Lee, Siqi Liu, Kyle Crocker, David R. Huggins, Mikhail Tikhonov, Madhav Mani, Seppe Kuehn

**Affiliations:** 1 Department of Ecology and Evolution, The University of Chicago, Chicago, IL 60637, USA; 2 Center for the Physics of Evolving Systems, The University of Chicago, Chicago, IL 60637, USA; 3 Department of Engineering Sciences and Applied Mathematics, Northwestern University, Evanston, IL 60208, USA; 4 NSF-Simons Center for Quantitative Biology, Northwestern University, Evanston, IL 60208, USA; 5 USDA-ARS, Northwest Sustainable Agroecosystems Research Unit, Pullman, WA 99164, USA; 6 Department of Physics, Washington University in St. Louis, St. Louis, MO 63130, USA; 7 National Institute for Theory and Mathematics in Biology, Northwestern University and The University of Chicago, Chicago, IL; 8 Center for Living Systems, The University of Chicago, Chicago, IL 60637, USA

## Abstract

A major challenge in microbiome research is understanding how natural communities respond to environmental change. The ecological, spatial, and chemical complexity of soils makes understanding the metabolic response of these communities to perturbations particularly challenging. Here we measure the dynamics of respiratory nitrate utilization in >1,500 soil microcosms from 20 soil samples subjected to pH perturbations. Despite the complexity of the soil microbiome a minimal mathematical model with two parameters, the quantity of active biomass and the availability of a limiting nutrient, quantifies observed nitrate utilization dynamics across soils and pH perturbations. Across environmental perturbations, the model reveals the existence of three functional phases each with distinct qualitative dynamics of nitrate utilization over time: a phase where acidic perturbations induce cell death that limits metabolic activity, a nutrient-limiting phase where nitrate uptake is performed by dominant taxa that utilize nutrients released from the soil matrix, and a resurgent growth phase in basic conditions, where nutrients are in excess and rare taxa rapidly outgrow dominant populations. The underlying mechanism of each phase is predicted by our interpretable model and tested via amendment experiments, nutrient measurements, and sequencing. Finally, our data suggest that how soils transition between functional phases depends on the long-term history of environmental variation in the wild. Therefore, quantitative measurements and a minimal mathematical formalism reveal the existence of qualitative phases that capture the mechanisms and dynamics of a community responding to environmental change.

## Introduction

Soil, marine, and freshwater microbiomes perform carbon and nitrogen transformations that sustain biogeochemical cycles and thereby life in the biosphere [1–3]. Natural microbiomes are exposed to environmental perturbations including changes in temperature, pH, moisture, oxygen, and nutrients stemming from either natural or anthropogenic events. Therefore, characterizing how these communities respond to natural and anthropogenic environmental perturbations is of critical importance. Understanding how these responses are shaped by community composition [4] may translate into principles for controlling community metabolic processes or predicting future responses to environmental change. However, due to the complexity of natural communities, establishing a quantitative link between microbiome metabolism, community composition, and environmental perturbations has proven challenging [5, 6].

This complexity is perhaps the most apparent in soils, which harbor immense taxonomic diversity [7], extensive spatial structure [8], and chemically diverse environments [9]. As a result, environmental perturbations can modify collective metabolic activity via a myriad of mechanisms, from indirect modification of nutrient availability [10,11] to changes in microbial physiology [12], ecological interactions [13, 14], or spatial organization [8, 15]. Identifying which of these processes are the key drivers of the community-level metabolic response remains a challenge.

Surveys of communities in the wild [16–18] reveal patterns relating community composition, environmental variation, and metabolic processes [19–28]. However, the origins of these patterns are difficult to dissect [14], and establishing causal relationships from observational data alone is challenging given many confounding factors. Accordingly, significant effort has been devoted to lab-based experiments that modulate microbiome structure and environmental conditions while making precise functional measurements [29–32]. These studies can reveal causal principles of community assembly [32–34], however, the results are hard to generalize to natural communities due to lower community diversity and experimental culture conditions that depart strongly from nature.

To resolve these limitations while retaining the advantages of both laboratory experiments and natural surveys, we conducted ~ 1500 parallelized soil microcosm experiments, where soil samples from the environment are incubated, perturbed, and assayed in the lab [35–38]. This approach retains much of the chemical, ecological, and spatial complexity of natural systems while enabling experiments at scale that control for confounding factors to identify causal relationships. We find that quantifying metabolite and abundance dynamics in soil microcosms coupled with judicious mathematical modeling can reveal principles governing how environmental variation impacts soil microbiome metabolism.

Despite the complexity of soils, we show that the diversity of nitrate utilization dynamics in soils subjected to pH perturbations can be quantitatively distilled to a conserved set of functional phases. Functional phases are qualitative regimes that capture the time dynamics of nitrate utilization by the entire microbiome. Functional phases are characterized by the quantity of metabolically active biomass and the availability of limiting nutrients, parameters that emerge naturally from a mathematical description of community-level nitrate utilization dynamics. The mathematical description provides a route to understanding the complex abundance dynamics across thousands of distinct taxa while revealing mechanisms underlying the three phases: An acidic phase (I) is characterized by severe nutrient limitation and death. A nutrient-limiting phase (II), is characterized by a large active population with nutrients-limiting nitrate utilization rates. Phase III, in alkaline conditions, is characterized by the inability of abundant populations to perform nitrate utilization giving rise to the rapid growth of a member of the rare biosphere. The existence of phases reveals that the metabolic response of the soil to environmental change is governed by only a few key properties of the microbiome. The transition between phases II and III is abrupt and occurs when a fixed quantity of base is added to the system. The transition between Phase I and II is smooth and depends on the native pH of the soil. Thus, while the dynamics and mechanisms of each functional phase are conserved across soils, the transitions between phases depend on environmental history and community composition. Our study establishes the notion that a few simple and interpretable functional phases encode how complex microbiomes respond to environmental change.

## Results

We focus on the utilization of nitrate $\left({NO}_{3}^{-}\right)$ which is used as an electron acceptor during anaerobic respiration. We study nitrate utilization in soils subjected to pH perturbations because pH is a key environmental parameter [39–41] and nitrate utilization is critical for agriculture and climate. Decades of studies have examined how pH affects nitrate utilization [42]. However, discrepancies in experimental methods (Table S1) and limited quantitative modeling, have made it difficult to find principles governing metabolic responses to pH perturbations [20], resulting in debates over the optimal pH for nitrate utilization (Table S2). Our discovery of phases provides a unifying picture that resolves these inconsistencies.

### Metabolite dynamics in soils after short and long-term pH perturbations

**Figure 1:**
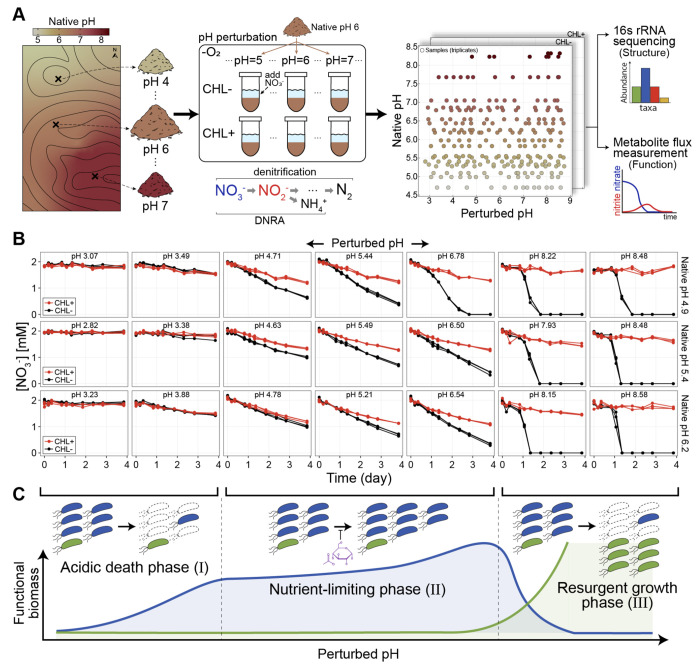
Soil microbiome metabolite and abundance dynamics under long and short-term pH variation. (**A**) Schematic of the field sampling for soils with long-term pH variations (20 soils, pH4.7 to 8.3, Cook Agronomy Farm, Pullman, WA, USA) and the experimental setup for imposing short-term pH perturbations in laboratory conditions. With each of the 20 soils, we created slurries (1:2 soil:water) amended with 2mM nitrate, adjusted to 13 different pH levels, and treated with (CHL+, no growth) or without chloramphenicol (CHL−, growth). ∼1,500 microcosms are depicted in a grid of different pH conditions (perturbed pHvs. native pH) each condition in triplicates. Microcosms were incubated anaerobically for a 4-days while nitrate and nitrite were quantified colorimetrically. For metabolic dynamics, we measured nitrate $\left({NO}_{3}^{-}\right)$ and nitrite $\left({NO}_{2}^{-}\right)$ flux, the first two intermediates in denitrification and dissimilatory nitrate reduction to ammonium (DNRA). Communities were quantified by 16 S rRNA amplicon sequencing before and after slurry incubation. (**B**) A subset of nitrate concentration dynamics (function) during the 4-day anaerobic incubation: three topsoils with different native pH levels (rows) were perturbed to either acidic or basic pH (columns) at the start of the incubation ($T_{0}$), all with (CHL+, red) and without chloramphenicol (CHL−, black) treatments in triplicates (see Methods). (**C**) Schematic depicting three different functional phases that capture how the soil community responds to pH perturbations. With moderate pH perturbations, the functional response can be characterized as the Nutrient-limiting phase (Phase II), where nitrate utilization is performed by dominant taxa (blue) that utilize nutrients released from the soil matrix due to perturbation. Growth is limited by the amount of available growth-limiting nutrients (purple). During strong basic perturbations, growth-limiting nutrients are in excess, and rare taxa (green) rapidly outgrow dominant populations that cannot perform nitrate reduction in basic conditions, hence the Resurgent growth phase (Phase III). Strong acidic perturbations induce cell death that limits metabolic activity, resulting in an inactive state (Acidic death phase, Phase I). Functional biomass of the dominant (blue) and rare (green) taxa are shown by the lines below.

To address this problem we measured nitrate utilization dynamics in soil microcosms across a range of native and perturbed pH levels. We sampled 20 top soils with pH from 4.7 to 8.3 (Fig. 1A, Table S3) at the Long-term Agricultural Research Cook Agronomy Farm (CAF) (Pullman, WA, USA). Sampling from a single area helped minimize the effect of confounding factors, and we obtained soils with similar characteristics (Table S3). The long-term variation of soil pH in the site arises from local agricultural practices and erosion.

For each soil sample, we created mixtures of soil and water (slurries) with 2mM nitrate and used varying levels of strong acid or base to perturb each soil’s native pH to 13 values between 3 and 9 (Fig. 1A). In this way, our experiment directly measures the effects of short-term pH perturbations, while the differences between soils can inform us about the effects of long-term exposure to low/high pH. We employed slurries to make amendments easier, limit the effect of differential water content, and mimic rain events when most of the anaerobic respiratory nitrate utilization occurs [43, 44].

Soil slurries retained much, but not all, of the complexity in the natural context, including the diversity of the communities, the soil nutrient composition, and the spatial structure due to intact soil grains. The metabolic activity we observed relied only on the natively available carbon (electron donor for nitrate reduction), and thus we did not enrich for specific taxa beyond the nitrate added to the system. To separate the activity of pre-existing nitrate utilizers from growth in each condition [45], we included controls in every pH perturbation treated with chloramphenicol which inhibits protein synthesis (Fig. 1A). The dataset comprised 20 soils, at 13 distinct pH levels, with and without chloramphenicol, each in triplicate.

For each of these treatments, we measured the dynamics of the relevant metabolites (nitrate, nitrite, ammonium, and water-soluble organic carbon) during the 4-day incubation in anaerobic conditions (Fig. 1A). Focusing on non-gaseous metabolites enabled us to perform high temporal resolution measurements of metabolite dynamics across the ∼1500 microcosms. Additionally, for 10 of 20 soils, we performed 16S rRNA amplicon sequencing before and after incubation.

We observed three types of dynamics that were conserved across pH perturbations and soils (Fig. 1B). First, all chloramphenicol-treated (CHL+) conditions exhibited linear nitrate $\left({NO}_{3}^{-}\right)$ utilization dynamics (red lines, Fig. 1B, Figs. S1, S2). This is expected because, with chloramphenicol, nitrate reducers are unable to grow, and the rate of nitrate reduction remains constant [43]. The slope of the CHL+ nitrate dynamics quantifies the activity of the pre-existing functional biomass. Second, we observed linear nitrate/nitrite reduction dynamics even in samples without chloramphenicol (CHL−) for pH perturbations around the native pH (black lines, Fig. 1B, Figs. S2, S3). This indicates that, after some early growth, the functional biomass stays constant even without the growth-inhibiting drug (CHL−). This suggests that the growth of the functional biomass is likely limited by nutrients other than nitrate (schematic, Fig. 1C). Third, when we perturb the pH above 8, we observe an initial lag of nitrate reduction, followed by an exponential increase in reduction rate (black lines, far right, Fig. 1B). This indicates that an initially rare population grows rapidly to reduce all available nitrate, whereas the pre-existing dominant population fails to perform nitrate reduction at this pH (flat CHL+ nitrate dynamics, far right; Fig. 1B).

### Simple consumer-resource model captures metabolite dynamics across all pH perturbations

**Figure 2:**
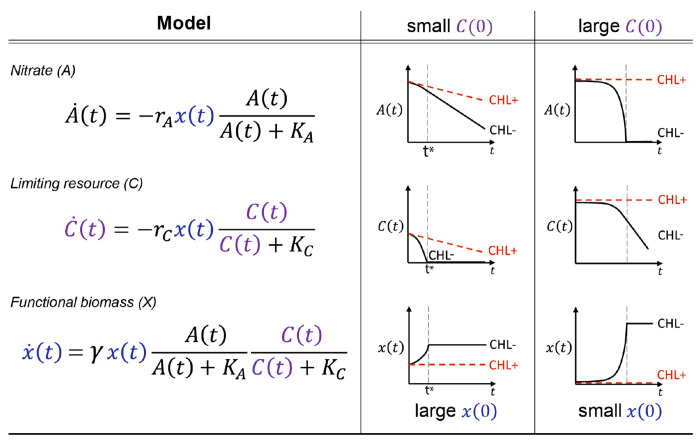
Consumer-resource model captures metabolite dynamics A mathematical representation of the consumer-resource model to fit the nitrate reduction dynamics of the community (**Model** column). The model describes the community through the total functional biomass (x, biomass) which describes the aggregated biomass of species that perform nitrate reduction, nitrate concentration (A,mM), and a limiting resource concentration (C,mM). Nitrate consumption rate $\left(\dot{A}(t)\right)$ takes a Monod [46] form with a reduction rate parameter $\left(r_{A},mM/\right.$ biomass/day) and an affinity parameter $\left(K_{A},mM\right)$. Nitrite, which is reduced from nitrate, is not modeled. To capture linear nitrate dynamics, we include a non-substitutable resource that limits growth (C) with Monod consumption function and parameters $r_{C}(mM/$ biomass/day) and affinity parameter $K_{C}(mM)$. Growth of functional biomass $\left(\dot{x}(t)\right)$ is determined by concentrations of nitrate (A) and limiting nutrient (C) with biomass growth rate (γ,1/ day). Plots in the right two columns show dynamics of x(t),A(t), and C(t) at small C(0) and large x(0) (middle) and large C(0) with small x(0) (right). Red and black traces show dynamics with and without growth-inhibiting $(\dot{x}(t)=0$) chloramphenicol respectively. Without growth, the nitrate reduction rate is constant and proportional to the functional biomass x(0) (red lines, top row for large/small (0)). The small C(0) column illustrates how the model captures linear nitrate dynamics in chloramphenicol untreated (CHL−) conditions (black lines). With the small amount of initial limiting resource C(0), functional biomass will stay constant after the limiting nutrient is depleted at $t^{*}$, which produces a constant nitrate reduction rate (linear ${NO}_{3}^{-}$ dynamics, black line, top). The large C(0) column shows exponential nitrate depletion dynamics in CHL− conditions (black lines) when there is excess C(0) and x(0) is small. Functional biomass grows exponentially, resulting in exponential nitrate utilization dynamics (black line, top). The affinity parameters $\left(K_{A},K_{C}\right)$ and yield parameter (γ) were fixed for all samples (see Methods for rationale and Fig. S8).

To capture these observations, we used the consumer-resource model presented in Fig. 2. Crucially, this model subsumes the ecological complexity of the soil microbiome into a single effective biomass rather than explicitly considering the multitude of possible interactions between taxa. The model has 3 variables: the functional nitrate-utilizing biomass (x), nitrate concentration (A), and the second growth-limiting nutrient (C) whose existence we hypothesized based on the observations described above. The five model parameters include: consumption rates $\left(r_{A}\right.$ and $r_{C}$), growth rate (γ), and affinities $\left(K_{A}\right.$ and $\left.K_{C}\right)$. The consumption rate of a resource is determined by the amount of functional biomass (x) and per-biomass consumption rates. The biomass growth rate $\left(\dot{x}\right)$ is set to zero in CHL+ conditions due to chloramphenicol inhibition $\left(\gamma =0,\dot{x}=0\right)$.

If the initial nutrient concentration C(0) is small (Fig. 2, middle column), the nutrient C runs out quickly, arresting biomass growth and resulting in A being consumed at a constant rate from $t^{*}$ onwards (dashed line). This recapitulates the late-time linear dynamics in CHL− conditions (Fig. 1B). In contrast, when the initial nutrient concentration C(0) is large (Fig. 2, right column), it is nitrate (A) that runs out first. In this regime, the initial rate of nitrate utilization (determined by x(0), the initial functional biomass) grows exponentially until A runs out. Therefore, a small x(0) and a large C(0) recapitulates the initially slow but exponentially growing dynamics observed at greater pH perturbations (Fig. 1B).

Our consumer-resource model provided a good fit to the observed nitrate dynamics in all soils (<10% error per data point, Fig. S7). To perform this fitting, we fixed the growth rate γ and the affinity parameters $\left(K_{A},K_{C}\right)$, and varied just two rescaled parameters: $\tilde{x}(0)=x(0)r_{A}$ and $\gamma \tilde{C}(0)=\gamma C(0)r_{A}/r_{C}$ (see Methods). These parameters retain the interpretation above $\left(\tilde{x}(0\right)$ reflects the initial functional biomass, and $\gamma \tilde{C}(0)$ the available limiting nutrient); the rescaling merely corresponds to measuring both these quantities in terms of equivalent nitrate utilization rate (see Methods). Next, we examine how these two key inferred quantities vary under pH perturbation, and across soils with different native pH.

### Model reveals functional phases that capture metabolite dynamics across pH perturbations

**Figure 3:**
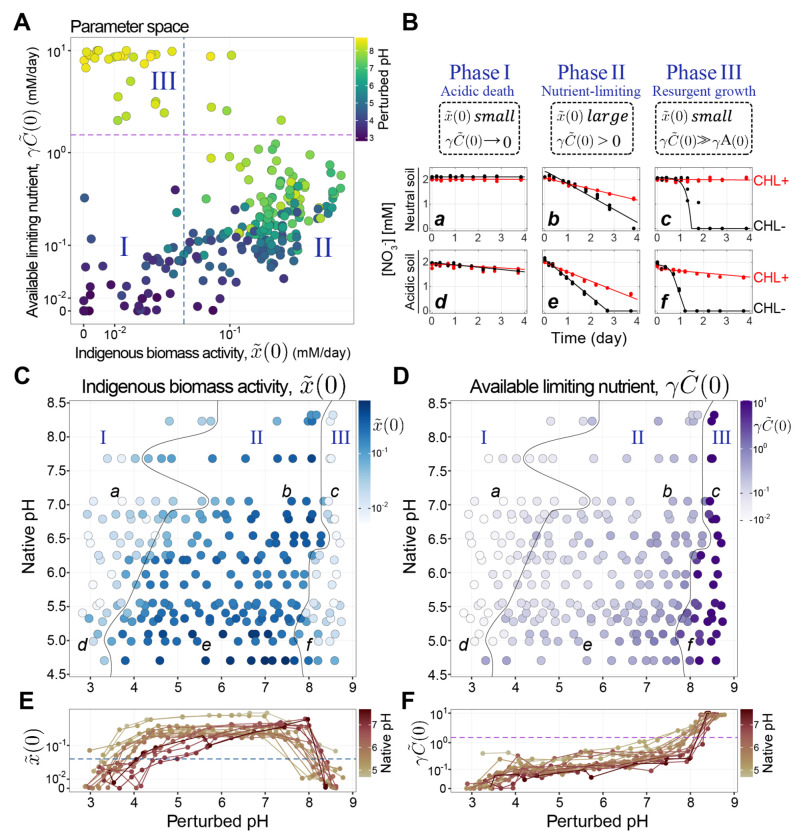
Conserved phases capture soil’s functional response to pH perturbations. **(A)** Scatterplot of the two model parameters (functional biomass $\tilde{x}(0)$, limiting nutrient concentration $\gamma \tilde{C}(0)$ ) inferred from nitrate dynamics across all samples. See text and Methods for details of model fitting. Note log-scale. The color of points indicates each sample’s perturbed pH. Three regions separated by dashed lines indicate the distinct phases of functional response against pH perturbations: the Acidic death phase (Phase I), Nutrient-limiting phase (Phase II), and Resurgent growth phase (Phase III). The locations of the dashed lines were determined by thresholding distributions of $\tilde{x}(0)$ and $\gamma \tilde{C}(0)$ (see Methods). (B) Example nitrate dynamics for each of the three phases for a neutral soil (top row) and an acidic soil (bottom row). Red lines are with growth-arresting chloramphenicol and black without. (,b,c,d,e,f correspond to perturbed conditions indicated in panels C **and D**). a,d show little nitrate reduction, b,e show linear nitrate dynamics with slopes that increase without chloramphenicol (see Fig. 2, middle column), and c,f show no activity without growth (red) but exponential nitrate utilization in the absence of the drug (Fig. 2, right column). **(C) and (D)** pH affects indigenous biomass activity $\tilde{x}(0)$ (blue) and available limiting nutrient $\gamma \tilde{C}(0)$ (purple). Fitted parameter values are shown with the color (log-scale) in the grid of long-term pH variation (y-axis, Native pH) and short-term pH perturbation (x-axis, Perturbed pH). In all soils from different native pH levels, we observe a conserved set of responses to short-term pH perturbations: Nutrient-limiting phase (region indicated by II) near the native pH, then transitioning to the Acidic death phase (region indicated by I) during acidic perturbation (black line), also transitioning to the Resurgent growth phase (III) for basic perturbations (black line). Long-term (native) pH dictates the pH thresholds of phase boundaries (black line). **(E)** Trends of $\tilde{x}(0)$ (log-scale) across varying perturbed pH values for soils with different native pH levels (native pH indicated by line color), demonstrating consistent phase transitions and a plateau of high activity within the mid-range pH (Phase II) across all soils. **(F)** $\gamma \tilde{C}(0)$ (log-scale) with perturbed pH, showing a rise in limiting nutrients induced by short-term pH increases. Colors indicate native pH. We used the median fitted value of the three biological replicates for all data points of $\tilde{x}(0)$ and $\gamma \tilde{C}(0)$.

We plotted $\tilde{x}(0)$ (indigenous functional biomass) against $\gamma \tilde{C}(0)$ (available limiting nutrient, Fig. 3A) and identified three phases of nitrate utilization dynamics (Methods, Fig. S9). Phase I, the Acidic death phase (both $\tilde{x}(0)$ and $\gamma \tilde{C}(0)$ are low) is observed for pH≲4, and shows little to no nitrate reduction with or without chloramphenicol (Fig. 3B, (a) and (d)). Phase II, the Nutrient-limiting phase (both $\tilde{x}(0)$ and $\gamma \tilde{C}(0)$ are large) is observed for 4≲pH≲8, and exhibits a relatively large initial nitrate reduction rate followed by a transient speed-up to a new constant rate (Fig. 3B, (b) and (e)). Finally, Phase III, the Resurgent growth phase (small $\tilde{x}(0)$, large $\gamma \tilde{C}(0))$ is observed for pH≳8, and displays a close-to-zero initial reduction rate, followed by an exponential speed-up that continues until nitrate is depleted (Fig. 3B, (c) and (f)).

We observe all three functional phases across all soils, but the pH at which a transition from one phase to another occurs depends on the native pH of the soil. Figure 3C & D shows the inferred initial functional biomass $\left(\tilde{x}(0)\right)$ and limiting nutrient $\left(\gamma \tilde{C}(0)\right)$ across soils of varying native pH (y-axis) and laboratory perturbed pH (x-axis). More basic soils transition from Phase II to Phase I at a higher pH than acidic soils. We next harnessed our model to identify mechanisms underlying these phases and test them via sequencing and experiments.

### Metabolite dynamics in Phase II are governed by carbon release

In Phase II (the Nutrient-limiting phase), soils exhibit high native functional biomass and a nitrate reduction rate increasing with pH (Fig. 1B). Our model suggests that this increase derives from the increasing availability of the limiting nutrient (Fig. 3F) translating into larger growth of active biomass (Fig. 2). Here we test this prediction of the model.

**Figure 4:**
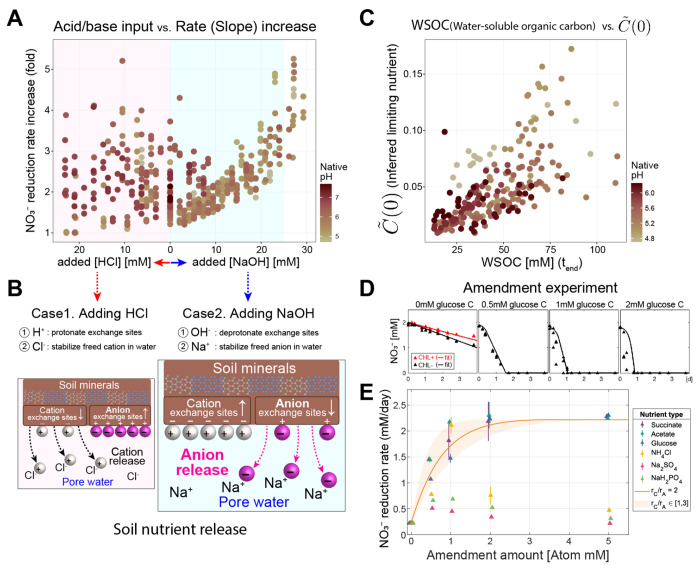
Carbon limits growth in phase II and is released by ion exchange mechanism **(A)** The amount of NaOH added to the soil plotted against the fold-change of nitrate reduction rate (ratio of rate with growth (CHL−) and with no growth (CHL+)). Base additions from 0mM to 25mMNaOH correspond to soils in the Nutrient-limiting phase (Phase II, Fig. 3) (light blue background in **A**). No increase in growth was observed for acidic perturbations (>0mMHCl addition, pink region, Fig. S11A). **(B)** Cartoon illustrating the mechanism of soil nutrient release hypothesis; NaOH results in the release of anionic nutrients (magenta-colored spheres) from soil particles (brown region), while the addition of HCl would release cationic nutrients (white spheres) and adsorbs anionic nutrients. Microbes cannot access the nutrients adsorbed in the soil particles but can access the nutrients dissolved in pore water. Added ${OH}^{-}$ ions decrease the number of anion exchange sites in the soil particles, releasing anionic nutrients. In concert, ${Na}^{+}$ ions stabilize the released anions (see text and Fig. S13B for additional details). **A** and **B** suggest the growth-limiting nutrients are anionic (negatively charged). **(C)** Scatterplot of model-inferred $\tilde{C}(0)$ (available limiting nutrient) and measured water-soluble organic carbon (WSOC) measured via a chromate oxidation assay (Methods) that is not chemically specific and WSOC likely contains different C compounds, N, P, etc. **(D) and (E)** Amendment experiments for soil in the Nutrient limiting phase (Phase II) at unperturbed pH. **(D)** Panels show nitrate dynamics with different levels of glucose amendments (red: CHL+, black: CHL−, points: data, lines: model fit), where linear dynamics (at 0mMC) transition into exponential dynamics (≥0.5mMC) supports carbon limitation of nitrate utilization. Lines are model predictions. **(E)** Nitrate reduction rates after amending soils with different concentrations of nutrients (three carbon sources, ammonium, sulfate, and phosphate). Points are the mean rates, estimated by linear regression, of triplicates with error bars indicating standard deviation. Carbon (succinate $\left({pK}_{a}=4.2\right.$ and 5.6), acetate $\left({pK}_{a}=4.75\right)$, and glucose) amendments increased the nitrate reduction rates starting from low concentrations (0.5CmM)). Carbon compounds are negatively charged when ${pK}_{a}<$ pH (here, the pH of soil 6 is 5.4). Ammonium, sulfate, and phosphate did not result in a similar increase in nitrate reduction. We cannot independently infer the ratio $r_{A}/r_{C}$ (Methods), model predictions are shown for $1<r_{A}/r_{C}<3$ (shaded region) with a line for $r_{A}/r_{C}=2$ (best fit). This ratio can be interpreted as the nitrate:carbon utilization ratio.

Previous studies have observed that increasing soil pH can enhance the availability of organic carbon in soils [47–50], and detailed investigations suggest this is due to ion exchange within the soil clay particles [49]. Thus, we hypothesized that the amount of nutrients released would be proportional to the quantity of either base (NaOH) or acid (HCl) added to the slurry. If so, the fold change in nitrate reduction rate, reflecting the growth of active biomass limited by this nutrient, should exhibit the same proportionality. This expectation is tested in Fig. 4A. We observe, across all soils, a data collapse in the increase in nitrate reduction rate with NaOH (light blue region). The trend is specific to phase II (Fig. S11A), and if the data are plotted against pH, the correlation becomes much weaker (Fig. S10). As further evidence supporting our hypothesis, we can leverage our 16S rRNA amplicon sequencing data, which included internal standards to measure taxa abundances in absolute units (see Methods). This enables us to observe the predicted increase in biomass, both at a coarse level (fold change in total biomass) and fine level (individual ASVs that responded to the amendment of nitrate); see Fig. S11. Together, these tests confirm the linear relationship between the released nutrients and the amount of NaOH added to the system.

The asymmetry between HCl and NaOH in Fig. 4A is significant. Under the established mechanism of ion-exchange-mediated nutrient release, adding ions releases nutrients adsorbed to the clay particles into the pore water, making them accessible to microbes (Fig. 4B). Crucially, HCl and NaOH will release cationic and anionic nutrients, respectively (see SI for a more detailed discussion, Fig. S13B). Our observation that the hypothesized limiting nutrient governing Phase II dynamics is released proportionally to the amount of NaOH indicates it is anionic, with likely candidates including phosphates, sulfate, or carbon. Notably, measurements of water-soluble organic carbon (WSOC) at the endpoint increased linearly with NaOH added (Fig. S13C). This suggests that some WSOC is negatively charged (anionic) and that the growth-limiting nutrient might be WSOC, or concomitantly released nitrogen (N), sulfur (S), or phosphorus (P).

To identify the limiting nutrient we performed an amendment experiment on a representative soil. We amended a soil slurry without a pH perturbation applied with glucose (neutral), succinate (anion when $pH>{pK}_{a}=4.2$ or 5.6), acetate (anion when $pH>{pK}_{a}=4.75$), phosphate (anion), ammonium (cation), and sulfate (anion) added in varying concentrations (Methods, Fig. S14). With a single free parameter, our model predicted the nitrate utilization dynamics in soils amended with glucose (Fig. 4D). Similar results are found for other carbon sources, but not sources of N, S, or P (Fig. 4E). The single free parameter is the ratio $r_{C}/r_{A}$, which can be interpreted as a stoichiometry of carbon to nitrate utilization (Fig. 2), and matches expected properties of these compounds (e.g., our fitted value is highest for glucose and lowest for acetate, as expected). The result agrees with previous studies finding that nitrate respiration is carbon-limited [43, 51]. The amendment experiment provides an independent confirmation of the existence of a growth-limiting anion, which our model was able to predict directly from the functional data.

### Phase III arises from the rapid growth of rare taxa

Under large basic pH perturbations, all soils exhibited a sharp transition from linear to exponential nitrate consumption. Our model fits suggest interpreting these metabolite dynamics as resulting from a small initial functional biomass $\left(\tilde{x}(0\right)$) undergoing exponential growth due to excess nutrient $\gamma \tilde{C}(0)$ (Fig. 3C–F). To test this interpretation, we used the sequencing data to investigate the compositional changes occurring during this abrupt transition of functional dynamics (Phase III).

Remarkably, our sequencing measurements corroborate our model predictions by revealing a group of rare taxa that are enriched in Phase III. These are especially clear if ASVs are grouped at the phylum level, revealing that Firmicutes undergo explosive growth in this phase (10-fold enrichment at the aggregate phylum level, and several hundred-fold for multiple individual ASVs, particularly in the Bacilli genus; see Fig. S17). To perform this analysis systematically, we computed the fold change of each phylum’s absolute abundance across treatments (using no-growth CHL+ conditions as a reference). Non-negative matrix factorization (NMF) analysis of the growth fold values revealed that most of the variation in these data could be captured with just two axes of variation (Fig. S15B, Methods). Each of these was composed of only a few phyla, with one projecting predominantly on Proteobacteria and Bacteroidota, and the other almost exclusively on Firmicutes.

**Figure 5:**
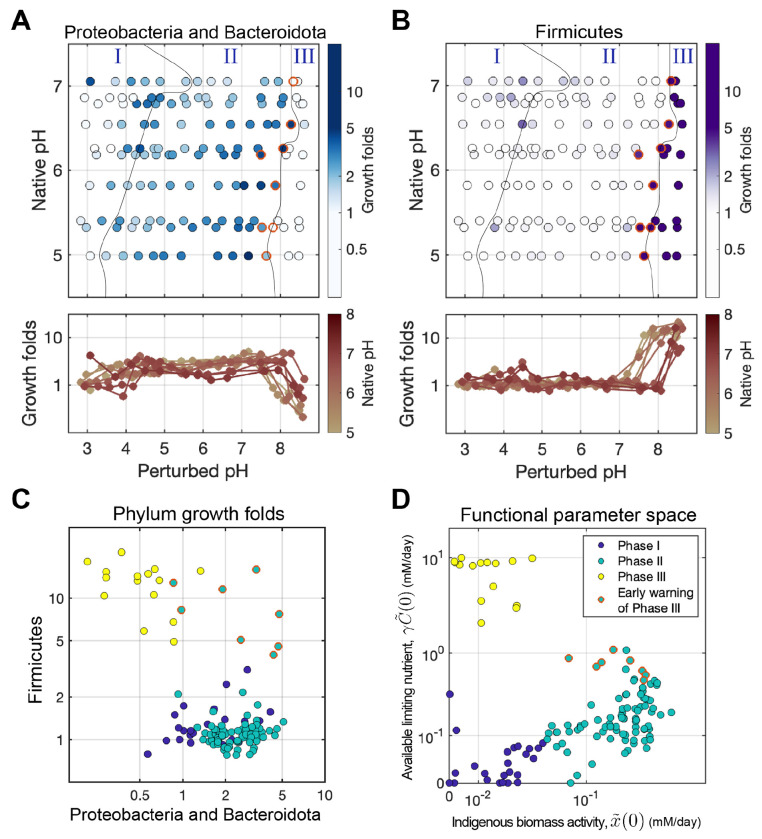
Phase III: Resurgent growth emerges from native population decline and rare taxa expansion. Global trends of growth in the phylum level across perturbed pH levels reveal taxonomic origins of the rapid growth in the resurgent growth phase (Phase III). 16S amplicon sequencing at the end of each incubation was used to identify amplicon sequence variants (ASVs) in CHL+/− conditions (Methods). ASVs were aggregated at the phylum level. For each phylum, a growth fold was computed as the ratio of abundances with/without growth $\left({Abs}_{CHL-}/{Abs}_{CHL+}\right)$. A statistical decomposition across all conditions identified three phyla that dominated abundance changes due to growth: Proteobacteria and Bacteriodota with similar changes, and Firmicutes. (see main text and Fig. S15). **(A)** Growth folds for the phyla Proteobacteria + Bacteroidota (combined abundance) indicated by the color for each native and perturbed pH condition. The growth declines at a basic pH threshold, mirroring the patterns observed in the fitted model parameter $\tilde{x}(0)$ (indigenous biomass activity, Fig 3C) at the phase II-III boundary). Phase boundary lines are those determined in Fig. 3). Line plots (lower panel) growth folds were plotted in logscale, color indicating native pH given in color bar. **(B)** Identical to (A) but showing growth folds (color) of Firmicutes increasing during the transition from Phase II to III, where pH perturbations are strongly basic. This mirrors the increase in inferred carbon concentrations $\gamma \tilde{C}(0)$ (Fig. 3). **(C)** Scatter plot of growth folds of Proteobacteria + Bacteroidota against Firmicutes. Points marked in red, associated with Phase II (also red in (D)), exhibit high growth of both Proteobacteria + Bacteroidota and Firmicutes. For red points, Firmicutes abundances are an early-warning indicator of a phase transition. **(D)** Same plot as Fig. 3A of $\tilde{x}(0)$ verses $\gamma \tilde{C}(0)$ with points marked by the phase they belong to and red points indicating phase II conditions near the boundary between Phase II and III. Note these red circles are in phase II, but the Firmicutes abundances are high (panel **(C)**).

Fig. 5A–B demonstrate that the transition from Phase II to Phase III is associated with the decline of Proteobacteria and Bacteroidota, and the enrichment of Firmicutes. We find that in the Nutrient-limiting phase (Phase II), Proteobacteria and Bacteroidota increased their growth with increased pH, and then decreased towards the start of Phase III. This matches the growth behavior of indigenous functional biomass ($\tilde{x}(0)$) revealed by the model in Phase II (Fig. 3C). On the other hand, Firmicutes did not grow until a critical pH threshold between 7-8.5, which matches the exponential increase of biomass growth in Phase III (Fig. 3F). Importantly, the boundary between Phase II and III derived from the functional dynamics data (Fig. 3C & D), aligns with the shifts in growth responses of Firmicutes (Fig. 5B) and Proteobacteria/Bacteroidota (Fig. 5A). These growth patterns suggest that the functional phases are reflected by the changes in the identity of the phyla responsible for nitrate reduction. A more detailed analysis of the likely metabolic traits of these strains [52] suggests that the transition from Phase II to III is also accompanied by a shift from denitrification to DNRA which agrees with the fact that excess carbon favors DNRA [53](Figs. S18 S19).

#### Growth is an early-warning indicator of a phase transition

Intriguingly, we found that Firmicutes begin increasing at pH levels just below the transition from Phase II to III, thereby acting as ‘early warning indicators’ for the impending transition (red circles, Fig. 5D). Specifically, when we plot the growth folds of the Firmicutes versus Proteobacteria and Bacteriodota, we find that Firmicutes abundances begin to rise just prior to the system entering phase III as defined by nitrate utilization dynamics (Fig. 5C). This finding indicates that compositional data can be used to predict functional state transitions during environmental perturbations.

### Acidic perturbations in Phase I reduce functional biomass via death

In response to a short-term decrease in pH, the model indicates a reduction in indigenous functional biomass ($\tilde{x}(0)$) and a decrease in the availability of limiting nutrients (Fig. 3). Below a pH value of 3–5, depending on the soil’s native pH, nitrate reduction ceases (Phase I). We tested whether the sequencing data reflects the decreasing trend of functional biomass $\left(\tilde{x}(0)\right)$ with pH. We computed the fold-change in each Phylum’s endpoint absolute abundance in CHL+ conditions relative to abundances at the initial time point $T_{0}$ (‘survival fold’; Fig. S20A). This ratio reflects the change in abundance in the absence of growth, hence we regard this as a proxy for death. For all phyla except Firmicutes, we observed a consistent drop of survival folds during acidic perturbations (Fig. S20A). Furthermore, we confirmed that the survival folds exhibited an approximately linear relationship with the $\tilde{x}(0)$ values (Fig. S20B). These observations confirm the widespread decline of biomass in acidic conditions, likely via death and DNA degradation, except in taxa tolerant to short-term pH changes (Firmicutes, Fig. S20A). Thus, we conclude that acidic perturbations lead to widespread death, while basic conditions lead to selective growth (Fig. S15C).

### Long-term soil pH defines phase boundaries

Having characterized functional phases, we next sought to understand what controls the boundaries between phases. We observed that the native pH of the soil (long-term pH) determined the pH at which any given soil transitioned between functional phases (Fig. 3C & D). This result suggests that the soil communities are adapted to their long-term pH conditions [22, 45, 54]. We next sought to understand how the properties of each soil’s response to changing pH might have impacted the pH at the phase boundaries.

**Figure 6:**
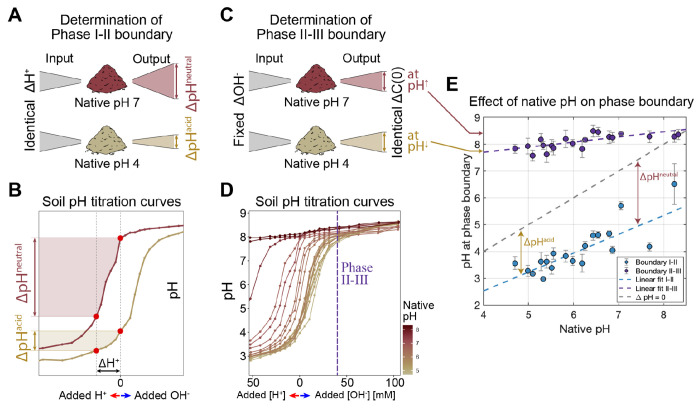
Phase boundaries are determined by long-term pH and history of pH variation. **(A)** Cartoon for how native pH impacts the pH of the Phase I and II boundary. The cartoon depicts how the identical amount of acid perturbations $\left(\Delta H^{+}\right)$ gives rise to larger changes in pH for neutral soils $\left(\Delta {pH}^{\text{neutral }}\right)$ than acidic soils $\left(\Delta {pH}^{\text{acidic }}\right)$ which arises due to differences in the location of the native pH on the titration curve shown in **B**. **(B)** Shows titration curves where the pH (y-axis) is measured after adding different amounts of acid or base (Methods) for neutral soil (dark brown) and acidic soil (light brown). The dashed vertical line at 0 indicates the pH with no acid/base perturbation. Due to the shape of these curves, if both soils are subjected to the same $\Delta H^{+}$ (bottom) the neutral soil experiences a larger change in pH (shaded regions). This suggests that acidic soils experience smaller pH fluctuations and therefore transition to phase I from II after smaller pH perturbations as shown in (**E**). **(C)** Cartoon for how native pH determines the pH of the Phase II and III boundary. The cartoon depicts how the fixed amount of added base (NaOH) results in an identical amount of released carbon (ΔC(0), Fig. 4). Large C(0) drives the transition from phase II to III. For a fixed addition of NaOH, more neutral soils reach higher pH again due to the shape of the titration curves as shown in D.). **(D)** Soil pH titration curves (identical to **C**) for all soils with different native pH levels. The vertical dashed line indicates the quantity of NaOH added to move from phase II-III. More neutral soils (darker colors) reach higher pH values for this fixed quantity of added NaOH. This correlates with the increasing pH at the Phase II-III boundary (purple points, (E)). **(E)** pH levels (y-axis) when the functional phase transitions from Phase II to I (blue points) and from Phase II to III (purple points) for soils from different native pH levels (x-axis). Phase boundaries are determined as the midpoint between the last pH perturbation in Phase I and the first in Phase II. Error bars represent the pH difference between these conditions. An identical strategy was used for phase II-III. The dashed blue line (Phase I-II boundary) and dashed purple line (Phase II-III boundary) are weighted least squares fits, with the weights inversely proportional to the error of each point. The dashed black line is slope 1, where the change in pH from native to the phase boundary is constant for all soils. Lines with a slope different from 1 indicate that the difference between native pH and pH at the phase boundary depends on the native pH of the soil. The slope of the blue dashed line is 0.7 (95% confidence interval: [0.44, 0.97].

To accomplish this, we quantified titration curves: changes in pH in response to acid/base additions, for each soil. The shape of the titration curve was similar across all soils (Fig. S23B&D), showing a plateau at low and high pH with a characteristic nonlinearity in between. As a result, acidic soils (with native pH near the lower plateau) were more strongly pH-buffered than the neutral soils (with native pH around the steepest portion of the nonlinearity; see Fig. 6B, Fig. S23A). This observation indicates that, at similar levels of acid addition, neutral soils would experience *larger drop in* pH than acidic soils $\left(\Delta {pH}^{\text{acidic }}<\Delta {pH}^{\text{neutral }}\right.$, Fig. 6A, B). We speculate that this makes communities in acidic soils less tolerant of acidic pH fluctuations, as they are less likely to natively experience large reductions in pH. This reasoning would help explain the behavior of the Phase I-II boundary shown in Fig. 6E (bottom), which shows that the best-fit line describing this boundary has a slope <1. A line of slope 1 (parallel to the grey dashed line in Fig. 6E) would indicate that entry into Phase I requires an acidic pH shift of a constant magnitude. Instead, we find that acidic soils tolerate smaller pH declines than neutral soils before entering phase I.

In contrast, we find that soils transition from Phase II to III, when carbon is in excess. From Fig. 4, we know that carbon is released proportionally to the NaOH added to the slurry (Fig. 6C). Accordingly, we find that a constant addition of NaOH drives the transition from Phase III to II (Fig. S24). However, due to the shape of the titration curves as seen in Fig. 6D, for a constant base amendment more neutral soils reach higher pH (dashed line Fig. 6E). Therefore, as expected from the titration curves, more basic soils transition to Phase III at higher pH (Fig. 6E).

Our sequencing data support the idea that variation in phase boundaries with native pH has a basis in the taxonomic composition of the microbiome. In natively more acidic soils, the Proteobacteria and Bacteroidota show better survival at lower pH (Fig.S21), while the growth initiation pH for Firmicutes in Phase III rises with the soil’s native pH (Fig. 5B), in line with the Phase II to III transition observed in functional measurements (Fig.6A). In addition, the strains that grow in phase III depend on the native pH of the soil (Fig.S17A). These findings suggest that prolonged exposure to a specific pH likely selects for specific taxa, thereby influencing the pH at which the community transitions between functional phases.

## Discussion

We showed that a simple mathematical model derived from quantitative measurements of metabolite fluxes delineates which processes are relevant for understanding the response of the soil to perturbations. Remarkably, the model does not attempt to account for all processes in the soils and instead captures the behavior of the entire community using a single effective biomass subjected to nutrient limitation. From this perspective, we identified functional phases that qualitatively govern the community’s response to environmental changes. For example, we discovered a regime of functional robustness (the Nutrient-limiting phase, Phase II) [4], where the indigenous biomass activity is robust to pH changes, as well as a regime where the rare biosphere determines community response (Phase III) to large basic perturbations [55].

### The significance of functional phases and their generalizability

The complexity of communities in the natural environment means that distilling which processes determine how a community responds to environmental variation is a challenge. Our study establishes the existence of functional phases where specific chemical, physiological, or ecological processes govern system response. This demonstrates that understanding the community response to perturbation may not require grappling with every metabolic process or interaction in the community, but only with a handful of key features. Our demonstration comes in the context of nitrate utilization and soil pH. However, we believe this study opens the door to asking whether similar functional phases describe community response to a suite of key perturbations including temperature or xenobiotics. Remarkably, a previous study of the response of soils to temperature revealed dynamics strikingly similar to Phase III at high temperatures and the asymmetric response in the Acidic death phase (Phase I) at low temperatures [56].

### Functional phases as guides for understanding complex omics data

Sequencing measurements of complex microbiomes result in datasets with tens or hundreds of thousands of variables - taxa, genes, or transcripts. Distilling some understanding from these data presents a huge challenge. The existence of phases guided our understanding of the dynamics of the >30,000 ASVs in our dataset by directing us to look for specific processes and timescales.

More broadly, the last decade has seen an explosion of methods for quantifying community dynamics and metabolism from transcriptomic and metagenomic measurements [57–59] to single-cell metabolomics [60] and quantitative stable isotope probes [61–63]. The challenge is to synthesize these data to achieve insights into dynamics and function. Our work illustrates the promise of an approach where we acquire large-scale quantitative measurements of metabolism at the whole community level, describe these dynamics mathematically, either phenomenologically or potentially new AI-driven methods [64], and then interpret the resulting model mechanistically. For example, in the Resurgent growth phase (Phase III), we expect native taxa to exhibit stress response and declining metabolic activity, and the converse for Firmicutes. Similarly, in the Acidic death phase (Phase I), we expect an increase in stress response genes during acidic perturbations. Thus the framework of phases suggests a route for leveraging new technologies for a deeper understanding of mechanisms in complex microbiomes.

### Physiological insights from constant utilization rates nutrient-limited environments

The linear dynamics of nitrate utilization observed here in the Nutrient-limiting phase (Phase II) have been previously observed [11, 40, 43, 65, 66] and attributed to carbon limitation [43]. Moreover, previous work supports the result that available organic carbon can be the limiting factor for nitrate utilization [10, 48, 51, 65, 67,–69].

How can limited carbon lead to a constant rate of nitrate reduction? Carbon is the electron donor for anaerobic respiration of nitrate which is the terminal electron acceptor. If carbon runs out we might expect that cells will run out of reductant to convert nitrate to nitrite, but this is not what we observe. One hypothesis is that cells internally store carbon to regenerate reductant for nitrate reduction [70]. To test this hypothesis, we incubated individual denitrifying bacterial strains in minimal media supplemented with $2mM{NO}_{3}^{-}$ without exogenous carbon. The observed nitrate reduction dynamics (Fig. S6) are linear, mirroring our measurements in the slurry. The result suggests that a fixed quantity of biomass can reduce nitrate at a constant rate even when carbon is severely limiting. Although the exact physiology of this process, likely related to maintenance energy in the stationary phase, requires further exploration, our model incorporating the limiting nutrient term reveals how cells behave in the natural soil matrix. This underscores the importance of considering the natural nutrient environment when we perform experiments to understand communities.

### Unifying decades of prior work in a quantitative framework

Our study focused on 20 samples from a single agricultural site. However, the features of the three functional phases defined here are present in many previous studies performed on topsoils from distinct sites, strongly suggesting that these phases are a general feature of nitrate utilization and pH perturbations. For example, Nömmik observed metabolite dynamics consistent with a transition from phase II to III [71]. Parkin *et al.* observed a native pH dependent $\tilde{x}(0)$ with chloramphenicol applied [45], consistent with all three phases. Simek observed increasing nitrate utilization rates with time as pH increased, another phase II to III transition [72]. Anderson *et al.* observed increasing rates of nitrate utilization with increasing pH, and the recruitment of Firmicutes in very basic conditions [47]. These results show that the phases are very likely general and not specific to our study site or experimental protocol.

#### Direct and indirect effects of pH perturbation

It has been debated whether the indirect effect of pH on nutrient availability is as important as the direct effect of pH on physiology [20]. Our results answer this question because the model enables us to quantify both the pH’s indirect effect on the growth-limiting nutrient (changes in $\tilde{C}(0)$) and its direct effect on indigenous functional activity that reflects physiology (changes in $\tilde{x}(0)$). In the Nutrient-limiting phase (Phase II), the indirect pH effect (changes in $\tilde{C}(0)$) is more important in determining the nitrate reduction rate because the indigenous functional biomass $\left(\tilde{x}(0)\right)$ is stable in this phase.

#### Optimal pH and long-term adaptation

Due to the agricultural importance of nitrate utilization, it has been debated whether soils exhibit an optimal pH for denitrification [72]. Previous studies have demonstrated the pH level associated with the highest rate of denitrification closely aligns with the native soil pH [45, 72] over short timescales (<3 hours, Table S2). Other studies observed a shift of optimum pH to more neutral values on longer timescales [72]. Our study reconciles these outcomes and elucidates the underlying cause. The fastest nitrate utilization occurs near the native pH of the soil on short timescales (Fig. 1B). This is consistent with our results because the pre-existing functional biomass $\left(\tilde{x}(0)\right)$ is the largest near the native pH (Fig. 3C&E). However, basic pH perturbations release carbon (C(0)), driving growth and faster reduction in alkaline conditions. Furthermore, with pH perturbations over 8 and long enough timescales (>12 hours), the growth of rare taxa drives fast nitrate reduction. As a result, the optimal pH depends on the time at which the measurement is made.

### Phases meet environmental fluctuations: origins of microbial diversity in nature

For large basic perturbations, the abundant native taxa could no longer perform nitrate reduction, while the rare biosphere grew rapidly to reduce nitrate (Phase III), acting as the source of functional resilience in the community. The adaptation of rare taxa in extreme environments suggests that there might be a trade-off between stress resistance and fast growth [73]. Rare taxa may specialize in surviving under extreme stress conditions (e.g., Firmicutes, Fig. S20), but perform little metabolic activity when the environment is near its native state. Conversely, dominant taxa near native environmental conditions (e.g., Proteobacteria) may specialize in faster growth rates when the nutrient becomes available but have limited ability to persist in stressful environments. These observations give rise to a picture where rare taxa are sustained by the presence of environmental fluctuations that transiently provide an opportunity to exploit resources [74].

Soil pH can change daily due to plant exudates (shifts of 0.4 in 12hrs), seasonally due to changes in rainfall and temperature (1–1.5 pH units), and through agricultural practices [75–77]. The titration curves gave insights into the amplitude of pH fluctuations the community experiences in nature (Fig. 6B). These observations place taxa’s distinct physiology and environmental fluctuations at the center of understanding the origin and structure of phases and therefore the metabolism of natural microbial communities. While physiology has experienced a renaissance of late, with quantitative approaches providing key insights [78], we know comparatively little about the role of natural environmental fluctuations in the wild. Our results suggest that understanding the dynamics and origins of these fluctuations could provide deep insights into the responses of complex communities to environmental change.

## Methods

### Sample collection, site description, and soil characterization

Twenty topsoils were sampled across a range of pH values (4.7–8.32) from the Cook Agronomy Farm (Table S3). The Cook Agronomy Farm (CAF, $46.78^{\circ }N,117.09^{\circ }W,800\;m$ above sea level) is a long-term agricultural research site located in Pullman, Washington, USA. CAF was established in 1998 as part of the Long-Term Agroecosystem Research (LTAR) network supported by the United States Department of Agriculture. Before being converted to an agricultural field, the site was zonal xeric grassland or steppe. CAF operates on a continuous dryland-crop rotation system comprising winter wheat and spring crops. CAF is located in the high rainfall zone of the Pacific Northwest region and the soil type is classified as Mollisol (Naff, Thatuna and Palouse Series) [79]. Sampling occurred from September 8-12, 2022, post-harvest of spring crops, to reduce plant’s impact on soil microbial communities. This period was during the dry season preceding the concentrated autumn rainfall.

Topsoils were collected from the eastern region of the CAF at a depth of 10-20 cm, other than Soil 1&2 (depth of 0–10 cm). Eastern CAF practices no-tillage which eliminates soil inversion and mixing of the soil surface to 20 cm. The N fertilizer in this field has been primarily deep banded to depths of approximately 7 to 10 cm during the time of application, which creates a spike of nitrate resource in the soil depth we sampled. Each soil sample was obtained by cutting down through the hardened dry soil with a spade in a circular motion to create a cylindrical cake of soil of radius 1020 cm until the desired depth. Each soil sample was not merged from sampling multiple replicates due to differences in pH in different locations. Samples were collected within a diameter of 500 m within the CAF to minimize the variation of edaphic factors other than pH. The large variation in soil pH comes from the long-term use of ammoniacal fertilizers and associated N transformations, which may undergo nitrification resulting in the release of H ions. In combination, spatial pH variation increases with field-scale hydrologic processes that occur under continuous no-tillage superimposed over a landscape that has experienced long-term soil erosion.

To maximize the coverage of sampled native pH, we used a portable pH meter (HI99121, Hanna Instruments, Smithfield, RI, USA) to directly measure and estimate the soil pH without having to make slurry on site to determine whether to sample the soil before sampling. For accurate pH values, pH was measured in the laboratory using a glass electrode in a 1:5 (soil to water w/w) suspension of soil in water (protocol of International organization for standardization, ISO 10390:2021), where 7 g of soil was vortexed with 35ml of Milli-Q filtered water, spun down, and filtered with (0.22μm) pore size. With these pH values, we selected 20 topsoil samples that are well spread across a range of pH from 4.7–8.32 with intervals of 0.1-0.6. Twenty soil samples were sieved (<2 mm), removed of apparent roots and stones, and gravimetric water content was determined ($\left.105^{\circ }C,24\;h\right)$. The sieved samples were stored in the fridge for 0-3 months until the incubation experiment. For sequencing the initial community, subsamples were stored in $-80^{\circ }C$ until the DNA extraction. The twenty soils were sent to the Research Analytical Laboratory (University of Minnesota, USA) to measure soil texture (soil particle analysis; sand, silt, clay composition), total carbon and nitrogen, and cation exchange capacity. The soils were also sent to Brookside Laboratories, Inc. (New Bremen, OH, USA) for a standard soil analysis package (Standard Soil with Bray I phosphorus). Twenty soils had relatively similar edaphic properties: 5-9% gravimetric water content (g/drysoilg), soil texture of silty clay or silty clay loam with 0% sand and 32-43% clay, and C:N ratio of 12–16 with 1–1.9% total carbon (wt/wt) (Table S3).

### Soil rewetting, constructing soil pH titration curves, and pH perturbation experiments

To mimic the autumn rainfall in the Pacific Northwest region and minimize the effect of spiking microbial activity by rewetting dry soils [80], we rewetted the sieved soil for 2 weeks before the perturbation experiments at room temperature with sterile Milli-Q water at 40% of each soil’s water holding capacity. After resetting, a soil slurry was made by adding 2mM sodium nitrate solution to the soil (2:1w/w ratio of water to soil). The slurry was then transferred to 48-deep well plates (2.35ml of slurry per well) for incubation under anaerobic conditions ($950RPM,30^{\circ }C$) for 4 days. Anaerobic incubation was performed in a vinyl glove box (Coy Laboratory Products 7601-110/220) purged of oxygen with a 99%/1% N2/CO2 gas mixture, where the gaseous oxygen concentration was maintained below 50ppm to prevent aerobic respiration [29].

To perturb the soil pH to desired levels, we constructed each soil’s pH titration curve for the 20 soils with varying native pH to know exactly how much acid or base to add to each soil sample. To do so, separate from the main pH perturbation experiment, we added 23 different levels of HCl (acid) or NaOH (base) in the slurry, final concentrations ranging from 0-100mMHCl or NaOH. We additionally tested whether the anion of acid $\left({Cl}^{-}\right)$ or the cation of base $\left({Na}^{+}\right)$ had a distinctive effect on the nitrate reduction dynamics, which was not the case (for results, see Fig. S12 and SI). We colorimetrically measured the pH (see section below) immediately after and 4 days after adding each well’s designated amount of acid/base. Due to natural soil’s buffering capacity, it takes 1-2 days to stabilize its pH level. Thus, we used the endpoint (after 4 days) pH measurements for all pH perturbations. We did a spline interpolation on the titration data points, plotting endpoint pH (y-axis) against acid/base input (x-axis), to compute how much HCl and NaOH needs to be added to the soil to obtain 13 different levels of pH with ≈0.4 intervals ranging from pH3 to 9, including the pH level without the addition of any acid or base. For Soil 19 and Soil 20, we had only 7 and 3 perturbed pH levels respectively, because the strong buffering capacity of these soils (native pH over 8) limited the range of pH perturbation.

For the main pH perturbation experiment, the computed levels of concentrated HCl or NaOH were added to the slurry in the 48-deep well plate with and without chloramphenicol treatment for each perturbed pH level in triplicates. The plates were immediately transferred to the shaking incubator (950 RPM in Fisherbrand Incubating Microplate Shakers 02-217-759, 3 mm orbital radius, $30^{\circ }C$) inside the anaerobic glove box and incubated for 4 days. For chloramphenicol-treated (CHL+) samples, we added concentrated chloramphenicol solution to the slurry to obtain a final concentration of 1 g/L. To gauge the effect of the 2mM nitrate, we had no-nitrate controls (0mM nitrate) for both CHL+/− treatments in the unperturbed pH conditions. With antifungal cycloheximide controls (200 ppm) for all 20 soils, we confirmed that fungal activity minimally affects nitrate utilization dynamics (Fig. S4). We also confirmed that abiotic nitrate/nitrite reduction does not occur by measuring metabolic dynamics of autoclaved soil ($\left(120^{\circ }C,99\right.$ minutes, autoclaved 5 times every 2 days) (Fig. S5). To offset the effect of increasing metabolite concentration due to evaporation throughout the 4-day incubation period, we used the wells with just 2mM nitrate, nitrite, and ammonium solutions to correct for evaporation in the slurry samples for every time point. The values of the gravimetric water content of each soil were taken into account to correct for the dilution of 2mM nitrate due to moisture in the soil. After the incubation, the plates were stored in $-80^{\circ }C$ for sequencing endpoint communities.

### Time-series slurry sampling, extraction, and colorimetric assays to measure nitrate, nitrite, ammonium, WSOC, and pH

To obtain the metabolic dynamics, we subsampled 60μL of the slurry into 96-well plates 10 times throughout 4 days (0, 4, 8, 19, 25, 31, 43, 55, 67, 91 hrs), where the initial time point $\left(T_{0}\right)$ is the time of pH perturbation and the start of anaerobic incubation. To measure nitrate and nitrite dynamics, extracts were prepared from the sampled slurries by adding and vortexing 2 minutes with 90μL of 3.33MKCl solution (final concentration of 2MKCl) and centrifuging at 4000rpm for 5 minutes. The supernatant was filtered at 0.22μm with a vacuum manifold to remove soil particles that could interfere with colorimetric assays. Concentrations of nitrate and nitrite in the extracts were determined colorimetrically using the Griess assay [81] and vanadium (III) chloride reduction method, following the protocol outlined previously [29]. We confirmed that 95%-99% of the nitrate in the soil can be accurately retrieved and detected using this method, as verified by nitrate spike-in and extraction experiments in the soil. For a subset of 20 soils (Soil 1, 5, 12, and 17), the ammonium dynamics were measured colorimetrically using the Salicylate-hypochlorite assay from the soil extracts [82]. Chloramphenicol treatments in the samples (CHL+) led to consistent detection of 0.5 $mM{NH}_{4}^{+}$ due to its N-H moiety. The salicylate-hypochlorite assay is also affected by the amount of base (NaOH) in the samples, resulting in slightly lower detection of chloramphenicol in the CHL+ samples ($\left(0.45mM{NH}_{4}^{+}\right.$ in 100mMNaOH perturbations). Taking advantage of these control measurements, we used the constant ${NH}_{4}^{+}$ levels in the controls without $2mM{NO}_{3}^{-}$ (No Nitrate controls) in the CHL+ samples for each soil to offset the NaOH effect in the CHL− samples and subtracted ${NH}_{4}^{+}$ levels caused by chloramphenicol in CHL+ samples.

For water-soluble organic carbon (WSOC) measurements, we subsampled 60μL of the slurry into 96-well plates at $T_{0}$ and endpoint (4 days). Then, soil extracts were prepared by adding, vortexing with 90μL Milli-Q water, centrifuging at 4000rpm for 5 minutes, and 0.22μm filtering the supernatant. Concentrations of the organic carbon in the supernatant were measured colorimetrically by the Walkley-Black assay, which uses dichromate in concentrated sulfuring acid for oxidative digestion [83]. We subtracted 0.4Cmg/ml from the CHL+ samples because chloramphenicol gave rise to a measured value of 0.4WSOCCmg/ml without additional carbon. For pH measurements, we subsampled 100μL of the slurry into 96-well plates at $T_{0}$ and the endpoint. Then, soil extracts were prepared by adding, vortexing with 150μLKCl solution (final concentration of 1MKCl), centrifuging at 4000rpm for 5 minutes, and 0.22μm filtering the supernatant. pH of the 120μL supernatant was determined colorimetrically by adding 4ul of the multiple indicator dye mixture via the protocol described previously [84]. The reason we used 1MKCl method for pH measurement (ISO 10390:2021) was that, contrary to the KCl method, the $H_{2}O$ method (using water instead of 1MKCl) resulted in a highly yellow coloration of the supernatants in strong basic perturbed samples, which interfered with the wavelength of the colorimetric pH assay. For samples of pH outside the range of the assay (below pH3 and above pH 9), we used a pH micro-electrode micro (Orion 8220BNWP, Thermo Scientific, Waltham, MA, USA).

### DNA extraction with internal standards, library preparation, and 16s rRNA amplicon sequencing

We performed 16S amplicon sequencing on half of all samples: 10(3,5,6,9,11,12,14,15,16, 17; Table S3) out of 20 soils were sequenced before perturbation and at the endpoint in both CHL+ and CHL− conditions, totaling 1,085 amplicon sequencing measurements. Genomic DNA was extracted from 500μL aliquots in a combined chemical and mechanical procedure using the DNeasy 96 PowerSoil Pro Kit (Qiagen, Hilden, Germany). Extraction was performed following the manufacturer’s protocol, and extracted DNA was stored at $-20^{\circ }C$. To estimate the absolute abundance of bacterial 16S rRNA amplicons, we added known quantities of genomic DNA (gDNA) extracted from *Escherichia coli* K-12 and *Parabacteroides* sp. TM425 (samples sourced from the Duchossois Family Institute Commensal Isolate Library, Chicago, IL, USA) to the slurry subsamples before DNA extraction. Equal concentrations of gDNA from these two strains were added. Both strains have identical rRNA copy numbers of 7 and comparable genome sizes of approximately 5000 kb. DNA Library preparation was performed using the 16S Metagenomic Sequencing Library Preparation protocol with a 2-stage PCR workflow (Illumina, San Diego, CA, United States). The V3–V4 region was amplified using forward primer 341-b-S-17 (CCTACGGGNGGCWGCAG) and reverse primer 785-a-A-21 (GACTACHVGGGTATCTAATCC) [85]. We confirmed using gel electrophoresis that the negative samples containing all reagents did not show visible bands after PCR amplification. Sequences were obtained on the Illumina MiSeq platform in a 2×300bp paired-end run using the MiSeq Reagent Kit v3 (Illumina, San Diego, CA, United States) with 25% PhiX spike-ins. A standardized 10-strain gDNA mixture (MSA-1000, ATCC, Manassas, VA, USA) was sequenced as well to serve as a positive control, which was confirmed to have relatively uniform read counts after assigning taxa.

### Model and fitting

#### Consumer-resource model

Consider a consumer-resource model with one consumer variable (functional biomass x(t), OD/biomass) and two resource variables (nitrate A(t) and carbon-nutrient C(t),mM), which evolves in time (t, day). The ordinary differential equations (ODEs) of the consumer-resource model can be expressed as:

(1) $$\dot{A}(t)=-r_{A}x(t)\frac{A(t)}{A(t)+K_{A}},\;\dot{C}(t)=-r_{C}x(t)\frac{C(t)}{C(t)+K_{C}}\;\dot{x}(t)=\gamma x(t)\frac{A(t)}{A(t)+K_{A}}\frac{C(t)}{C(t)+K_{C}}.$$

The first two equations of (1) represent the resource consumption rates, which are determined by the functional biomass (x, biomass), the maximum consuming rates per unit biomass $\left(r_{A}\right.$ and $r_{C}$, mM/biomass/day), and the Monod functions $\left(A/\left(A+K_{A}\right)\right.$ and $C/\left(C+K_{C}\right)$, dimensionless). Here assume the affinities $\left(K_{A}\right.$ and $\left.K_{C},mM\right)$ to be fixed and small. So the Monod functions can be deduced to 1 when $A\gg K_{A}$ or $C\gg K_{C}$, and can be deduced to 0 when A→0 or C→0. The third equation represents the growth of functional biomass, which is determined by the maximum growth rate per biomass (γ,1/day) and the multiplication of two Monod terms indicating the fact that nitrate and carbon are non-substitutable (electron acceptor and donor respectively). The growth is exponential $\left(x(t)=x(0)e^{\gamma t}\right)$ when both $A\gg K_{A}$ or $C\gg K_{C}$, but growth stops when either A→0 or C→0. Therefore, in this model, the growth of biomass is limited by both resources, but the consumption of one resource can continue when the other resource runs out and the biomass growth stops. For example, we believe this happens when C→0 in Phase II and the consumption of A continues (Fig. 2).

#### Solution for nitrate dynamics

To find the solution for nitrate dynamics, we rescale the equations by combining parameters: $\tilde{x}=$ $r_{A}x,\tilde{C}=Cr_{A}/r_{C},{\tilde{K}}_{C}=K_{C}r_{A}/r_{C}$. Therefore, the equations become:

(2) $$\dot{A}(t)=-\tilde{x}(t)\frac{A(t)}{A(t)+K_{A}}\;\dot{\tilde{C}}(t)=-\tilde{x}(t)\frac{\tilde{C}(t)}{\tilde{C}(t)+{\tilde{K}}_{C}}\;\dot{\tilde{x}}(t)=\gamma \tilde{x}(t)\frac{A(t)}{A(t)+K_{A}}\frac{\tilde{C}(t)}{\tilde{C}(t)+{\tilde{K}}_{C}}$$

In the rescaled equations (2), the parameters and variables all have units of rates (nitrate per time): $[\tilde{x}]=mM/$*day* and $[\tilde{C}]=\left[{\tilde{K}}_{C}\right]=mM$. Therefore, the solution of nitrate dynamics only depends on three parameters $\left(\gamma ,K_{A},{\tilde{K}}_{C}\right)$ and three initial conditions $\left(A_{0},\tilde{C}(0),\tilde{x}(0)\right)$. Since the affinities are very small $\left(K_{A}\approx 0.01\;mM,{\tilde{K}}_{C}\approx 0.01\;mM\right.$), the solution of biomass approximately equals to $\tilde{x}=\tilde{x}(0)e^{\gamma t}$ before the time at which growth stops $t^{*}$. So the resource dynamics before $t^{*}$ are approximately $A=A_{0}-\tilde{x}(0)\left(e^{\gamma t}-1\right)/\gamma$ and $\tilde{C}=\tilde{C}(0)-\tilde{x}(0)\left(e^{\gamma t}-1\right)/\gamma$. Accordingly, the time at which growth stops is given by $t^{*}=log\left(min\left(A_{0},\tilde{C}(0)\right)\gamma /\tilde{x}(0)+1\right)/\gamma$. If $\tilde{C}(0)<A_{0}$, the nitrate dynamics after $t^{*}$ and before running out are given by $A=A\left(t^{*}\right)-(\gamma \tilde{C}(0)+\tilde{x}(0))\left(t-t^{*}\right)$. As a result, the nitrate consumption rate after $t^{*}$ is $\gamma \tilde{C}(0)$ larger than the initial rate $\tilde{x}(0)$.

#### Least-square fitting scheme

To infer the model parameters from the metabolite measurement, we use the least-square fitting scheme to find the closest dynamic curves to the time-series data. Our metabolite measurement including the time points $\left({\underline{t}}^{-}=\left[t_{1}^{-},t_{2}^{-},\ldots ,t_{N}^{-}\right]\right)$ and nitrate amount $\left({\underline{a}}^{-}=\left[a_{1}^{-},a_{2}^{-},\ldots ,a_{N}^{-}\right]\right)$ for each CHL− sample, and the measurements of ${\underline{t}}^{+}$ and ${\underline{a}}^{+}$ for a corresponding CHL+ sample. We set up the loss function as the mean-squared-error (MSD):

(3) $$L=\frac{1}{2N}\left(\sum_{k=1}^{N}{\left(A\left(t_{k}^{-}\right)-a_{k}^{-}\right)}^{2}+\sum_{k=1}^{N}{\left(A^{c}\left(t_{k}^{+}\right)-a_{k}^{+}\right)}^{2}\right)$$

Here the functions A(t) and $A^{c}(t)$ are theoretical solutions of the consumer-resource model (2) for CHL−/+ conditions, respectively. Because the nitrate dynamics A(t) and $A^{c}(t)$ are determined by the parameter set $\Theta =\left\{\tilde{x}(0),\tilde{C}(0),A_{0},A_{0}^{c},\gamma ,K_{A},{\tilde{K}}_{C}\right\}$, we minimize the loss function L(Θ) to get the optimal model parameters $\Theta^{*}$. We note to the readers that three parameters are fixed (γ= $4.8day^{-1},K_{A}={\tilde{K}}_{C}=0.01mM$) as justified by the sensitivity analysis in the following paragraph. Note, that these parameters were globally fixed across all the data. We also use different initial nitrate ($\left(A_{0}\right.$ and $A_{0}^{c}$) in the functions A(t) and $A^{c}(t)$. The optimization algorithm is the interior-point method which is built in the MATLAB fmincon function. The codes and data are available at https://github.com/SiqiLiu-Math/xxx. The fitting errors over all samples are shown in Fig. S7, in which the root-mean-squared-error (RMSE, $\sqrt{L\left(\Theta^{*}\right)}$) and the error per datapoint $\left(\left|A\left(t_{k}^{-}\right)-a_{k}^{-}\right|\right.$ or $\left.\left|A^{c}\left(t_{k}^{+}\right)-a_{k}^{+}\right|\right)$ are normalized by the input value of nitrate (2mM).

#### Sensitivity analysis on model parameters

$\gamma ,K_{A}$, and ${\tilde{K}}_{C}$ were globally fixed to one value across all data. Here we justify this decision. We analyzed the sensitivity of $\gamma ,K_{A}$, and ${\tilde{K}}_{C}$ on simulated dynamic data. To reflect the three typical dynamics (phases) observed from the measurement, we simulated three nitrate curves by setting up the initial conditions to be $\tilde{x}(0)=0.01,0.1,0.001mM/\mathrm{day}$ and $\tilde{C}(0)=0.005,0.05,2mM$, respectively. Other parameters are given by $A_{0}=A_{0}^{c}=2mM,K_{A}={\tilde{K}}_{C}=0.01mM,\gamma =$ $4day^{-1}$. We then used different fixed values of parameters to fit the three examples. In the first row of Fig. S8, we used different fixed γ values - from $\gamma =2day^{-1}$ to $\gamma =6day^{-1}$ - to fit three simulations. We demonstrate very small mismatches (RMSE <5%) from these variations of parameter values, which are almost invisible in Phase I and Phase II fittings. In the second and the third row of Fig. S8, we use different fixed $K_{A}$ and ${\tilde{K}}_{C}$ values - from $10^{-4}mM$ to 1mM - to fit three simulations. When $K_{A}<0.1mM$ or ${\tilde{K}}_{C}<0.1mM$, the mismatches were again very small ( RMSE <1%) and invisible. These results indicate that the fixed values of $\gamma ,K_{A}$ and ${\tilde{K}}_{C}$ are insensitive in large ranges.

#### Determination of phase boundary with model parameters

To define the phase boundaries, we examined the distributions of each parameter’s value. $\tilde{x}(0)$ had a bimodal distribution (Fig. S9A). This bi-modality becomes more evident when we separately observe its distribution from the left half (perturbed pH<4) and right half (perturbed pH>6) of the parameter space displayed in the perturbed pH vs. native pH grid in Figure 3C (Fig. S9B). Therefore, we set the threshold for the $\tilde{x}(0)$ boundary where these two modes are evidently separated $\left(\tilde{x}(0)=0.05\right)$. The distribution of $\gamma \tilde{C}(0)$ exhibited a significant mode around 0, prompting us to set the threshold $\left(\gamma \tilde{C}(0)=1.5\right)$ at the tail region, where the $\gamma \tilde{C}(0)$ threshold also separated the Phase III samples in the top-left quadrant of the $\tilde{x}(0)$ vs. $\gamma \tilde{C}(0)$ scatter plot (Fig. 3A). The separation of Phase I and Phase II data points may not be clear cut in the $\tilde{x}(0)$ vs. $\gamma \tilde{C}(0)$ scatter plot (Fig. 3A). However, when we plot $\tilde{x}(0)$ of individual soils from different native pH levels (Fig. S9E), especially in the natively acidic soils, the transition from Phase II (large $\tilde{x}(0)$) to Phase I ($small\tilde{x}(0)$) is evident going towards more acidic pH perturbations because of the large $\tilde{x}(0)$ levels sustained over a wide pH range in Phase II.

### Sequence data analysis

#### Sequencing data preprocessing and assigning taxonomy to ASVs with DADA2

Raw Illumina sequencing reads were stripped of primers, truncated of Phred quality score below 2, trimmed to length 263 for forward reads and 189 for reverse reads (ensuring a 25-nucleotide overlap for most reads), and filtered to a maximum expected error of 4 based on Phred scores; this preprocessing was performed with USEARCH ver. 11.0 [86]. The filtered reads were then processed with DADA2 ver. 1.18 following the developers’ recommended pipeline [87]. Briefly, forward and reverse reads were denoised separately, then merged and filtered for chimeras. For greater sensitivity, ASV inference was performed using the DADA2 pseudo-pooling mode, pooling samples by soil. After processing, the sequencing depth of denoised samples was $10^{4}-10^{6}$ reads per sample. Low-abundance ASVs were dropped (≲10 total reads across all 1085 samples), retaining 34 696 ASVs for further analysis. Taxonomy was assigned by DADA2 using the SILVA database ver. 138.1, typically at the genus level, but with species-level attribution recorded in cases of a 100% sequence match.

#### Computing absolute abundance with internal standards of each ASV per sample

As an internal control, we verified that the ASVs corresponding to the two internal standard genera *Escherichia-Shigella* and *Parabacteroides* were highly correlated with each other as expected (person correlation ρ=0.94). These ASVs were removed from the table and combined into a single reference vector of ”spike-in counts”. The spike-in counts constituted 8.9±8.8% of the total reads in each sample. For downstream analysis, the raw ASV counts in a sample were divided by the spike-in counts of the internal standard per sample to obtain the absolute abundance of the ASV in a sample. Total biomass per sample was obtained by dividing the total raw read counts with the spike-in counts of the sample.

#### Differential abundance analysis to identify enriched ASVs

We conducted differential abundance analysis to statistically determine which amplicon sequence variants (ASVs) were significantly enriched in the Nutrient-limiting phase (Phase II) or the Resurgent growth phase (Phase III), respectively. To do so, we identified enriched ASVs for each perturbed pH condition in each native soil comparing endpoint chloramphenicol-untreated (CHL−) samples with endpoint chloramphenicol-treated (CHL+) samples. For each native soil, we then compiled a list of enriched ASVs by aggregating a union set of enriched ASVs across perturbed conditions that belong to Phase II (or Phase III). To remove ASVs that could be false-positive nitrate reducers, we similarly performed differential abundance analysis to identify ASVs that are enriched in no-nitrate controls (nitrate ${\;}^{-}$) by comparing endpoint chloramphenicol-untreated (CHL− & nitrate^−^) samples with endpoint chloramphenicol-treated (CHL+& nitrate^−^) samples. This filtering was done when inferring nitrate reducer biomass (Fig. S11C&D) and inferring the Phase III strains (Fig.S17). For each native soil, we only had nitrate^−^ controls for the condition without acidic/basic perturbation. We assumed that these enriched ASVs in no-nitrate conditions (NNresponders) without acid/base perturbation would also be false-positive nitrate reducers in other acidic or basic perturbation levels. For each native soil, we filtered out these false-positive NNresponders from the aggregated list of Phase II (or Phase III) enriched ASVs.

To identify the ASVs enriched for each perturbed pH level, it was necessary to determine what change in recorded abundance constitutes a significant change, relative to what might be expected for purely stochastic reasons. The relevant null model would combine sampling and sequencing noise with the stochasticity of ecological dynamics over a 4-day incubation, and cannot be derived from first principles. However, since all measurements were performed in triplicate with independent incubations, the relevant null model can be determined empirically. The deviations of replicate-replicate comparisons from 1:1 line were well-described by an effective model combining two independent contributions, a Gaussian noise of fractional magnitude $c_{\text{frac}}$ and a constant Gaussian noise of magnitude $c_{0}$ reads, such that repeated measurements (over biological replicates) of an ASV with mean abundance n counts are approximately Gaussian-distributed with a standard deviation of $\sigma \left(c_{0},c_{\text{frac }}\right)=\sqrt{{\left(c_{\text{frac }}n\right)}^{2}+c_{0}^{2}}$ counts. In this expression, $c_{\text{frac }}$ was estimated from moderate-abundance ASVs (>50 counts) for which the other noise term is negligible; and $c_{0}$ was then determined as the value for which 67% of replicate-replicate comparisons are within $\pm \sigma \left(c_{0},c_{\text{frac }}\right)$ of each other, as expected for 1-sigma deviations. This noise model was inferred separately for each soil and each perturbed pH level, as the corresponding samples were processed independently in different sequencing runs. For example, the parameters in Soil 11 were $c_{\text{frac }}=0.21\pm 0.04$ and $c_{0}=4.5\pm 0.7$ counts (Fig. S25).

The model was used to compute the z-scores for the enrichments of absolute ASV abundances in CHL− treatments against CHL+ controls (three independent z-scores from three replicate pairs; rep1-rep1, rep2-rep2, rep3-rep3). The median z-score was assigned to each ASV for each perturbed condition. In consideration of ASVs with 0 read count in either CHL−/+ samples, all raw ASV counts were augmented by a pseudocount of 0.5 and divided by the per-sample spike-in counts, yielding values that can be interpreted as the absolute biomass of each taxon (up to a factor corresponding to the copy number of the 16 S operon), measured in units where 1 means as many 16 S fragments as the number of DNA molecules in the spike-in. Significantly enriched ASVs were identified in each perturbed condition as those with z-scores greater than $z=\Phi^{-1}\left(1-\alpha /2/n_{ASV}\right)$, where $\Phi^{-1}(x)$ is the inverse CDF of the standard normal distribution, α=0.05, and $n_{ASV}$ as the number of nonzero ASVs in a given sample. This critical z-score $\left(z=4.2\right.$, when $n_{ASV}=2000$ for enriched ASVs and z=4.3, when $n_{ASV}=2500$ for filtering no-nitrate responders (NNResponders)) corresponds to a two-tailed Bonferroni-corrected hypothesis test at significance level α under the null hypothesis that counts in the CHL− and CHL+ conditions are drawn from the same distribution. These analyses were performed using custom MATLAB (Mathworks, Inc) and R scripts, which are available on the GitHub data repository for the present manuscript; for additional technical details, the reader is referred to the detailed comments in these scripts.

#### Non-negative matrix factorization (NMF) analysis on phylum-level growth folds

To analyze the abundance change at the phylum level, we compute the growth fold of each phylum at each condition. For each phylum, we compute the absolute abundance by aggregating the abundances of all ASVs within that phylum. Taking CHL+ abundance ${Abs}^{+}$ as the reference abundance and CHL− abundance ${Abs}^{-}$ as the endpoint abundance (where Abs denotes taxon abundance normalized to internal standard), the logarithm of the growth fold for phylum i and condition j is given by $g_{ij}=log\left({Abs}_{ij}^{-}+10^{-3}\right)-log\left({Abs}_{ij}^{+}+10^{-3}\right)$. Note that we use CHL+ abundance as reference instead of the initial abundance (at T0), to account for any effects on read counts unrelated to growth which would be common between CHL+ and CHL− conditions (e.g. direct effect of acid/base addition), allowing us to focus only on growth-mediated abundance changes. We also set all negative $g_{ij}$ to 0 since we are focusing on growth. For all 130 conditions (10 soils ×13 perturbations) and 40 phyla, the phylum-level growth folds G is a 40×130 matrix. For each phylum, the row vector ${\vec{g}}_{i}$ represents how it grows under different conditions (see Fig. S15 for the growth vectors of the first 10 phyla). In order to reduce the dimensionality of the growth matrix and extract the main features of the growth vectors, we use non-negative matrix factorization (NMF) to decompose the growth matrix G=W*H by factor 2. Here the feature matrix H is of size 2×130, and the two rows ${\vec{h}}_{1}$ and ${\vec{h}}_{2}$ are two modes of growth vectors (shown in Fig. S15B). Therefore, the growth vector of phylum i is thus decomposed as ${\vec{g}}_{i}\approx w_{i1}{\vec{h}}_{1}+w_{i2}{\vec{h}}_{2}$, while the weights $w_{i1}$ and $w_{i2}$ are from the 40×2 weight matrix W. The weights of all 40 phyla are plotted in Fig. S15B, showing that Firmicutes are mostly composed by the second mode ${\vec{h}}_{2}$ and other phyla are mostly composed by the first mode ${\vec{h}}_{1}$. Additionally, Bacteroidota and Proteobacteria show the most significance of the first mode. This decomposition keeps 93.36% of the original growth matrix.

#### Genotyping enriched ASVs with PICRUSt2

To understand what traits make Resurgent growth strains unique, we used PICRUSt2 ver. 2.5.2 [52] to infer putative genotypes of the enriched ASVs in the Resurgent growth phase (Phase III) (Fig. S18C). Using the script ”place_seqs.py” in the pipeline, we matched the representative 16S rRNA sequences of each amplicon sequence variant (ASV) to PICRUSt2’s curated reference genome database (multiple sequence alignment). Then, using the ”hsp.py” script and default parameters, we predicted KEGG orthologs (KO) abundance of each ASV with the matched reference genome (hidden-state prediction). To narrow down to KOs/genes related to denitrification and Dissimilatory Nitrate Reduction to Ammonium (DNRA), we focused on nitrate reductase in denitrification (*narG*/K00370, *narH*/K00371, *narI*/K00374, *napA*/K02567, *napB*/K02567) and nitrite reductase to ammonium (*nirB*/K00362, *nirD*/K00363, *nrfA*/K03385, *nrfH*/K15876). To track which KOs were enriched at which pH in the 89 families used in the peak pH analysis (see SI for peak pH analysis), we summed the relative abundance (reads / total reads of each perturbed pH level in CHL−) of the ASVs belonging to each family that possessed at least 1 predicted gene respectively for *narGHI*, *napAB*, *nirBD*, and nrfAH. Then, we plotted their relative abundance values across pH for all soils, indicated by the intensity of the poin’s colors (Fig. S18).

#### Taxonomy of growing strains in Phase III varies with soil native pH

To further investigate whether the taxonomic identity of Resurgent growth (Phase III) strains varies across natural pH environments, we performed a regularized regression analysis to see if we can predict the native pH level of the source soil from the presence or absence of taxa that grow in Phase III at the ASV, Species, Genus, Family, or higher taxonomic levels. The Resurgent growth strains were determined by the differential abundance analysis as described previously. Should our findings confirm that the prediction of native soil pH is feasible based on the taxonomic variation of these strains, it means that the strains responsible for growth in Phase III *depend on the long-term pH of the soil*. To do so, we used the sequencing data to build a matrix where the rows are samples (including three biological replicates) belonging to the Resurgent growth phase (Phase III), where each row has a corresponding native pH value of the original soil. There are 10 source soils with different native pH levels, and each soil has 3 to 6 pH perturbed samples (replicates) of which metabolite dynamics are classified as the Resurgent growth phase. The matrix’s columns are different taxa belonging to the identified Resurgent growth strains, either in ASV, species, genus, family, or higher levels. Each element of the matrix is 0 if absent and 1 if present in the sequencing data of the sample. Because the presence and absence of taxa can randomly depend on the random sampling depth of each sample, we test varying threshold values (0,0.001,0.005 relative abundance) to call the taxa present if their relative abundance is greater than the threshold (effects shown in Fig. S22F).

The regularized regression was performed to predict the native pH of the source soil of the samples from the presence and absence of taxa using only additive terms and LASSO regularization to avoid overfitting [88]. To estimate the regularization hyperparameter, tenfold cross-validation was performed on the samples from ten different soils with different native pH levels. All models were fit using the package glmnet in R version 4.1.4. To make predictions of the native pH, we used two strategies. First, ’in-sample’ predictions used all available data points to fit the regression coefficients and predicted native pH using those coefficients. Second, to ask whether we can still predict the native pH without the model seeing the samples belonging to that specific native pH level, we implemented a ’eave-one-soil-ou’ (LOSO) procedure where all the perturbed samples from one native soil were left out as a test set, and the model was trained on the remaining data to fit the regression coefficients. Then, we used the model to predict the native pH of the left-out samples (out-of-sample prediction). The observed versus predicted pH values are shown in the scatter plots (Fig. S22A). The prediction quality ($R^{2}$, coefficient of determination $=1-SS_{E}/SS_{T}$, sum of squares error over total sum of squares) was computed using the mean predicted and mean observed native pH levels for each soil (for different taxonomic levels and prediction strategies, see Fig. S22 E & F, negative $R^{2}$ values indicate the predictions are worse than just predicting the pH as the mean predicted pH). To ascertain that our high prediction quality was not a random artifact, we randomly permuted the native pH values of our soils 1000 times and then predicted in-sample the native pH to obtain $1000R^{2}$ values. We computed the threshold $R^{2}$ value that corresponds to the p-value of 0.05 (top 50th $R^{2}$ value out of 1000 instantiations) and compared it with the $R^{2}$ value that we have obtained with our true native pH predictions (Fig. S22G).

### Testing the effect of different bases and salts on nutrient release

To see the effects of different bases (NaOH and KOH) on nitrate reduction dynamics, we added different concentrations of NaOH and KOH (final concentration of 0,8,16,24mM in the slurry), following the same protocol previously described (without chloramphenicol), to measure the nitrate and nitrite dynamics (Fig. S12) using Soil 6 (Table S3). In addition, to test the effects of ${Na}^{+},K^{+}$, ${Cl}^{-}$ separately, we added different concentrations of salts (NaCl,KCl) (without chloramphenicol and without adding any acid/base) and measured the metabolite dynamics (Fig. S12).

### Nutrient amendment experiments with slurries

To experimentally determine what nutrient was limiting growth in the Nutrient-limiting phase, we conducted nutrient amendment experiments respectively with glucose, succinate, sodium acetate, ammonium chloride $\left({NH}_{4}Cl\right)$, monosodium phosphate $\left({NaH}_{2}{PO}_{4}\right)$, and sodium sulfate $\left({Na}_{2}{SO}_{4}\right)$ (for results, see Fig. 4D and Fig. S14). Among them, succinate (${pK}_{a}=4.21$ and 5.64, $25^{\circ }C$), acetate $\left({pK}_{a}=4.76,25^{\circ }C\right)$, and phosphate $\left({pK}_{a}=2.2,7.2\right.$, and $\left.12.4,25^{\circ }C\right)$ were strong candidates for the limiting nutrient according to our soil nutrient release hypothesis, due to their anionic nature in mid-range pH (5-7). The incubation was conducted following the same protocol using Soil 6 (Table S3) without chloramphenicol and without adding any acid/base. Concentrations were either in CmM,NmM,SmM, or PmM with final concentrations in slurry varying from 0 to 5mM, each in triplicates. Because we have previously tested the effect of ${Na}^{+}$ and ${Cl}^{-}$ to be negligible in nitrate dynamics, the effect of these amendments can be attributed solely to C/N/S/P nutrients other than ${Na}^{+}$ and ${Cl}^{-}$.

## Supplementary Material

1

## Data Availability

Data associated with this manuscript will be made publicly available at NCBI BioProject upon publication.
