# Supplementary material for: Functional regimes define the response of the soil microbiome to environmental change
Source: bioRxiv. 2024 May 28:2024.03.15.584851. Preprint. [Version 3] doi: 10.1101/2024.03.15.584851 (PMC10980070; doi:10.1101/2024.03.15.584851)
Supplement: Supplement 1 [file NIHPP2024.03.15.584851v3-supplement-1.pdf]

bioRxiv preprint doi: https://doi.org/10.1101/2024.03.15.584851; this version posted May 28, 2024. The copyright holder for this preprint (which was not certified by peer review) is the author/funder. This article is a US Government work. It is not subject to copyright under 17 USC 105 and is also made available for use under a CC0 license.

Supplementary Materials

A detailed description of the three functional regimes: the Acidic death regime (Regime I), Nutrient-limiting regime (Regime II), and Resurgent growth regime (Regime III)

By quantitatively distinguishing the impact of pH on the consumer side (microbial community, $\tilde{x}(0)$) and the resource side (growth-limiting nutrient, $\tilde{C}(0)$), we can ask the mechanism behind functional adaptation during different regimes. Regime II can be called the "Nutrient-limiting" regime. Within this pH range (Fig. 3C), conditions favor the resident population of nitrate reducers; hence it allows a large indigenous nitrate reducer population to perform nitrate reduction (large $\tilde{x}(0)$). This specific range of favorable pH levels is determined by the long-term exposure to the native pH of soils (Fig. 3C). In this regime, the increase of nitrate reduction rate is determined by the biomass growth from the available growth-limiting nutrient. Therefore, in Regime II, the adaptive strategy employed by the nitrate-reducing population is to utilize the pre-existing resident species which are rather robust to pH perturbations, and at the same time incrementally increase the resident's biomass as the resource availability changes with pH perturbations. Going back to the functional dynamics, that is the reason we see a relatively high slope of CHL+ conditions and a slight increase of denitrification rate in CHL- conditions ((b) and (e) of Fig. 3B), demonstrating that the resident nitrate reducers adapt to the new environment in a "nutrient-limiting" manner.

Regime III can be called the "Resurgent growth" regime. As the perturbed pH is increased from Regime II, there comes a critical pH of around 8 where the adaptive mechanism abruptly transitions. When the pH is perturbed beyond the critical point, the previously large biomass of the nitrate reducer population can no longer adapt and perform nitrate reduction ($\tilde{x}(0) \to 0$). On the resource aspect in Regime III, there is a surplus of limiting nutrients, and thus the system is no longer limited by C but limited by nitrate A. These two effects of short-term pH change (both on the consumer and resource aspect) set the stage for the "resurgence". A rare population, which we will investigate the composition later, appears to have a small biomass initially showing a flat

bioRxiv preprint doi: https://doi.org/10.1101/2024.03.15.584851; this version posted May 28, 2024. The copyright holder for this preprint (which was not certified by peer review) is the author/funder. This article is a US Government work. It is not subject to copyright under 17 USC 105 and is also made available for use under a CC0 license.

slope in both CHL- and CHL+ conditions but later grows exponentially to exponentially deplete the nitrate in Regime III (panel c, f of Fig. 3F). This shows that in Regime III, the adaptive mechanism of the community is to rely on the rare uprising nitrate reducer biomass to rapidly grow in the absence of nutrient limitation.

Regime I can be called the “Acidic death” regime. This regime is at the other end (acidic) of the Nutrient-limiting regime (Regime II). As the perturbed pH is decreased from Regime II, it transitions into a regime where the system fails to adapt. The boundary pH of Regime I and Regime II is influenced by the native pH of the soil, where relatively acidic soils have a lower boundary of pH 3 or 4 and relatively neutral soils have a higher boundary of pH 5 to 6. Similar to what happens when the community enters Regime III, the unfavorable pH diminishes the indigenous nitrate-reducing activity of the soil, indicated by the flat CHL+ dynamics in panels a, d of Fig. 3B. However, unlike Regime III, the perturbed pH does not make the growth-limiting nutrient superfluous but makes it unavailable, making the divergence of CHL- and CHL+ dynamics nonexistent (panel a, d of Fig. 3A). These two effects of pH perturbation make it extremely difficult for the community to adapt to the new environment of Regime III, hence the “Acidic death” regime. Another reason we call it that is to highlight the asymmetric effect of acidic and basic perturbations, which has been seldom acknowledged in the literature.

Detailed mechanism of nutrient release in soils due to change in pH

We turned to soil literature to develop a comprehensive mechanism for the nutrient release mechanism in soil [38, 39]. Soil comprises minerals, organic matter, water, and air. Minerals and organic matter form aggregated clumps of soil particles, categorized by size into sand, silt, and clay. Clay particles, the smallest among them, consist of layers of phyllosilicates. Each layer includes tetrahedral structures of Si^{4+} covalently bonded to four oxygens and octahedral structures of continuous Mg^{2+} or Al^{3+} covalently bonded to six hydroxides [82]. Due to this chemical structure, clay particles possess numerous electrostatically charged sites, including negatively charged (oxygen atom

bioRxiv preprint doi: https://doi.org/10.1101/2024.03.15.584851; this version posted May 28, 2024. The copyright holder for this preprint (which was not certified by peer review) is the author/funder. This article is a US Government work. It is not subject to copyright under 17 USC 105 and is also made available for use under a CC0 license.

and hydroxide) and positively charged [39] (shown as - and + sites in Fig. 4B). The clay particle's cation exchange sites are negatively charged and form ionic bonds with cations (positively charged ions), while the anion exchange sites are positively charged and bind to anions (negatively charged ions). Both cations and anions can serve as potential nutrient sources. When they are bound to the clay's exchange sites (brown section in Fig. 4B), they are protected from microbes. However, when they are released and dissolved in the pore water (light blue or pink section in Fig. 4B), they become available to the microbial community. To understand how NaOH or HCl impacts nutrient availability, it's essential to track whether cations and anions are bound to clay particles or dissolved in the pore water.

The literature on nutrients and pH in soils proposes the following mechanism for nutrient release (Fig. 4B, see more detailed cartoon Fig. S13B) [38, 39]. When NaOH is added to the soil solution, both Na^+ and OH^- ions (pH-mediated) act to release the anionic nutrients (case 2 of Fig. 4B). First, OH^- deprotonates ion exchange sites in the clay particles increasing the number of cation exchange sites (- charge) and decreasing the number of anion exchange sites (+ charged) reducing the capacity of the clay to hold anions. Secondly, Na^+ can either bind to the clay particle or remain in solution [38]. The (Na^+) that remains in solution increases the stability of released anions. Overall, increased OH^- increases the anionic nutrients available to the microbial community (Fig. 4B). The converse happens for HCl perturbations (case 1 of Fig. 4B).

Effect of base cation on nutrient release in soils

Nutrient release is not solely driven by OH^- ions. The base cation plays an important role, and thus whether the base cation prefers to be in the clay particle or the water solution can influence the amount of nutrients available to microbes. It is known that the bigger the size and greater the charge of the cation, the more selectively the cation binds to clay particles. For example, the cation's binding specificity to the clay particle instead of staying in the solution is in the order of $NH_4^+ > K^+ > Na^+ > Li^+$, divalent cations having greater binding specificity than monovalent

bioRxiv preprint doi: https://doi.org/10.1101/2024.03.15.584851; this version posted May 28, 2024. The copyright holder for this preprint (which was not certified by peer review) is the author/funder. This article is a US Government work. It is not subject to copyright under 17 USC 105 and is also made available for use under a CC0 license.

cations (e.g., $Ca_2^+ > K^+$) [39]. Therefore, when the amount of dissolved organic carbon (DOC) was measured after adding $Ca(OH)_2$ vs. KOH in equimolar hydroxide, adding KOH resulted in a much higher concentration of dissolved organic carbon (DOC) [38]. Because K^+ ions less specifically bind to the clay particle and more likely remain in the solution, it would stabilize the released anions better in the solution, presuming that the DOC is mostly anion due to many negatively charged moieties of O^-s. To check if there was a significant difference between monovalent cations, we compared NaOH and KOH treatments for basic perturbations. We found that there was no significant difference in the nitrate utilization rates in the CHL- condition when we added the same concentrations of NaOH and KOH, indicating that the amount of limiting nutrient released was similar (Fig. S12), although the stabilized endpoint pH was different. As a sanity check, we further tested KCl and NaCl treatments and found that K^+, Na^+, or even Cl^- (relevant in the HCl addition) ions themselves without OH^- did not affect nutrient release (Fig. S12), which agrees with previous findings [38].

Recapitulating linear dynamics with monoculture experiments without carbon

Our model and functional dynamics data suggest that the limited carbon leads to a constant rate of nitrate reduction. However, it is difficult to understand the mechanism behind this phenomenon, because if the organic carbon, an electron donor in the electron transport chain, is coupled to the reduction reaction of nitrate (terminal electron acceptor), the depletion of organic carbon will likely stop the nitrate reduction performed by the nitrate reductase enzymes. This will cause the nitrate reduction rate to be close to 0 rather than the observed constant rate. To resolve this contradiction, one hypothesis can be that the cells internally store carbon nutrients (electron donor) to power the electron transport chain (consuming nitrate) without needing to import external carbon nutrients to generate ATPs for the cell's maintenance energy. To test this hypothesis, we conducted monoculture experiments with a *E. coli* strain and a known denitrifier *Pseudomonas sp.* strain.

bioRxiv preprint doi: https://doi.org/10.1101/2024.03.15.584851; this version posted May 28, 2024. The copyright holder for this preprint (which was not certified by peer review) is the author/funder. This article is a US Government work. It is not subject to copyright under 17 USC 105 and is also made available for use under a CC0 license.

Culturing protocol

Strains were pre-cultured in two stages under aerobic conditions before being transferred to denitrifying (anaerobic) conditions for phenotyping. First, wells of a sterile 24-well plate (Thermo Scientific Nunc Non-Treated Multidishes) were loaded with $1.7\,\mathrm{mL}$ of R2B medium. Wells were inoculated with *E. coli* K12 and *Pseudomonas sp.* PDM04 [73] strains from glycerol stocks stored at $-80\,^{\circ}\mathrm{C}$. The plates were then sealed with a gas-permeable sterile membrane (Breathe-Easier, USA Scientific, 9126-2100). After sealing, the culture was incubated overnight at 0.5 rcf ($400\,\mathrm{RPM}$ in Fisherbrand Incubating Microplate Shakers 02-217-759, $3\,\mathrm{mm}$ orbital radius) and $30\,^{\circ}\mathrm{C}$ in aerobic conditions. These cultures reached saturation during this time. Second, wells of a sterile 24-well plate were loaded with $1.7\,\mathrm{mL}$ of defined media ($15\,\mathrm{mM}$ ammonium, $40\,\mathrm{mM}$ phosphate buffer with the final medium pH adjusted to 7.3, and trace metals and vitamins, as described in Ref [73]) with $25\,\mathrm{mM}$ succinate. Wells were then inoculated with $17\,\mu\mathrm{L}$ of the saturated R2B *E. coli* K12 and *Pseudomonas sp.* PDM04. After sealing, the cultures were incubated at 0.5 rcf and $30\,^{\circ}\mathrm{C}$ in aerobic conditions overnight. These cultures reached saturation during this time. Saturated defined media (DM) cultures were washed and normalized to a desired optical density (measured at $600\,\mathrm{nm}$) via dilution into pH 7.4 phosphate-buffered saline ($8\,\mathrm{g/L}$ H_2O, $0.2\,\mathrm{g/L}$ KCl, $2.68\,\mathrm{g/L}$ $Na_2HPO_4{\cdot}7H_2O$, $0.24\,\mathrm{g/L}$ KH_2PO_4).

Wells of a sterile 96-deep well plate (Axygen PDW20C) were loaded with carbon-free $1.2\,\mathrm{mL}$ DM supplemented with 2mM sodium nitrate which had been allowed to equilibrate in the anaerobic glovebox. These wells were inoculated in the glovebox with $12\,\mu\mathrm{L}$ of OD-normalized aerobic pre-cultures, resulting in starting ODs of 0.1 and 0.01. Additional wells were left blank as no-growth controls. Plates were sealed with a gas-permeable sterile membrane. Cultures were incubated at $30\,^{\circ}\mathrm{C}$ and shaken at $950\,\mathrm{RPM}$ (Fisherbrand Incubating Microplate Shakers 02-217-759 or Talboys Professional 1000MP, 3 mm orbital radius) for $72\,\mathrm{h}$. Optical densities of initial and endpoint anaerobic pre-cultures were measured using $300\,\mu\mathrm{L}$ of cultures in 96-well optical plates. Nitrate and nitrite concentrations were assayed over time via manual sampling and subsequent Griess assay

bioRxiv preprint doi: https://doi.org/10.1101/2024.03.15.584851; this version posted May 28, 2024. The copyright holder for this preprint (which was not certified by peer review) is the author/funder. This article is a US Government work. It is not subject to copyright under 17 USC 105 and is also made available for use under a CC0 license.

and vanadium (III) chloride reduction via the protocol described in Ref. [73].

Linear metabolite dynamics were recapitulated with monoculture experiments

Both *E. coli* K12 and *Pseudomonas sp.* PDM04 strains were able to reduce nitrate even without carbon in the culture media (Fig. S6A). The reduction rate was negligible for *E. coli* strain at a starting OD600 of 0.01 (optical density at 600nm). However, for the denitrifier *Pseudomonas sp.* PDM04 at a starting OD600 of 0.01, not only the rate of nitrate reduction was comparable to what we observed in soils at the Nutrient-limiting regime (Regime II), but the reduction dynamics were strikingly linear. This result directly demonstrates that nitrate reduction can proceed even when carbon is not exogenously available. Our observations are consistent with this hypothesis that the cells can internally store carbon and oxidize this carbon to provide electrons (NADH) to reduce nitrate to nitrite. If the nitrate reduction rate had increased, it would have meant that the functional biomass, or the quantity of nitrate reductase enzyme, increased. The nitrate reduction rates did not increase throughout the experiment (top panel in Fig. S6A). This supports the idea that cells are using nitrate to maintain biomass. Consistently, final OD600 measurements did not detect any significant increase from the initial OD600 as expected. We can now more confidently presume that soil microbial communities are also in the same maintenance state during the linear nitrate dynamics. In sum, these results suggest that the functional biomass in soils can utilize nitrate at a constant rate, even after external carbon is no longer available. Note our model assumes this to be the case (Fig. 2).

In the monoculture experiment, we observed biphasic behavior in the high initial OD600 condition. This phenomenon is challenging to interpret. In the starting OD600 of 0.1, the initial slopes of nitrate reduction dynamics are constant, then after some time, the rates decrease and remain constant (we will call this ”late slope”) until the end of the experiment (bottom panel of Fig. S6A). The linear dynamics observed in the ”late slope” again still recapitulate the linear dynamics we observed in the soils in the Nutrient-limiting regime, where the microbes could be using nitrate and

bioRxiv preprint doi: https://doi.org/10.1101/2024.03.15.584851; this version posted May 28, 2024. The copyright holder for this preprint (which was not certified by peer review) is the author/funder. This article is a US Government work. It is not subject to copyright under 17 USC 105 and is also made available for use under a CC0 license.

internally stored carbon to generate maintenance energy. In the soil experiments, the ”late slopes” were determined by the increased functional biomass in the model. However, in these monoculture experiments, it was difficult to understand what determines the late slope values. Their biomass had not changed from the beginning according to the endpoint OD600 measurements, hence requiring further investigation of the bacterial physiology. The initial slopes can be roughly explained by their starting biomass (Fig. S6B), where the initial slope for the OD600 0.1 condition was roughly 10 times greater than that for the OD600 0.01 condition, with the fitted initial slopes showing an increase factor ranging between 5 to 19 times.

Confirming the linear dependence between functional biomass and acid/base input

Although the linear relationship between acid/base input with the total biomass increase during the Nutrient-limiting regime (Regime II) corroborates our proposed nutrient release mechanism, to be more precise, we need to further show that the fold increase of the “functional” biomass is equal to the fold increase of nitrate reduction rate from the nitrate dynamics data. However, when we observe ASVs increasing in absolute abundance from the start to the end of the experiment we cannot assume all ASVs are performing nitrate reduction (for example, some may be growing via fermentation). To address this we filtered out the ASVs that are likely not nitrate reducers by removing ASVs that were enriched in no-nitrate conditions (dark grey NNresponders bar in Fig. S11D, no pH perturbation). To detect the fractional biomass that performs nitrate reduction, we used a differential abundance analysis to statistically determine which amplicon sequence variants (ASVs) were significantly enriched in each pH perturbed condition compared to the CHL+ counterpart serving as a baseline of no growth (see Materials and Methods for details). Then, we summed up the absolute abundance of these ASVs that we inferred as true nitrate-reducing biomass to obtain the functional biomass for each condition (red bar in Fig. S11D). By comparing the fold increase of these functional biomass values (endpoint/initial functional biomass), we showed that indeed the functional biomass increase and nitrate reduction rate increases are similar in different

bioRxiv preprint doi: https://doi.org/10.1101/2024.03.15.584851; this version posted May 28, 2024. The copyright holder for this preprint (which was not certified by peer review) is the author/funder. This article is a US Government work. It is not subject to copyright under 17 USC 105 and is also made available for use under a CC0 license.

soils (Fig. S11C). While some soils showed very close agreement between the inferred increase in functional biomass and increases in nitrate reduction rate (Soil 11, inset of Fig. S11C), for many soils the relationship was not quantitative. This discrepancy likely arises from the fact that we inferred the taxa that are not nitrate reducers from slurries where the pH was not perturbed. Thus the no-nitrate responders may be distinct as pH is perturbed and this may increase errors in our inference of changes in functional biomass.

Investigating the taxonomy, pH niche, and the phylogeny of the Resurgent growth strains

Determination of peak pH for each family

To elucidate the pH niche of each family in Fig. S18A, we analyzed the relative abundance of the chloramphenicol-untreated (CHL-) conditions of ASVs identified as being enriched in different pH levels (see Differential abundance analysis). Due to the challenge of visualizing a large number of ASVs, we aggregated the relative abundance of ASVs in the same family for each sample, visualizing the data at the family level. To get the representative relative abundance of the family in each perturbed pH level, we took the median of the relative abundance from three biological replicates. To incorporate abundance values at each perturbed pH from all soils with different native pH levels, we placed the perturbed pH values from all native soils from smallest to largest and then binned neighboring 2–4 perturbed pH values (depending on the total number of relative abundance values greater than 0) to compute the median relative abundance of each family within each bin corresponding to its mean pH value. Now, for each family, we have a median relative abundance value assigned to the mean pH of each bin. For each family, we ranked those median relative abundance values across perturbed pH and found the peak pH value with the highest relative abundance, as well as the second peak pH and its corresponding relative abundance. To reduce the number of families to plot, we chose 89 families that have relative abundance values at the second peak pH greater than 0.002 (= 0.2%). After aligning the 89 families with their peak pH from smallest (top of the plot, dark blue color) to largest (bottom of the plot, yellow color), we plotted a

bioRxiv preprint doi: https://doi.org/10.1101/2024.03.15.584851; this version posted May 28, 2024. The copyright holder for this preprint (which was not certified by peer review) is the author/funder. This article is a US Government work. It is not subject to copyright under 17 USC 105 and is also made available for use under a CC0 license.

ridge plot with the x-axis being perturbed pH level and height corresponding to the median relative abundance of each bin (Fig. S18A). The maximum heights of the ridge for each family were set to the same level by normalizing the maximum relative abundance for each family. Indeed, the family with peak pH over 8 were mostly Firmicutes phylum (Bacillaceae, Clostridiaceae, Paenibacillaceae, Caloramatoraceae, Peptostreptococcaceae, Lachnospiraceae), other than Yersiniaceae family which was Proteobacteria.

Constructing a phylogenetic tree with 16S rRNA sequences

To see whether there was phylogenetic convergence among strains with similar pH niches, we used the 16S rRNA sequences of the ASVs to construct a phylogenetic tree (Fig. S18B). To be consistent with the previous peak pH analysis, we used the ASVs that belonged to the 89 families in the previous analysis. We selected one ASV with the largest relative abundance from each family to represent the family and used its 16S rRNA sequence to construct the phylogenetic tree. The phylogenetic tree was constructed by approximating the Maximum likelihood tree with the General time reversible model in FastTree ver. 2.1.9 [83]. The tree was plotted using the plot.phylo function in ape package in R, each node (labeled with the classified genera or species name) colored by its peak pH.

Lower-level taxonomy and traits of the Regime III strains

To identify the specific taxa accountable for the emergence of Regime III at a finer taxonomic level, we conducted a differential abundance analysis that statistically determined which Amplicon sequence variants (ASVs) were significantly more abundant in Regime III CHL- samples, compared to CHL+ samples under same perturbed conditions (see Methods). Then, we aggregated the relative abundance of these differential ASVs (i.e., Regime III strains) to assess their contribution to the emergence of Regime III. Notably, their abundance began to rise between pH 7-8 (Fig. S17C), which aligns with or slightly precedes the transition between Regime II and III (Fig. S17D). This increase in relative abundance corresponded with the shift of the nutrient growth parameter $\gamma\tilde{C}(0)$

bioRxiv preprint doi: https://doi.org/10.1101/2024.03.15.584851; this version posted May 28, 2024. The copyright holder for this preprint (which was not certified by peer review) is the author/funder. This article is a US Government work. It is not subject to copyright under 17 USC 105 and is also made available for use under a CC0 license.

from zero (Fig. S17E). The analysis revealed that 10 families belonging to Firmicutes (Bacillaceae, Paenibacillaceae, Clostridiaceae, Caloramatoraceae, Peptostreptococcaceae, etc.) and 2 families belonging to Proteobacteria phylum (Legionellaceae and Yersiniaceae) were significantly enriched in Regime III (Fig. S17B). At the genus level, Bacillus, Clostridium, Paenibacillus, and others were identified as the primary contributors to Regime III (Fig. S17A).

We lastly sought to find distinct features of the Regime III strains that differentiated them from other strains to understand why these strains better adapt to perform nitrate reduction in high pH and high carbon conditions. To do so, we classified families by their peak pH obtained by finding the pH level at which its median relative abundance across soils was the highest across different perturbed pH levels (Fig. S17A). We indeed found that the Regime III families had distinct pH niches compared to other strains, having high relative abundance in basic pH (over 8) and in some cases acidic pH (less than 4) but remained rare ($< 0.1\%$) in the mid-range of pH 4-8. One can speculate that their ability to survive and persist in extreme pH perturbations (see Fig. S20A) may be because many taxa in the phylum Firmicutes are spore-forming bacteria species [84]. These strains did not cluster phylogenetically and were dispersed throughout the phylogenetic tree (Fig. S17B, see Methods).

pH titration curves and soil's native pH are shaped by soil's physicochemical properties

We've constructed pH titration curves for the 20 soils from different native pH levels (see Methods, Fig. 6B). Because we titrated both in acid and basic directions with H^+ and OH^- respectively, we unified the x-axis to OH^- (m mol) by shifting the curves to the right by 0.2 m mol, ensuring each curve starts at 0 m mol OH^-. We then fitted the pH titration curves with a logistic function with 4 parameters (a, x_{mid}, b, c) as below (visualized in Fig. S23B):

$$pH = \frac{a}{1 + e^{\frac{-(x - x_{mid})}{b}}} + c \tag{4}$$

bioRxiv preprint doi: https://doi.org/10.1101/2024.03.15.584851; this version posted May 28, 2024. The copyright holder for this preprint (which was not certified by peer review) is the author/funder. This article is a US Government work. It is not subject to copyright under 17 USC 105 and is also made available for use under a CC0 license.

Parameter x_{mid} strongly correlated with soil's native pH level (R^2 = 0.8, Fig. S23D), while parameter c (y-intercept) and a+c (asymptotic y value), scaling parameter (b) stayed mostly constant across soils with different pH levels. This indicates that the titration curve's general shape is similar for all soils, but the titration curve shifts horizontally depending on the soil's original pH level.

Soil's native pH, which determines the horizontal shift of the titration curves, was strongly correlated to the cation exchange capacity (CEC, milliequivalent charge / 100g) (R^2 = 0.88, Fig. S23D). This was expected because soils with higher CEC will have a greater number of negative charges in the clay particles and hence more likely to adhere to protons. This will result in fewer free protons in the soil pore water and, thus result in more basic pH levels. In the literature, CEC is reported to be determined by soil's clay particles and its organic matter, because CEC is proportional to how much negative charge the soil has on the aggregate's surface. However, in our dataset, the percent clay and organic matter did not correlate strongly with CEC (Fig. S23D, see Fig. S23E for percent clay). CEC appeared to be determined by Ca^{2+} ion concentration in the soil and not by other cations (Mg^{2+}, K^{+}, Na^{+}). Soil pH was inversely correlated with S, P, Al, and Fe concentrations, which can either be the cause or result of the soil's pH (Fig. S23D). In sum, we can attribute the horizontal shift of the pH titration curves to their varying native soil pH levels, which is potentially determined by the CEC and the Ca^{2+} ion concentrations (see summarized diagram in Fig. S23C).

Evidence for long-term pH adaptation from phyla's differential response to pH perturbations

We observed that long-term pH variation (different native soil pH) shifts the pH boundaries between functional regimes (Fig. 6). To see if those shifts of pH boundary can be explained by taxa's differential response to perturbed pH due to long-term pH adaptation, we further asked whether the pH values where the abundances of taxa (Firmicutes, Bacteriodota, and Proteobacteria) exhibit large changes also agree with the boundaries between regimes determined solely by nitrate utilization dynamics (Fig. 3). We observed the growth folds of taxa for the transition from Regime II to Regime III and the survival folds of taxa for the transition from Regime II to Regime I (Fig. S21A). Growth

bioRxiv preprint doi: https://doi.org/10.1101/2024.03.15.584851; this version posted May 28, 2024. The copyright holder for this preprint (which was not certified by peer review) is the author/funder. This article is a US Government work. It is not subject to copyright under 17 USC 105 and is also made available for use under a CC0 license.

folds were computed by endpoint absolute abundance ratio of $\mathrm{Abs}_{CHL-}/Abs_{CHL+}$ (chloramphenicol untreated/treated conditions) and survival folds were computed by absolute abundance ratio of Abs_{CHL+}/Abs_{T_0}, representing taxa's endpoint absolute abundance in CHL+ conditions compared to the initial time point (T_0) for each perturbed pH level.

To understand the transition to the Acidic death regime (Regime I), we observed the survival folds of Proteobacteria and Bacteroidota phyla across perturbed pH levels. Then, we set an identical survival fold threshold for all soils (red lines in Fig. S21) to compute the pH at which the survival fold goes below that threshold during acidic perturbation. We used two distinct definitions to choose a threshold for the survival fold. The first was a definition of "dying" where the taxa's abundance started to decline in abundance compared to T_0 (survival fold threshold < 1, red solid lines in Fig. S21A). The second was a definition of "dead" where the taxa's abundance was close to 0 (survival fold threshold $\rightarrow 0$, red dashed lines in Fig. S21A). For each of these definitions, the pH transition points were plotted (Fig. S21B with the first "dying" definition and Fig. S21C with the second "dead" definition) and compared to the trends of functional regime boundaries (transition from Regime II to I). Employing the 'dying' definition with Proteobacteria, Bacteroidota (Fig. S21B) allowed us to recapitulate the phenomenon observed in the functional data, where the fitted slope of Boundary I-II was less than 1, as shown in Fig. 6A. This suggests that these phyla in the relatively neutral soil are more tolerant of larger ΔpH change until they start to die than those in acidic soils, possibly due to variations in soil titration curves (Fig. 6B). Because the fitted slope is greater than 0, this also means that these phyla in relatively acidic soils can tolerate lower acidic pH conditions than those in neutral soils, which signals long-term pH adaptation. The 'dead' definition threshold resulted in a flat slope close to 0. This suggests that, despite long-term adaptation to varying native soil pH levels, these taxa have similar pH thresholds at which complete decimation occurs.

Similarly, to understand the transition to the Resurgent growth regime (Regime III), we observed the growth folds of Firmicutes phylum across perturbed pH levels. Then, we applied an

bioRxiv preprint doi: https://doi.org/10.1101/2024.03.15.584851; this version posted May 28, 2024. The copyright holder for this preprint (which was not certified by peer review) is the author/funder. This article is a US Government work. It is not subject to copyright under 17 USC 105 and is also made available for use under a CC0 license.

identical growth fold threshold for all soils (red lines in Fig. S21A) to compute the pH at which the growth fold goes above the threshold during basic perturbations. These pH transition points were plotted (Fig. S21B&C) and compared to the trends of functional regime boundaries (transition from Regime II to III). Consistent with the trend of functional regime boundary II-III (Fig. 6A), the abundance of Firmicutes began to increase at higher pH values as the native soil pH increased. Since the NaOH amount, and consequently, the carbon nutrient level, remains constant at the pH boundary of II-III (Fig. S24), the reason Firmicutes increases at higher pH values is not linked to the amount of nutrients available. Therefore, this is another signal for taxa adaptation to long-term pH variation.

Supplementary Tables

bioRxiv preprint doi: https://doi.org/10.1101/2024.03.15.584851; this version posted May 28, 2024. The copyright holder for this preprint (which was not certified by peer review) is the author/funder. This article is a US Government work. It is not subject to copyright under 17 USC 105 and is also made available for use under a CC0 license.

Table S1: Varying experimental conditions in previous studies

Paper	Soil/source	Carbon	Chloramphenicol	Water content	Measurement (time)
Wijler & Delwiche (1954)	1 soil's pH modified by H2SO4 or KOH	Dried alfalfa added	Untreated	Added water and dried	N2O/N2 gas (17 days)
Nömmik (1956)	6 soils (pH 5.2-7.6), each pH modified by HCl or KOH	Untreated, wheat straw, glucose conditions	Untreated	100% water saturation	N2O/N2 gas, NO3-, NO2-, NH4+ (12 days)
Bremner & Shaw (1958)	8 soils (pH 3.6-8.6) and 2 of those soils(pH 3.6-4.1)'s pH modified by CaOH	Glucose	Untreated	Waterlogged (slurry)	Loss of N (30 days)
Valera & Alexander (1961)	5 pure cultures of denitrifiers tested in buffered media of different pH levels	Glucose and yeast extract	Untreated	Defined buffered medium	N2 gas
Burford & Bremner (1975)	17 soils (pH 5.8-7.8)	Untreated	Untreated	Waterlogged (slurry)	Nitrous oxdie/NO/N2 gas, NO3-, NO2-, NH4+ (7 days)
Van Cleemput et al. (1975)	1 soil's pH modified by HCl or NaOH	Dried ground plant material	Untreated	Waterlogged (slurry)	N2O/N2 gas, NO3-, NO2- (100 hrs)
Smith et al. (1978)	4 soils (pH 6.4-7.6)	Untreated	Chloramphenicol and untreated	Waterlogged (slurry)	N2O while acetylene inhibition (10 hrs)
Smith & Tiedje (1979)	3 soils and pure cultures of denitrifiers	Succinate or glucose added and untreated	Chloramphenicol and untreated	Waterlogged (slurry)	N2O while acetylene inhibition (10 hrs)
Koskinen & Keeney (1982)	4 soils (pH 4.6-6.9)	Untreated	Untreated	20% water content	N2O/NO/N2 gas, NO3-, NO2-, NH4+ (16 days)
Waring & Gilliam (1983)	15 acidic soils (pH 3.42-5.54) and 5 of those soils limed to increase pH	Glucose and untreated	Untreated	Waterlogged (slurry)	NO3-, NO2- (20 days)

bioRxiv preprint doi: https://doi.org/10.1101/2024.03.15.584851; this version posted May 28, 2024. The copyright holder for this preprint (which was not certified by peer review) is the author/funder. This article is a US Government work. It is not subject to copyright under 17 USC 105 and is also made available for use under a CC0 license.

Paper	Soil/source	Carbon	Chloramphenicol	Water content	Measurement (time)
Parkin et al. (1985)	2 soils (pH 4, 6), each pH modified by H2SO4 or NaOH	Glucose for short-term (DEA), untreated for long-term (DP)	Chloramphenicol (DEA) and untreated (DP)	Waterlogged (slurry) for DEA	N2O while acetylene inhibition. Denitrifying enzyme activity (DEA) (2 hrs), Denitrification potential (DP) (5 days)
Nägele & Conrad (1990)	3 soils (pH 4-7.8), each pH modified by HCl or NaOH	Glucose	Chloramphenicol and untreated	Waterlogged (slurry)	N2O/NO/N2 gas, NO3-, NO2-, NH4+ (20 hrs)
Drury et al. (1991)	13 soils (pH 4.88-7.79)	Glucose for denitrification potential, untreated for background denitrification rate	Untreated	Field capacity	N2O while acetylene inhibition (75 hrs)
Bandibas et al. (1994)	18 soils (pH 4-7.8)	Untreated	Untreated	Field capacity, saturation, waterlogged	N2O/N2 gas, NO3-, NO2-, NH4+ (20 days)
Yamulki et al. (1997)	3 soils (pH 3.9, 5.9, 7.6) and the acid soil(pH 3.9)'s pH modified by NaOH	Untreated	Untreated	Unadjusted	N2O/NO/N2 gas, NO3-, NO2- (6hrs), field measurements (12 months)
Ellis et al. (1998)	4 soils (pH 3.3-6.1)	Untreated	Chloramphenicol and untreated	Waterlogged (slurry) for anaerobic condition and aerobic condition	N2O with and without acetylene inhibition, NO3-, NO2-, NH4+ (48 hrs)
Šimek et al. (2000)	13 soils (pH 5.62-7.77)	Glucose	Chloramphenicol (DEA) and untreated (DP)	Waterlogged (slurry)	N2O while acetylene inhibition. Denitrifying enzyme activity (DEA) (30-60 min), Denitrification potential (DP) (48 hrs)

bioRxiv preprint doi: https://doi.org/10.1101/2024.03.15.584851; this version posted May 28, 2024. The copyright holder for this preprint (which was not certified by peer review) is the author/funder. This article is a US Government work. It is not subject to copyright under 17 USC 105 and is also made available for use under a CC0 license.

Paper	Soil/source	Carbon	Chloramphenicol	Water content	Measurement (time)
Šimek et al. (2002)	5 soils (pH 4.9-7.9), each pH modified by H2SO4 or NaOH	Glucose	Chloramphenicol (DEA) and untreated (DP)	Waterlogged (slurry)	N2O while acetylene inhibition. Denitrifying enzyme activity (DEA) (30 min-3 hrs), Denitrification potential (DP) (48 hrs)
Liu et al. (2010)	3 soils (pH 3-4), each pH modified by long-term liming	Glutamate and untreated	Untreated	Unadjusted (1st run), flooding and draining (2nd run)	N2O/N2 gas (21 hrs)
Cuhel et al. (2010)	3 soils (pH 5.5-7.7), each pH modified by10-month application of H2SO4 and KOH	Glucose	Chloramphenicol for short-term (DEA), untreated for in-situ field chamber measurements	Waterlogged (slurry) for DEA	N2O/N2 gas, NO3-, NO2-, NH4+. In-situ field chamber measurements (74 hrs), Denitrifying enzyme activity (DEA) (30-60 min)
Bergaust et al. (2010)	1 pure culture in 5 pH levels (pH 6.0-7.5)	Succinate	Untreated	Defined buffered medium	N2O/NO/N2 gas, NO3-, NO2- (72 hrs)
Dörsch et al. (2012)	Cells extracted from 3 soils (pH 5.4-7.1) perturbed to pH 5.4 and 7.1	Glutamate	Untreated	Defined buffered medium	N2O/NO/N2 gas, NO3-, NO2- (130 hrs)
Samad et al. (2016)	13 soils (pH 5.57-7.03)	Untreated	Untreated	Flooded and drained	N2O/NO/N2 gas (200 hrs)

bioRxiv preprint doi: https://doi.org/10.1101/2024.03.15.584851; this version posted May 28, 2024. The copyright holder for this preprint (which was not certified by peer review) is the author/funder. This article is a US Government work. It is not subject to copyright under 17 USC 105 and is also made available for use under a CC0 license.

Table S2: Relevant conclusions from previous studies

Paper	Conclusions relevant to this paper
Wijler & Delwiche (1954) [85]	Total denitrification rates were quite constant above pH 6.
Nömmik (1956) [63]	Total denitrification rates are low below pH 5 and increased until 7-8. Further increase in pH suppressed denitrification. Different soils showed similar trends.
Bremner & Shaw (1958) [59]	When compared with soils without pH modification, soil with native pH of 7.5 had a higher denitrification rate. When pH was increased in soils with acidic pH levels, the denitrification rate increased. Denitrification potential correlated very well with mineralizable carbon. Easily decomposable substances (glucose, mannitol, and sucrose) have greater effects on increasing denitrification rate than difficultly decomposable materials (lignin and sawdust).
Valera & Alexander (1961) [86]	Pure cultures exhibited the highest denitrification rate between pH 7-8.
Burford & Bremner (1975) [36]	Denitrification capacity was very highly correlated with water-soluble organic carbon and mineralizable carbon, and less but significantly correlated with total organic carbon in soils.
Van Cleemput et al. (1975) [11]	Soils in very acidic conditions also show substantial denitrification. Authors suggested pH might affect the availability of readily decomposable organic matter, which correlated with the denitrification rate.
Smith et al. (1978) [55]	The denitrification rates increased from the 0hr-3hr period until a linear rate was attained. The linear rate corresponded to mineralizable carbon.
Smith & Tiedje (1979) [31]	Chloramphenicol does not affect the denitrifying rate in 0-3hrs, called linear Phase I, but prevented the rate from increasing during the transition to linear Phase II (4-8hrs) compared to the untreated samples. Even when glucose was added, the linear rate of Phase I did not change or just increased slightly, but it later prompted a logarithmic increase of denitrifying activity. Irrigating the soils before the incubation increased the linear rate of Phase I. Therefore, it can be concluded that Phase II is determined by a state where derepression of de novo enzyme synthesis is complete and therefore impacted by available electron donor, pH, and temperature, while Phase I rate is determined by indigenous enzyme activity.
Koskinen & Keeney (1982) [10]	Denitrification rate was correlated with mineralizable carbon but not consistently related to pH or total organic carbon. Therefore, the rate of organic C mineralization rather than pH controls the rate of denitrification in C-limited systems.
Waring & Gilliam (1983) [87]	Soluble organic carbon is more important in denitrification than pH. Significant denitrification can also happen in strongly acidic soils (below pH 4).

bioRxiv preprint doi: https://doi.org/10.1101/2024.03.15.584851; this version posted May 28, 2024. The copyright holder for this preprint (which was not certified by peer review) is the author/funder. This article is a US Government work. It is not subject to copyright under 17 USC 105 and is also made available for use under a CC0 license.

Paper	Conclusions relevant to this paper
Parkin et al. (1985) [33]	Both natural denitrification rates and denitrifying enzyme activity (DEA) were greater in neutral soil, but denitrification rate of similar magnitude occurred in acid soil, which can be due to long-term selection of acid-tolerant denitrifying populations. With DEA measurements (activity of existing enzymes), acid soil (native pH 4) had the highest denitrification rate at 3.9 and neutral soil (native pH 6) had an optimal pH at 6.3.
Nägele & Conrad (1990) [88]	Unlike Parkin et al. (1985), under chloramphenicol treatment, acid soil (native pH 4) had greater reduced nitrate when pH was adjusted to 7 compared to pH 4. On the other hand, neutral soil (native pH 7.8) had greater reduced nitrate when pH was 7 and decreased as pH was lowered.
Drury et al. (1991) [58]	For background denitrification rate (C, nitrate unamended), denitrification rates were highly correlated with biomass C, which was also highly correlated with available organic carbon. This shows that soil denitrification is limited by available organic carbon. Background and potential denitrification rates did not correlate with each other. pH nor other soil physicochemical properties did not correlate with either background and potential denitrification rate (other than the correlations stated above).
Bandibas et al. (1994) [89]	Saturated conditions produced the most N2O compared to field capacity and waterlogged moisture conditions. Soil pH was the soil characteristic that best predicted N2O emission.
Yamulki et al. (1997) [32]	Field measurements did not show a clear effect of pH on N2O emissions. The highest N2O emission rate was observed after a period of relatively high rainfall in the pH 5.9 plot. Average N2O emission rates were higher in the pH 7.6 and 5.9 plots than in the pH 3.9 plot. In lab measurements. When the soil's pH was increased in the lab from pH 3.9 to above 6, NO2 emission dropped 40%.
Ellis et al. (1998) [56]	In anaerobic conditions, denitrification rates increased with higher pH levels both in CHL+ and CHL- conditions, which is contrary to Parkin et al. (1985) probably due to longer (48hr) time scale. Chloramphenicol reduced the denitrification rate in all soils with different pH levels. However, the CO2 production was not significantly reduced in anaerobic conditions. Increasing pH increased the CO_2 production rate in both CHL+ and CHL- conditions.
Šimek et al. (2000) [60]	No significant relationship was found between short-term denitrifying enzyme activity(DEA) and longer-term denitrification potential(DP), indicating the independence of the existing metabolic activity of the denitrifier community (DEA) and its potential for rapid development when the environmental conditions change (DP). Existing enzyme activity did not correlate with pH. However, denitrification potential positively correlated with pH, available organic carbon, and biomass.

bioRxiv preprint doi: https://doi.org/10.1101/2024.03.15.584851; this version posted May 28, 2024. The copyright holder for this preprint (which was not certified by peer review) is the author/funder. This article is a US Government work. It is not subject to copyright under 17 USC 105 and is also made available for use under a CC0 license.

Paper	Conclusions relevant to this paper
Šimek et al. (2002) [17]	In agreement with Parkin et al. 1985, for existing denitrifying enzymes, the denitrification rate was highest close to their native pH. Optimal pH shifted to neutral pH (pH 6 - 8) as denitrification rate was measured in longer periods. It is proposed that this is either due to the development of a community of denitrifiers that can grow better at the neutral pH or due to the accommodation of the existing populations to new conditions.
Liu et al. (2010) [90]	Soil pH has little effect on denitrification rate apart from the low denitrification rates in the very acid peat soil (pH 4.0). For acidic soil, neither the gene pools (nirS vs. nosZ) nor their transcription rates could explain the observed effects of low pH on N2O reductase activity, which implies that this low N2O reductase activity is due to post-transcriptional level, either by interfering with translation, protein assembly or by directly affecting the activity of the functional enzyme. Denitrification in unamended soil appeared to be based on the activation of a pre-existing denitrification proteome, because constant rates of N2 and N2O production were observed, and the transcription of functional genes was below the detection level, whereas glutamate-amended soils showed sharp peaks in the transcripts of nirS and nosZ, increasing the denitrification rates.
Cuhel et al. (2010) [18]	Denitrification rates were higher when soil's pH was perturbed to higher pH and were lower when perturbed to lower pH. Microbial biomass C followed a similar pattern.
Bergaust et al. (2010) [7]	Pure culture experiment of Paracoccus denitrificans revealed that the denitrification rate was highest at pH 7 and was slower at pH 6. This can be explained by the change in transcription of genes, where the maximum numbers of norB and nosZ transcripts were higher at pH 7 than at pH 6 and the maximum number of nirS transcripts was higher at pH 7 than at pH 6. However, as in Liu et al. 2010, transcription cannot explain the low N2O reduction (N2OR) rate at pH 6. They rule out the possibility of N2OR enzyme activity directly inhibited by low pH, because functional N2O reductase activity was only marginally affected by lowering pH to 6. Therefore, the loss of N2OR activity is due to unsuccessful assemblage/folding of the protein due to low pH in the periplasm.
Dorsch et al. (2012) [91]	Denitrification rates in extracted communities are higher in neutral pH 7.1 than in pH 5.4.
Samad et al. (2016) [61]	The denitrification rate (NO+N2O+N2 μmol N/h/vial) was higher in more neutral soils. Emission kinetics across all soils revealed that the denitrification rates under anoxic conditions were significantly associated with C mineralization (CO2 μmol/h/vial).

bioRxiv preprint doi: https://doi.org/10.1101/2024.03.15.584851; this version posted May 28, 2024. The copyright holder for this preprint (which was not certified by peer review) is the author/funder. This article is a US Government work. It is not subject to copyright under 17 USC 105 and is also made available for use under a CC0 license.

Table S3: Physical and chemical characteristics of soil samples

Soil No.	Soil ID	pH_{H2O}	Latitude	Longitude	Sand (%)	Silt (%)	Clay (%)	Total N (%)	Total C (%)	C:N ratio	Depth	Date
Soil1	Acidic4	4.703	46.785703	-117.079245	0.00	58.15	41.85	0.13	1.61	12.79	0-10cm	9/10/22
Soil2	Acidic12	5.094	46.781117	-117.080513	0.00	63.80	36.20	0.15	1.97	13.19	0-10cm	9/12/22
Soil3	CE239	4.987	46.781153	-117.080455	0.00	61.20	38.80	0.11	1.35	12.27	10-20cm	9/8/22
Soil4	SE56b	5.277	46.778744	-117.082738	0.00	57.40	42.60	0.10	1.26	13.31	10-20cm	9/8/22
Soil5	CE201	5.324	46.781049	-117.086242	0.00	58.70	41.30	0.12	1.57	13.07	10-20cm	9/8/22
Soil6	CE73	5.405	46.779637	-117.086172	0.00	61.20	38.80	0.13	1.65	12.97	10-20cm	9/8/22
Soil7	CE153	5.514	46.7805	-117.085431	0.00	58.70	41.30	0.13	1.76	13.82	10-20cm	9/8/22
Soil8	CE56a	5.552	46.778855	-117.082968	0.00	58.70	41.30	0.10	1.35	13.64	10-20cm	9/8/22
Soil9	CE277	5.822	46.781883	-117.0835	0.00	61.20	38.80	0.09	1.19	12.65	10-20cm	9/8/22
Soil10	CE253	5.975	46.781534	-117.084192	0.00	62.50	37.50	0.08	0.98	12.00	10-20cm	9/8/22
Soil11	CE234	6.186	46.781301	-117.082533	0.00	62.50	37.50	0.13	1.88	14.04	10-20cm	9/8/22
Soil12	CE229	6.255	46.781206	-117.084623	0.00	58.80	41.20	0.09	1.20	12.73	10-20cm	9/8/22
Soil13	Neutral7	6.435	46.781523	-117.084533	0.00	60.00	40.00	0.08	0.99	12.06	10-20cm	9/11/22
Soil14	Neutral2	6.545	46.781308	-117.084696	0.00	58.80	41.20	0.11	1.28	12.06	10-20cm	9/11/22
Soil15	Neutral5	6.789	46.781416	-117.084818	0.00	63.70	36.30	0.10	1.31	13.79	10-20cm	9/11/22
Soil16	Neutral6	6.860	46.781524	-117.084694	0.00	61.20	38.80	0.09	1.07	12.30	10-20cm	9/11/22
Soil17	Neutral3	7.052	46.781194	-117.084732	0.00	60.00	40.00	0.09	1.09	12.56	10-20cm	9/11/22
Soil18	Neutral1	7.681	46.781354	-117.084812	0.00	63.70	36.30	0.10	1.21	12.37	10-20cm	9/11/22
Soil19	Neutral4	8.232	46.781222	-117.084882	0.00	62.50	37.50	0.08	1.05	13.78	10-20cm	9/11/22
Soil20	CE251	8.323	46.781492	-117.085028	0.00	67.50	32.50	0.09	1.47	16.14	10-20cm	9/8/22

bioRxiv preprint doi: https://doi.org/10.1101/2024.03.15.584851; this version posted May 28, 2024. The copyright holder for this preprint (which was not certified by peer review) is the author/funder. This article is a US Government work. It is not subject to copyright under 17 USC 105 and is also made available for use under a CC0 license.

Supplementary Figures

bioRxiv preprint doi: https://doi.org/10.1101/2024.03.15.584851; this version posted May 28, 2024. The copyright holder for this preprint (which was not certified by peer review) is the author/funder. This article is a US Government work. It is not subject to copyright under 17 USC 105 and is also made available for use under a CC0 license.

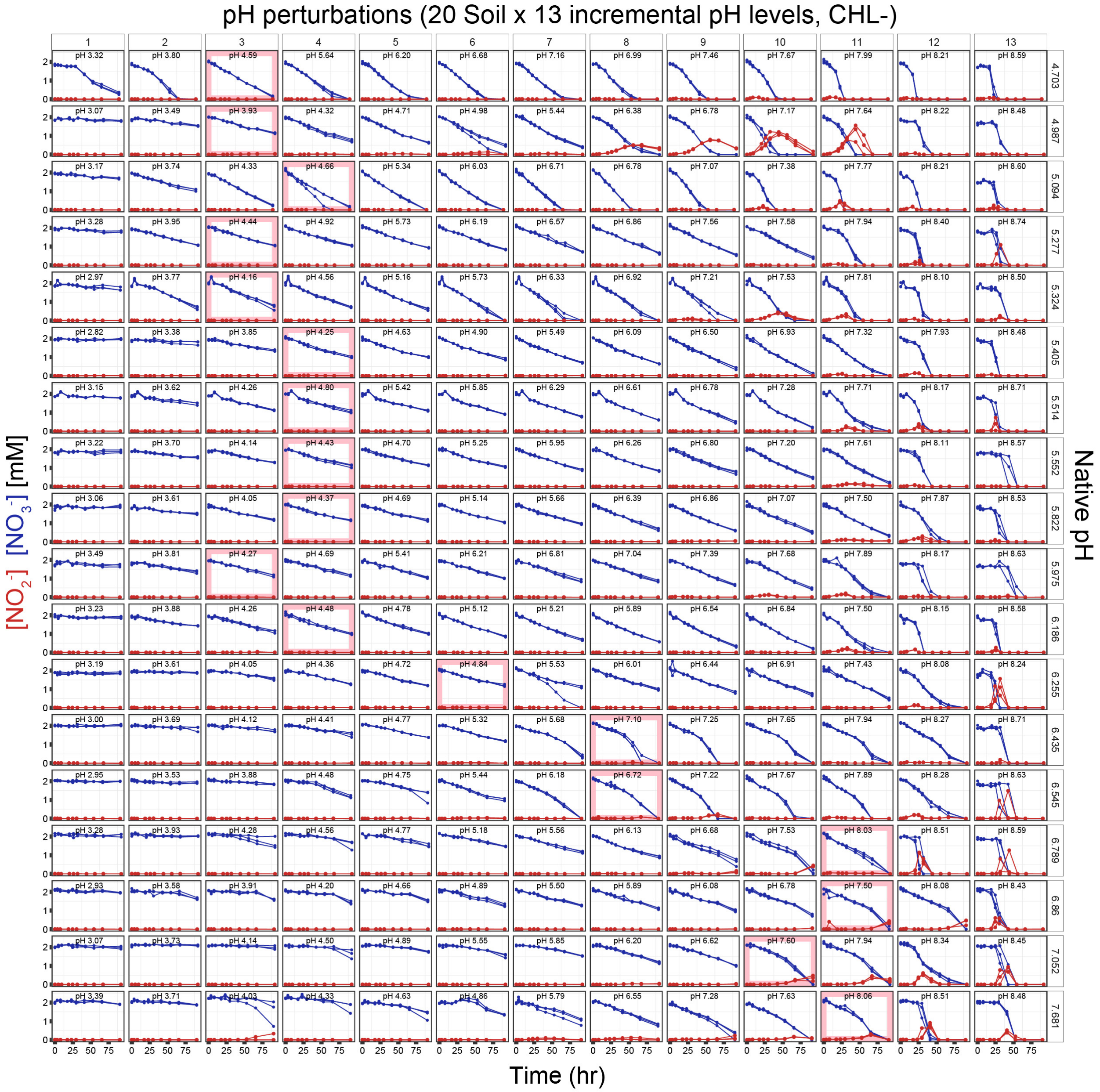

Figure S1: **Flux dynamics of nitrate and nitrite of the dataset.** Time series measurements of nitrate (blue points) and nitrite (red points) across 4 days are shown. (Continued)

bioRxiv preprint doi: https://doi.org/10.1101/2024.03.15.584851; this version posted May 28, 2024. The copyright holder for this preprint (which was not certified by peer review) is the author/funder. This article is a US Government work. It is not subject to copyright under 17 USC 105 and is also made available for use under a CC0 license.

Figure S1: (Continued from the previous page) Each row is from the identical soil sample of a native pH level (pH$_{H_2O}$), indicated at the right end of each row in the order of most acidic (top) to most basic (bottom). Each row has 13 columns which are the 13 different levels of short-term pH perturbations. The targeted perturbed pH levels were determined by constructing a soil pH titration curve before the experiment and computing how much acid (HCl) or base (NaOH) to add to the slurries. Perturbed pH levels are indicated inside each panel, which are obtained by measuring the stabilized pH values at the endpoint of the experiment (see Methods). Each line connects the point of measurements of a replicate, constituting the 3 replicates per perturbed condition. The pink-colored box for each row indicates the condition without any acid/base addition, where the pH of these conditions also changes with incubation. Soil 19 and Soil 20 are not shown due to having different numbers of perturbed pH levels (7 and 3, respectively).

bioRxiv preprint doi: https://doi.org/10.1101/2024.03.15.584851; this version posted May 28, 2024. The copyright holder for this preprint (which was not certified by peer review) is the author/funder. This article is a US Government work. It is not subject to copyright under 17 USC 105 and is also made available for use under a CC0 license.

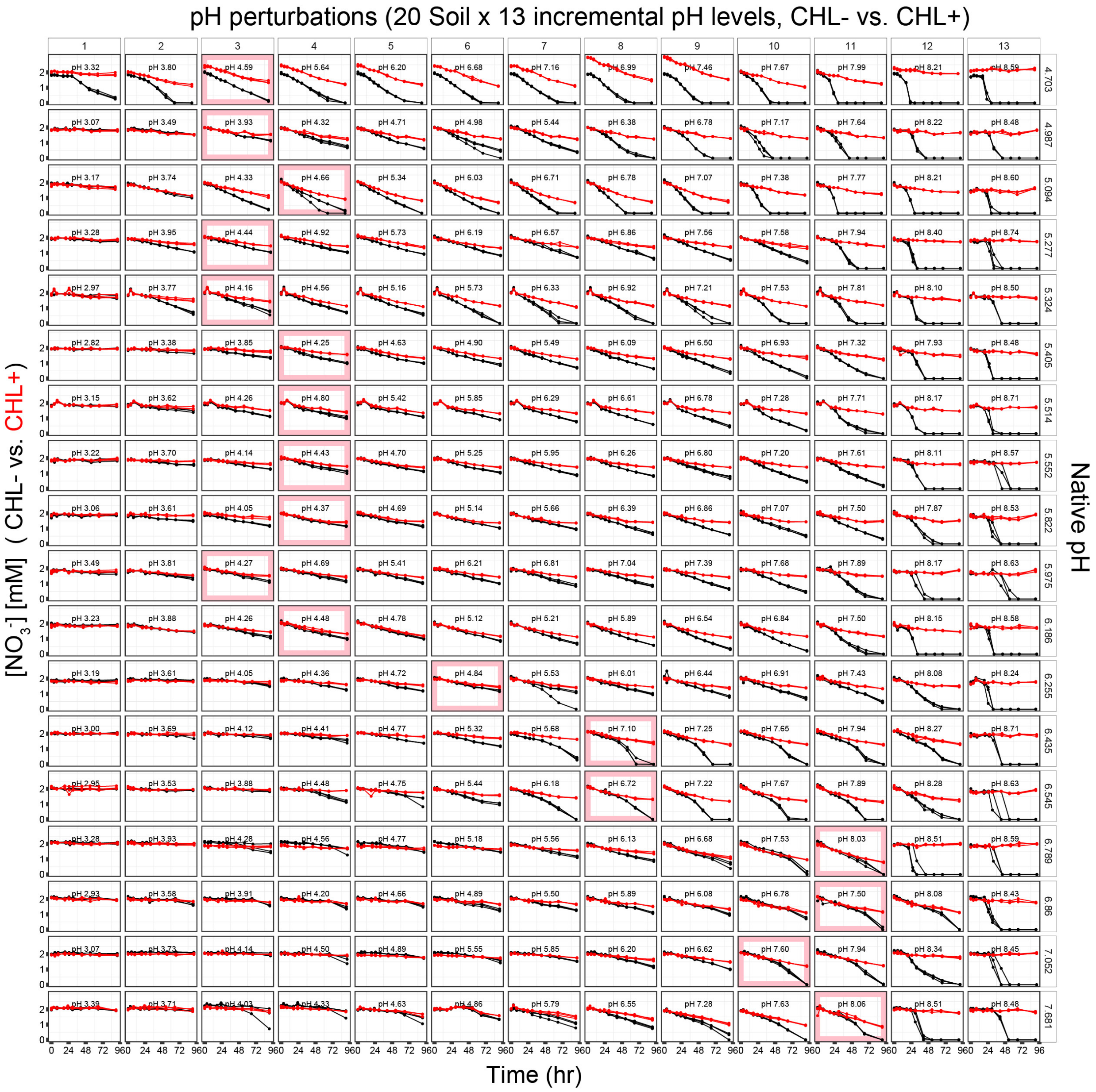

Figure S2: **Nitrate dynamics of chloramphenicol untreated (CHL-) and treated (CHL+) conditions in the dataset.** Time series measurements of nitrate in chloramphenicol-untreated (CHL-, black points) and treated (CHL+, red points) across 4 days are shown. (Continued)

bioRxiv preprint doi: https://doi.org/10.1101/2024.03.15.584851; this version posted May 28, 2024. The copyright holder for this preprint (which was not certified by peer review) is the author/funder. This article is a US Government work. It is not subject to copyright under 17 USC 105 and is also made available for use under a CC0 license.

Figure S2: (Continued from the previous page) Each row is from the identical soil sample of a native pH level (pH$_{H_2O}$), indicated at the right end of each row in the order of most acidic (top) to most basic (bottom). Each row has 13 columns which are the 13 different levels of short-term pH perturbations. The targeted perturbed pH levels were determined by constructing a soil pH titration curve before the experiment and computing how much acid (HCl) or base (NaOH) to add to the slurries. Perturbed pH levels are indicated inside each panel, which are obtained by measuring the stabilized pH values at the endpoint of the experiment (see Methods). Each line connects the point of measurements of a replicate, constituting the 3 replicates per perturbed condition. The pink-colored box for each row indicates the condition without any acid/base addition, where the pH of these conditions also changes with incubation. Soil19 and Soil20 are not shown due to having different numbers of perturbed pH levels (7 and 3, respectively).

bioRxiv preprint doi: https://doi.org/10.1101/2024.03.15.584851; this version posted May 28, 2024. The copyright holder for this preprint (which was not certified by peer review) is the author/funder. This article is a US Government work. It is not subject to copyright under 17 USC 105 and is also made available for use under a CC0 license.

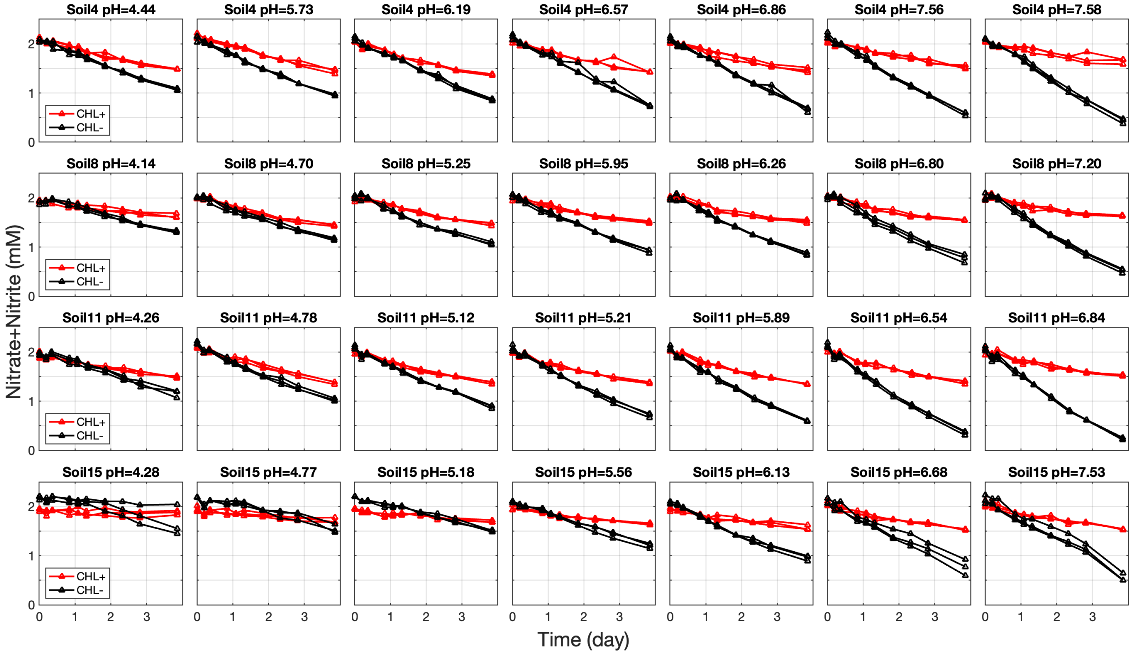

Figure S3: **Nitrate + nitrite concentration dynamics to show constant nitrite reduction rates.** The points indicate the time-series measurement of the sum of nitrate and nitrite concentrations. Concentrations from chloramphenicol-treated (CHL+) samples are in red and untreated (CHL-) samples are in black, with lines connecting each of the three biological replicates. A subset of pH perturbed conditions (each row is from the same native pH soil, with varying perturbed pH levels) is shown. Nitrate + nitrite dynamics (in CHL- conditions) are linear, indicating that the community's nitrite consumption is constant across time.

bioRxiv preprint doi: https://doi.org/10.1101/2024.03.15.584851; this version posted May 28, 2024. The copyright holder for this preprint (which was not certified by peer review) is the author/funder. This article is a US Government work. It is not subject to copyright under 17 USC 105 and is also made available for use under a CC0 license.

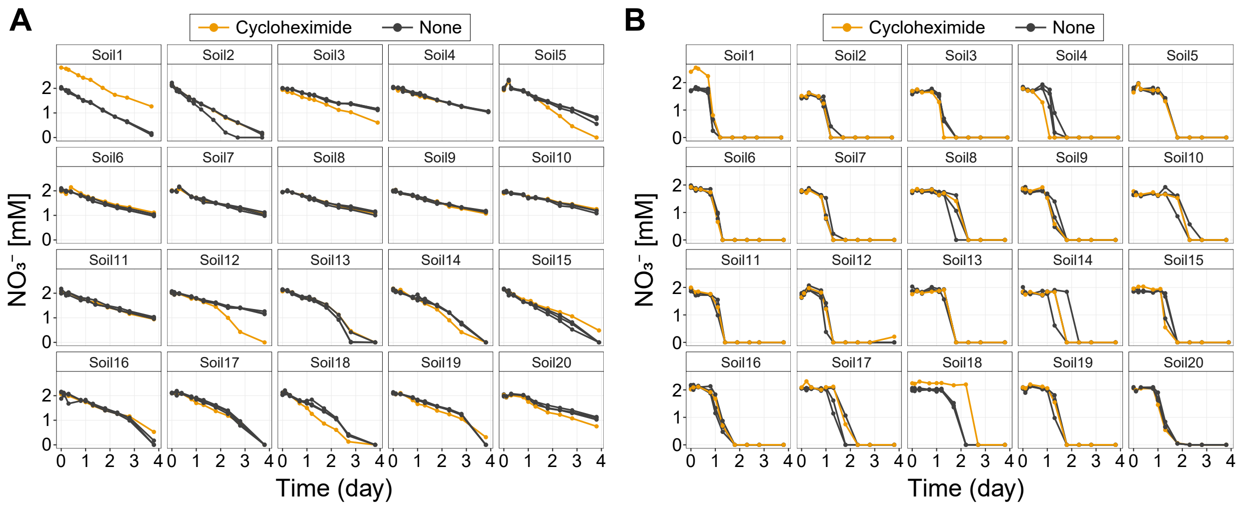

Figure S4: **Cycloheximide antifungal controls suggest a minimal role for fungi in nitrate utilization dynamics.** Nitrate dynamics across a 4-day anaerobic incubation with and without cycloheximide treatment for all 20 soils. Panel **(A)** shows the nitrate dynamics of pH-unperturbed samples with (orange data points, 1 replicate) and without cycloheximide (black data points, 3 biological replicates) treatment, while **(B)** illustrates the nitrate dynamics for basic-perturbed samples, also with (orange, 1 replicate) and without cycloheximide (black, 3 biological replicates) treatment. Most of the dynamics were not affected by the application of 200 ppm cycloheximide. Only 1 case out of 40 samples (Soil 18 in **B**) showed delayed nitrate reduction when the antifungal was treated. This means that fungi do not play a significant role during nitrate reduction.

bioRxiv preprint doi: https://doi.org/10.1101/2024.03.15.584851; this version posted May 28, 2024. The copyright holder for this preprint (which was not certified by peer review) is the author/funder. This article is a US Government work. It is not subject to copyright under 17 USC 105 and is also made available for use under a CC0 license.

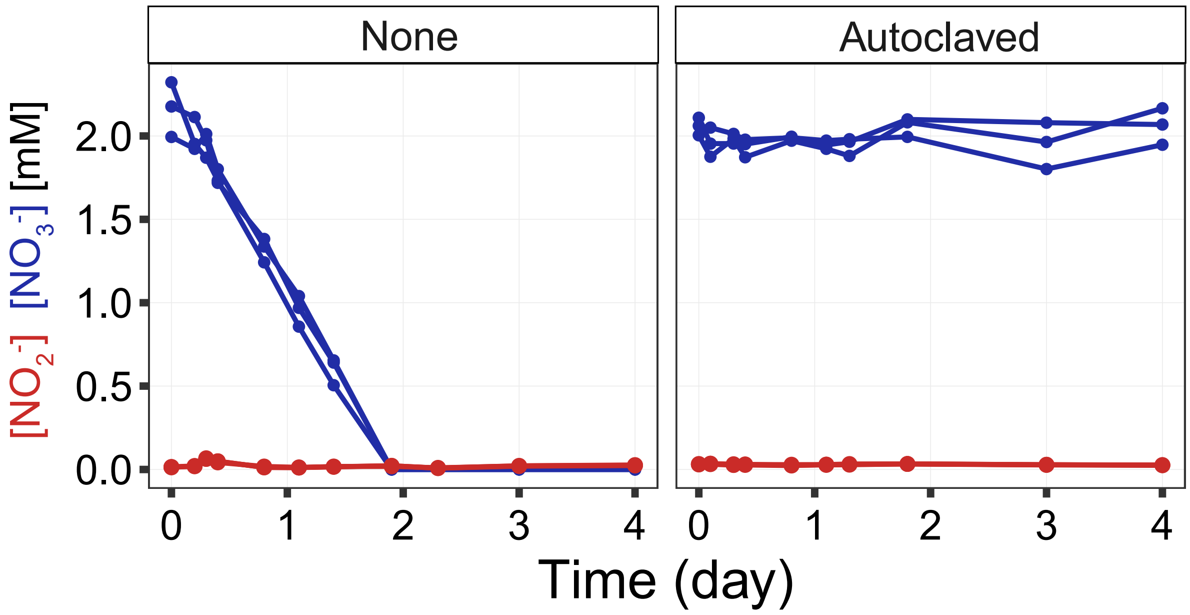

Figure S5: **The results from autoclaving soil suggest the absence of abiotic (chemical) nitrate reduction.** Nitrate (blue data points) and nitrite (red data points) dynamics of a soil sample with (right, Autoclaved) and without (left, None) autoclaving procedure. The autoclaving was performed at 120 °C for 99 minutes and repeated five times at two-day intervals. The soil used in this experiment was collected from LaBagh Woods (latitude 41.977855, longitude -87.742585), Sauganash Prairie, Chicago, IL, USA, on January 18, 2022. Contrary to the soil without the sterilization, nitrate reduction did not occur in the soil with the sterilization process (autoclaving). This rules out the possibility of abiotic (chemical) nitrate reduction occurring in soils.

bioRxiv preprint doi: https://doi.org/10.1101/2024.03.15.584851; this version posted May 28, 2024. The copyright holder for this preprint (which was not certified by peer review) is the author/funder. This article is a US Government work. It is not subject to copyright under 17 USC 105 and is also made available for use under a CC0 license.

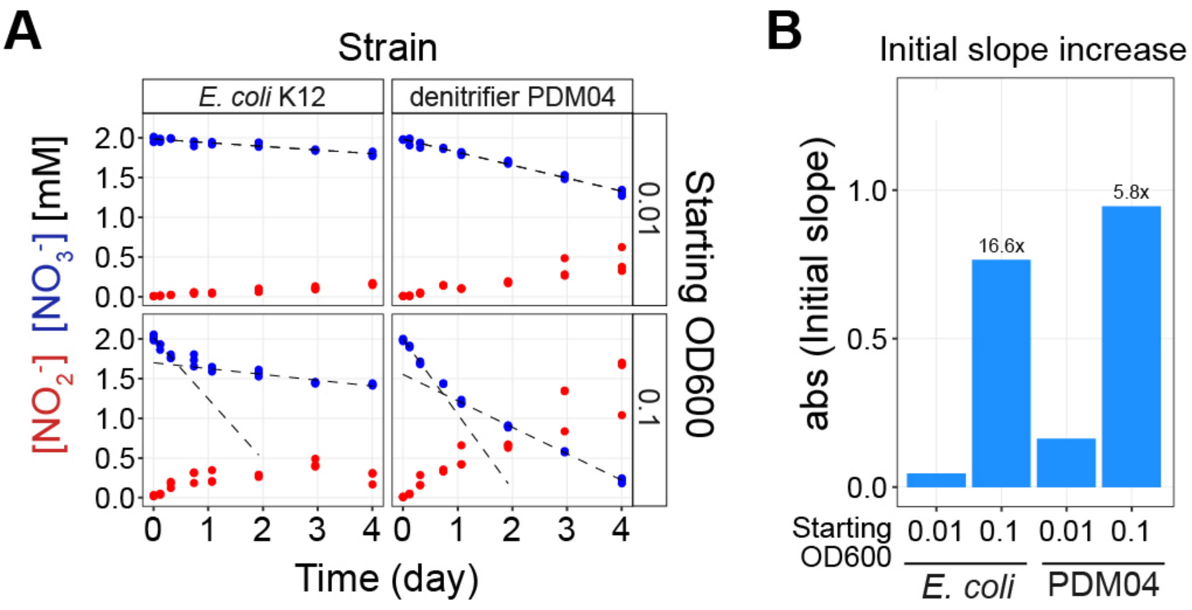

Figure S6: **Linear metabolite dynamics recapitulated from monoculture experiments in the absence of external carbon sources (A)** Nitrate and nitrite dynamics of monoculture experiments using *E. coli* K12 and the denitrifier *Pseudomonas sp.* PDM04 strains over 4 days with no external carbon provided in the culture media (see SM for detailed experimental methods). The top two panels have a starting OD600 (optical density at 600nm) of 0.01 and the bottom panels have a starting OD600 (optical density at 600nm) of 0.1. The x-axis represents time in days, and the y-axis represents the concentration of nitrate (blue points) or nitrite (red points) reduced (mM), each condition having three biological replicates. The linear dynamics demonstrate that nitrate reduction can occur even in the absence of external carbon, resolving the previous contradiction about the necessity of carbon for this process. The dashed lines represent linear regression of the dynamics: for the top panels, linear regression used all data points, while in the bottom panels, initial slopes were derived from fitting the first three points, and late slopes were calculated using the last four points. **(B)** The fitted initial slope values in nitrate reduction (using initial slopes in **A**). The different bars indicate the initial slopes from different conditions of starting OD600 values (0.01 and 0.1) and two strains. The plot underscores the effect of starting biomass on initial nitrate reduction rates, with factor increase of initial slopes annotated on top of the bars.

bioRxiv preprint doi: https://doi.org/10.1101/2024.03.15.584851; this version posted May 28, 2024. The copyright holder for this preprint (which was not certified by peer review) is the author/funder. This article is a US Government work. It is not subject to copyright under 17 USC 105 and is also made available for use under a CC0 license.

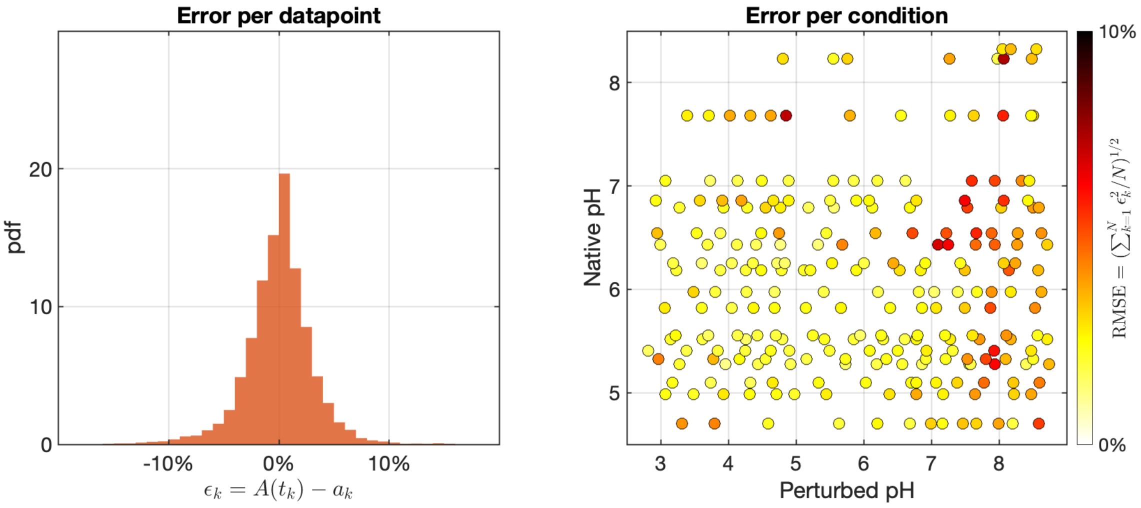

Figure S7: **Quantification of error in model fitting** Error per data point (Left panel): The probability density function (pdf) represents the distribution of errors for individual data points of nitrate measurements at time point k. Errors are calculated as the difference between the model's predicted nitrate concentration $A(t_k)$ and the observed nitrate amounts a_k for either the chloramphenicol-untreated(CHL-) or treated(CHL+) conditions, normalized by dividing by the input nitrate concentration (2mM) to be expressed as a percentage. Error per condition (right panel): Each dot represents the error for a specific experimental condition (triplicates), with the native pH of the sample on the y-axis and the perturbed pH on the x-axis. The error per condition, indicated by the color of each point, is the square root of the mean-squared error (MSD) loss function minimized during parameter optimization of both CHL-/+ conditions of triplicates, normalized by the input nitrate concentration (2mM) to be expressed as a percentage (refer to Methods for the error computation).

bioRxiv preprint doi: https://doi.org/10.1101/2024.03.15.584851; this version posted May 28, 2024. The copyright holder for this preprint (which was not certified by peer review) is the author/funder. This article is a US Government work. It is not subject to copyright under 17 USC 105 and is also made available for use under a CC0 license.

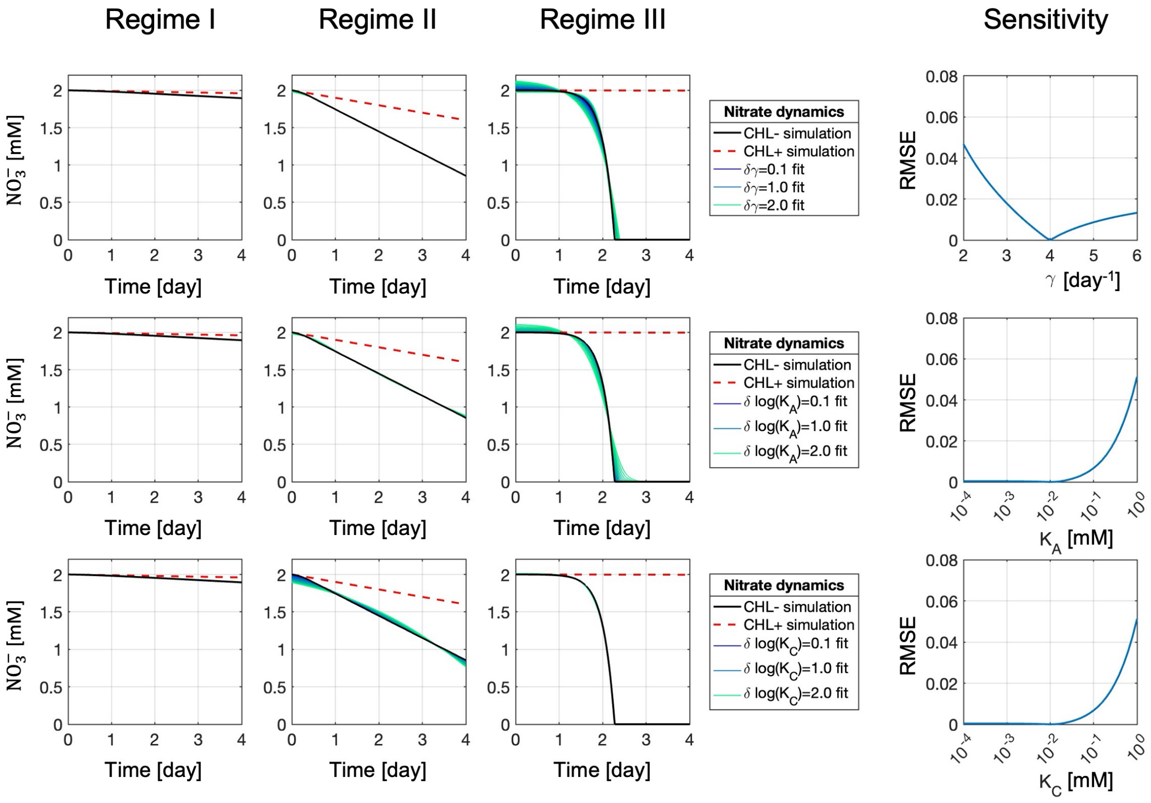

Figure S8: **Sensitivity analysis on model parameters γ, K_A, and $\tilde{K}_C$ to justify fixing these parameters** To justify the fixed parameters in the fitting scheme, we analyzed the sensitivity of γ, K_A, and $\tilde{K}_C$ by simulating dynamic data. To reflect the three typical dynamics (regimes) observed from the measurement, we simulated three nitrate curves by setting up the initial conditions to be $\tilde{x}(0) = 0.01, 0.1, 0.001 mM/day$ and $\tilde{C}(0) = 0.005, 0.05, 2mM$, respectively. Other parameters are given by $A_0 = A_0^c = 2mM$, $K_A = \tilde{K}_C = 0.01mM$, $\gamma = 4day^{-1}$. Black curves indicate the simulated nitrate dynamics from the chloramphenicol-untreated (CHL-) conditions, and red dashed lines indicate the simulated nitrate dynamics from the chloramphenicol-treated (CHL+) conditions. We then used different fixed values of parameters to fit the three examples. In the first row, we used different fixed γ values - from $\gamma = 2day^{-1}$ to $\gamma = 6day^{-1}$ - to fit three simulations. The square root of the mean-squared error (RMSE) is computed by the loss function (mean-squared difference of predicted and observed nitrate concentration for both CHL-/+ conditions) minimized during parameter optimization, normalized by the input nitrate concentration (2mM) to be expressed as a percentage (refer to Methods for loss function). We demonstrate very small mismatches (RMSE$< 5\%$) from these variations of parameter values, which are almost invisible in Regime I and Regime II fittings (purple lines indicate fitted results from $\gamma = 4 \pm 0.1\ day^{-1}$, blue lines indicate fitted results from $\gamma = 4 \pm 1\ day^{-1}$, green lines indicate fitted results from $\gamma = 4 \pm 2\ day^{-1}$). In the second and the third row, we used different fixed K_A and $\tilde{K}_C$ values - from $10^{-4}mM$ to $1mM$ - to fit three simulations. When $K_A < 0.1mM$ or $\tilde{K}_C < 0.1mM$, the mismatches were again very small (RMSE $< 1\%$) and invisible (purple lines indicate fitted results from $K_{A,C} = 10^{-2\pm0.1}mM$, blue lines indicate fitted results from $K_{A,C} = 10^{-2\pm1}mM$, and green lines indicate fitted results from $K_{A,C} = 10^{-2\pm2}mM$). These results indicate that the fixed values of γ, K_A and $\tilde{K}_C$ are insensitive in large ranges.

bioRxiv preprint doi: https://doi.org/10.1101/2024.03.15.584851; this version posted May 28, 2024. The copyright holder for this preprint (which was not certified by peer review) is the author/funder. This article is a US Government work. It is not subject to copyright under 17 USC 105 and is also made available for use under a CC0 license.

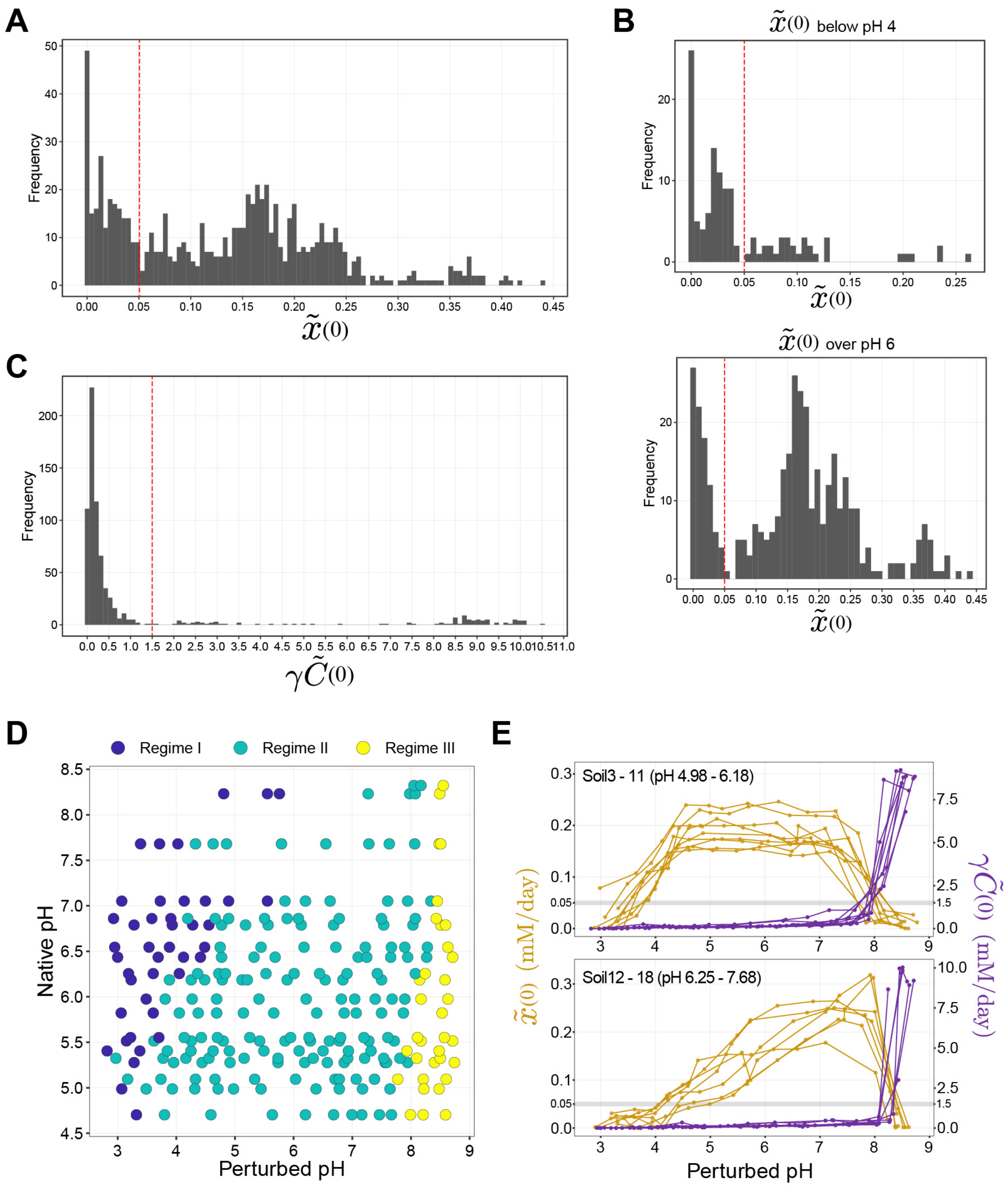

Figure S9: **Determining regime boundary thresholds with distributions of the parameters $\tilde{x}(0)$ and** $\gamma\tilde{C}(0)$ To determine the regime boundaries, we examined the distributions of parameters fitted to the functional data for $\tilde{x}(0)$ and $\gamma\tilde{C}(0)$. (Continued)

bioRxiv preprint doi: https://doi.org/10.1101/2024.03.15.584851; this version posted May 28, 2024. The copyright holder for this preprint (which was not certified by peer review) is the author/funder. This article is a US Government work. It is not subject to copyright under 17 USC 105 and is also made available for use under a CC0 license.

Figure S9: (Continued from the previous page) **(A)** $\tilde{x}(0)$ had a bimodal frequency distribution, having two peaks. **(B)** This bi-modality becomes more evident when we separately observe its distribution from the left half (perturbed pH < 4) and right half (perturbed pH > 6) of the parameter space displayed in the perturbed pH vs. native pH grid in Figure 3C. We set the threshold for the $\tilde{x}(0)$ boundary where these two modes are separated ($\tilde{x}(0)$ = 0.05). **(C)** $\gamma\tilde{C}(0)$ showed an uni-modal frequency distribution. We set the threshold ($\gamma\tilde{C}(0)$ = 1.5) at the tail of the distribution, where the $\gamma\tilde{C}(0)$ threshold also separated the Regime III samples in the top-left quadrant of the $\tilde{x}(0)$ vs. $\gamma\tilde{C}(0)$ scatter plot (Fig. 3A). The separation of Regime I and Regime II data points may not be clear cut in the $\tilde{x}(0)$ vs. $\gamma\tilde{C}(0)$ scatter plot (Fig. 3A). However, when we plot $\tilde{x}(0)$ of different soils (dark yellow colored lines) by grouping them into relatively acidic (Soil3–11 (pH 4.98-6.18), **(E)** top panel) and neutral soils (Soil12–18 (pH 6.25-7.68), **(E)** bottom panel), the transition from Regime II (large $\tilde{x}(0)$) to Regime I (small $\tilde{x}(0)$) is evident going towards more acidic pH perturbations, especially in the naturally acidic soils (top panel), because the large $\tilde{x}(0)$ levels are sustained over a wide pH range in Regime II. **(D)** With these thresholds of two parameters, we can define the three different regimes of adaptive behavior across native pH and perturbed conditions (colored differently by regimes).

bioRxiv preprint doi: https://doi.org/10.1101/2024.03.15.584851; this version posted May 28, 2024. The copyright holder for this preprint (which was not certified by peer review) is the author/funder. This article is a US Government work. It is not subject to copyright under 17 USC 105 and is also made available for use under a CC0 license.

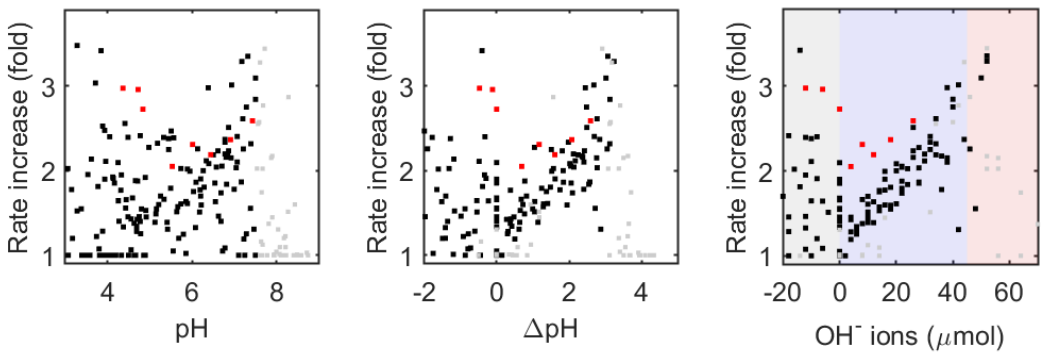

Figure S10: **NaOH input has a more consistent linear relationship with rate increase than pH or delta pH** To provide additional evidence that the NO_3^- reduction rate increase (fold) has a linear relationship with the added base, we calculated the rate increase (y-axis) independently from the model. To do so, we performed linear regression on the linear nitrate dynamics of chloramphenicol treated (CHL+) and untreated (CHL-) conditions, determining the slope ratio (CHL-/CHL+). On the leftmost plot, the rate increase is plotted against the perturbed pH (x-axis), against delta pH (= perturbed pH - native pH) in the central plot, and against the added amount of OH^- ions (in μ moles, negative values indicate the amount of H^+ ions) input on the rightmost plot. We are using perturbed samples from all soils with varying native pH levels. As we progress from left to right plots, we observe a greater collapse of data into a linear relationship with the rate increase. This confirms that NaOH is the most reliable descriptor for consistently explaining the growth due to nutrient release across soils with various native pH levels. Data points from Soil12 are colored in red owing to its slope being different from the collapsed slope of other soils (black points). Treatments with pH greater than 7.5 were colored gray, as they predominantly belong to the Resurgent growth regime (Regime III), while linearity is expected to only hold in the Nuitrient-limiting regime (Regime II). The blue background in the rightmost plot is a guide for the eye, indicating the range of perturbations that typically remain within the Nutrient-limiting regime (Regime II).

bioRxiv preprint doi: https://doi.org/10.1101/2024.03.15.584851; this version posted May 28, 2024. The copyright holder for this preprint (which was not certified by peer review) is the author/funder. This article is a US Government work. It is not subject to copyright under 17 USC 105 and is also made available for use under a CC0 license.

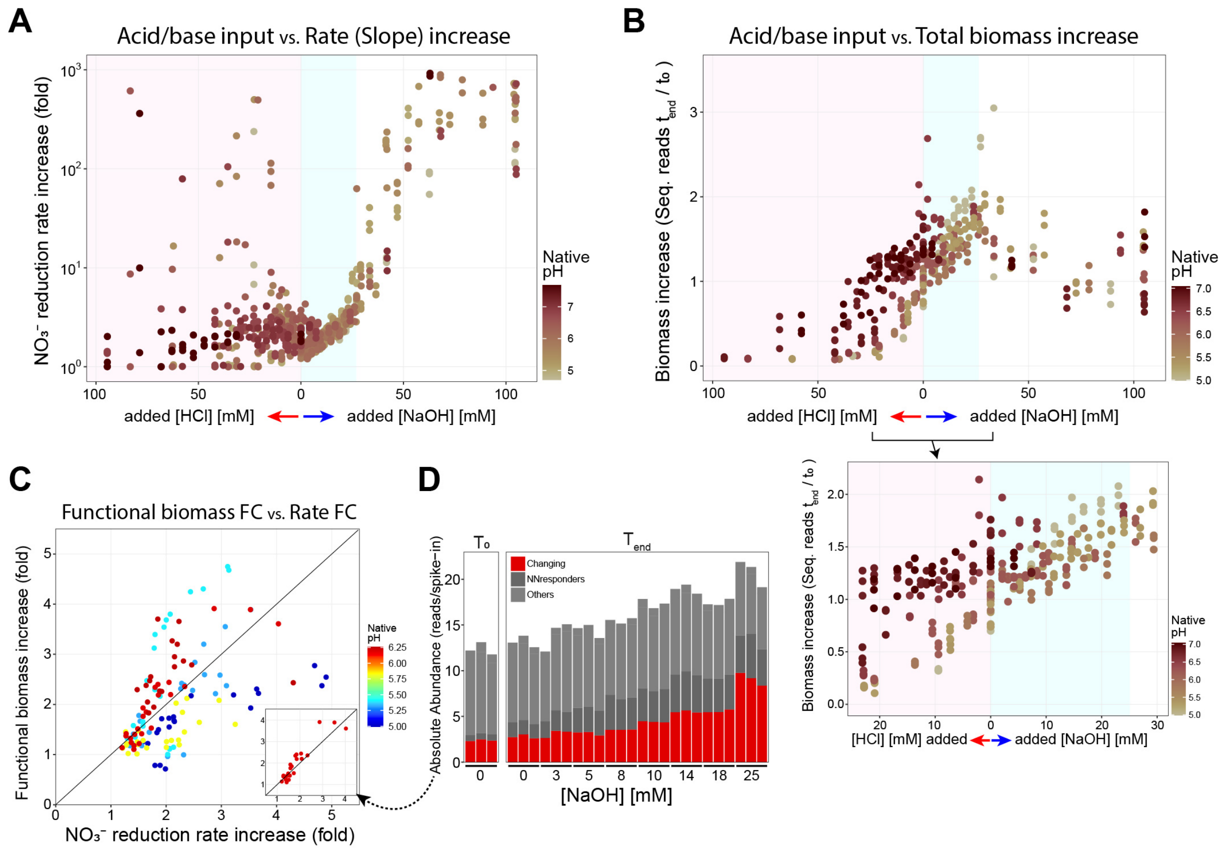

Figure S11: **Confirming the linear dependence between functional biomass and acid/base added** A more detailed analysis, accounting for individual Amplicon sequence variant) ASVs **(C, D)**that responded to the amendment of nitrate, further confirmed the linear dependence between biomass and acid/base added. **(A)** Showing the full range of acid/base input (x-axis) against the NO_3^- reduction rate increase (fold) (y-axis) in chloramphenicol-untreated (CHL-) conditions compared to treated conditions (CHL+) for all soils from different native pH (color gradient of data points). The rate fold increase is computed from the fitted model parameters $(1 + \gamma\tilde{C}_0/\tilde{x_0})$. This linear relationship is observed within the range of NaOH addition from 0mM to 25mM, which belongs to the Nutrient-limiting regime (Regime II) (light blue background in **A**). This was not the case for acidic perturbations (> 0mM HCl addition) and basic perturbations beyond 25mM NaOH addition. Therefore, the fitted model parameter suggests that the addition of NaOH causes the release of limiting nutrients in the soil, increasing biomass growth. **(B)** Showing the full range of acid/base input against biomass growth measured by the sequencing data. Biomass increase (fold) was computed with the ratio of the total absolute abundance of initial and end time points samples (T_{end}/T_0). We plotted an inset to highlight a zoomed-in range (< 25mM HCl, < 25mM NaOH). In this range, the amount of biomass growth evidently increases with the addition of NaOH (light blue background) and decreases with the addition of HCl (pink background) for all soils from different native pH levels (color gradient of data points). Although this linear relationship corroborates our proposed nutrient release mechanism, to be more precise, we need to prove further that the factor increase of the "functional" biomass equals the factor increase of nitrate reduction linear rate from the flux dynamics data. This is because not all biomass performs NO_3^- reduction. (Continued)

bioRxiv preprint doi: https://doi.org/10.1101/2024.03.15.584851; this version posted May 28, 2024. The copyright holder for this preprint (which was not certified by peer review) is the author/funder. This article is a US Government work. It is not subject to copyright under 17 USC 105 and is also made available for use under a CC0 license.

Figure S11: (Continued from the previous page) To detect the fractional biomass that performs denitrification, we used a differential abundance analysis to statistically determine which ASVs were significantly enriched in each pH perturbed condition compared to the CHL+ counterpart serving as a baseline of no growth (see Methods). We filtered out the ASVs that could be false-positive nitrate reducers by removing ASVs that were statistically enriched in no-nitrate conditions (dark grey NNresponders bar in **(D)**). Then, we summed up the absolute abundance of these ASVs that we inferred as true nitrate reducer biomass to obtain the functional biomass for each condition (red bar in **(D)**). **(C)** By comparing the factor increase of these functional biomass values (endpoint/initial functional biomass), we showed that indeed the functional biomass increase and denitrification rate increase are aligned in different soils (color spectrum in soils with different native pH). Some soils had these two values lie very close to the 1:1 diagonal line (Soil11, inset of **(C)**), which validates our inference procedure.

bioRxiv preprint doi: https://doi.org/10.1101/2024.03.15.584851; this version posted May 28, 2024. The copyright holder for this preprint (which was not certified by peer review) is the author/funder. This article is a US Government work. It is not subject to copyright under 17 USC 105 and is also made available for use under a CC0 license.

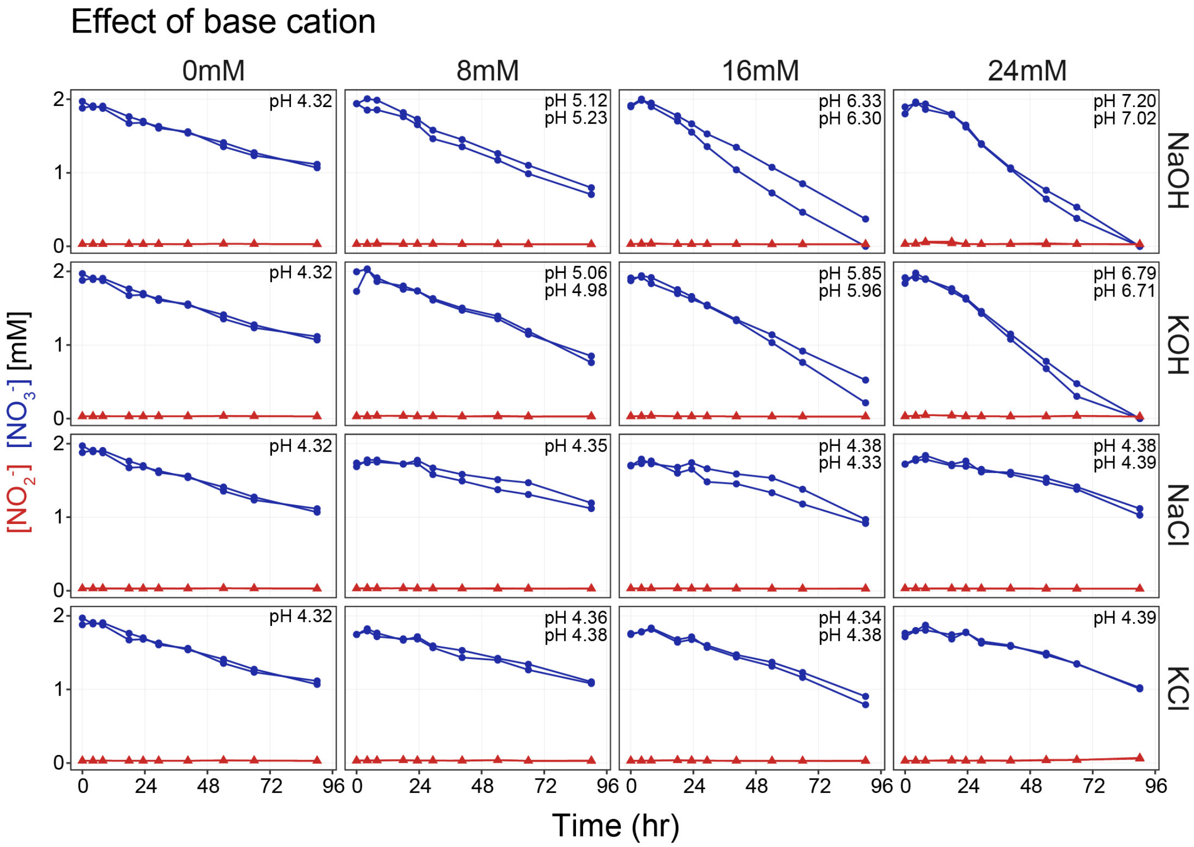

Figure S12: **Testing the effect of different bases and salts on nutrient release** To see the effects of different bases (NaOH and KOH) on nitrate reduction dynamics, we added different concentrations of NaOH and KOH (final concentration of 0, 8, 16, 24mM in the slurry), following the same protocol previously described (without chloramphenicol), to measure the nitrate and nitrite dynamics using Soil6 (Table S3). In addition, to test the effects of Na^+, K^+, and Cl^- separately, we added different concentrations of salts (NaCl, KCl) (without chloramphenicol and without adding any acid/base) and measured the metabolite dynamics. Blue points denote nitrate measurements and red points denote nitrite measurements. The lines connect the data points of two biological replicates. Each panel displays the stabilized endpoint pH (1M KCl method). Identical pH values across biological replicates are noted once.

bioRxiv preprint doi: https://doi.org/10.1101/2024.03.15.584851; this version posted May 28, 2024. The copyright holder for this preprint (which was not certified by peer review) is the author/funder. This article is a US Government work. It is not subject to copyright under 17 USC 105 and is also made available for use under a CC0 license.

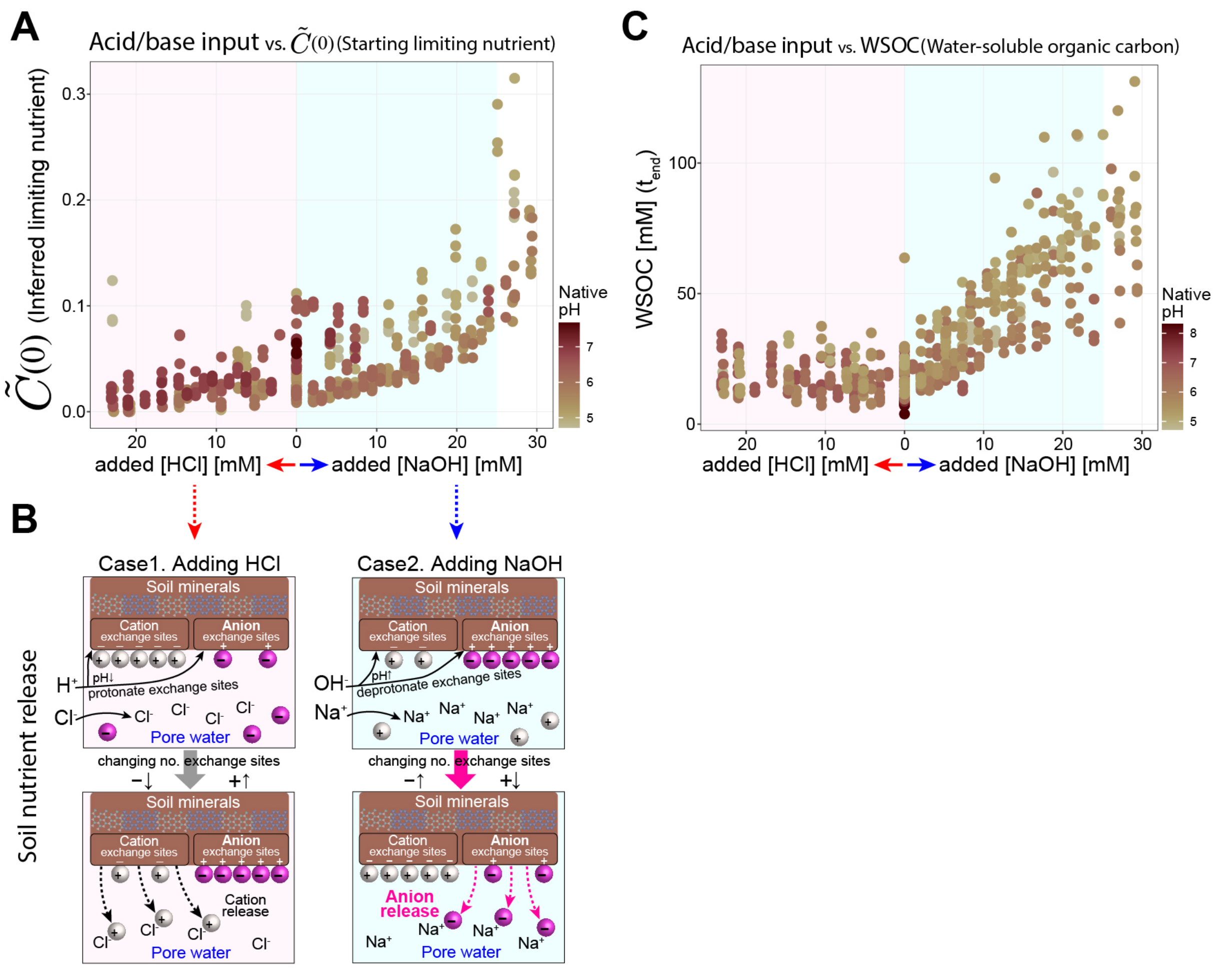

Figure S13: **Water-soluble organic carbon (WSOC) measurement aligns with the nutrient release hypothesis in the Nutrient-limiting regime (Regime II) (B)** Cartoon illustrating the soil nutrient release hypothesis; NaOH results in the release of anion nutrients from soil clay particles (brown region), while the addition of HCl releases cation nutrients and adsorbs anion nutrients. Microbes cannot access the nutrients adsorbed in the soil particles but can access the nutrients dissolved in soil pore water. Added OH^- ions deprotonate both cation and anion exchange sites, hence decreasing the number of anion exchange sites in the soil particles and increasing the number of cation exchange sites. This releases anion nutrients from the clay particles to the pore water, while cations in the pore water are adsorbed to the clay particles. In concert, added Na^+ ions stabilize the released anions in the pore water facilitating the release. On the other hand, during HCl addition, Added H^+ protonates both cation and anion exchange sites, hence increasing the number of anion exchange sites in the soil particles and decreasing the number of cation exchange sites. This releases cations from the clay particles to the pore water, while anion nutrients in the pore water are adsorbed to the clay particles no longer available to the microbes. In concert, added Cl^- ions stabilize the released cation in the pore water. (Continued)

bioRxiv preprint doi: https://doi.org/10.1101/2024.03.15.584851; this version posted May 28, 2024. The copyright holder for this preprint (which was not certified by peer review) is the author/funder. This article is a US Government work. It is not subject to copyright under 17 USC 105 and is also made available for use under a CC0 license.

Figure S13: (Continued from the previous page) **(A)** With this proposed mechanism of nutrient release by NaOH and HCl, we can further specify the type of growth-limiting nutrient by observing the change of the fitted model parameter of $\tilde{C}(0)$ (starting limiting nutrient). In natively acidic soils, increasing NaOH concentrations linearly increased the $\tilde{C}(0)$ (light blue region), which indicated that the limiting nutrient is negatively charged (anion nutrient). In natively neutral soils, increasing HCl concentration linearly decreased the $\tilde{C}(0)$ (light pink region). This is congruent with our statement that the growth-limiting nutrients are anions, because when HCl is added, anions are sequestered to the clay particles becoming unavailable to the microbes (purple spheres in **B**). **(C)** Coincidentally, adding NaOH linearly increased the water-soluble organic carbon (WSOC) concentrations present in the slurry at the endpoint, while adding HCl did not. This suggests two aspects related to our nutrient release hypothesis. Firstly, it appears that most water-soluble organic carbon (WSOC) may be negatively charged (anion). Secondly, the growth-limiting nutrient might be either the WSOC itself or another nutrient that is concomitantly released with organic carbon in the form of organic matter, including all carbon (C), nitrogen (N), sulfur (S), and phosphorus (P). If the limiting nutrient were WSOC, only a fraction of WSOC would be used as nutrient, because while $\tilde{C}(0)$ and WSOC are well correlated, the concentration of released WSOC is disproportionately higher (≈20-75 C mM) than the amount of limiting nutrient needed to deplete all 2mM NO_3^- in the system, as shown in Fig. 4D).

bioRxiv preprint doi: https://doi.org/10.1101/2024.03.15.584851; this version posted May 28, 2024. The copyright holder for this preprint (which was not certified by peer review) is the author/funder. This article is a US Government work. It is not subject to copyright under 17 USC 105 and is also made available for use under a CC0 license.

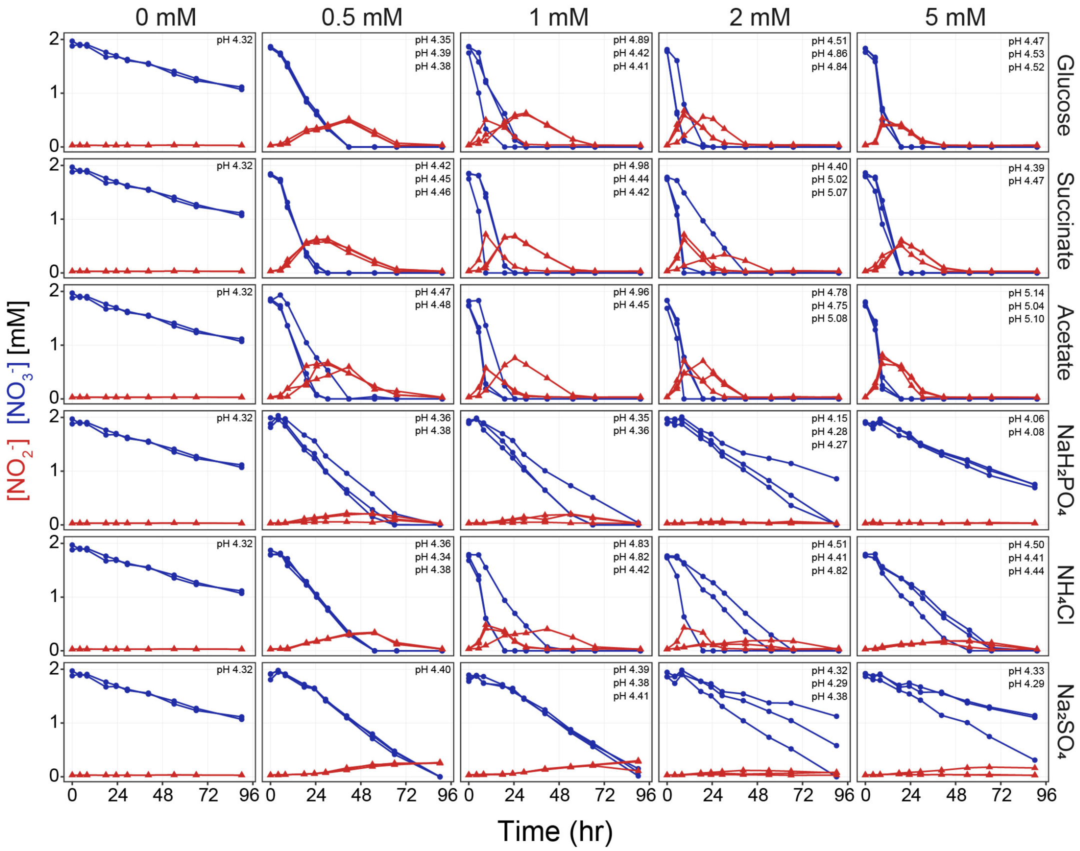

Figure S14: **Nitrate and nitrite dynamics of soils amended with different nutrients** To experimentally determine what nutrient was limiting growth in the Nutrient-limiting regime (Regime II), we conducted nutrient amendment experiments respectively with varying concentrations of glucose, succinate, sodium acetate, ammonium chloride (NH_4Cl), monosodium phosphate (NaH_2PO_4), and sodium sulfate (Na_2SO_4). Nitrate dynamics (blue) and nitrite dynamics were measured following the same protocol with 2mM NO_3^- (see Methods) using Soil6 (Table S3) without chloramphenicol and not adding any acid/base. Columns in the plot are different concentrations of C mM, N mM, S mM, or P mM in final concentrations in the slurry varying from 0 to 5 mM, each with biological replicates. 0mM amendment conditions are the same for all nutrients. Rows in the plots are different nutrients: C source (glucose, succinate, acetate), P source (phosphate), N source (ammonium), and S source (Sulfate). Each panel displays the stabilized endpoint pH (1M KCl method), and identical pH values across biological replicates were noted once. Among them, succinate (pK_a = 4.21 and 5.64, 25 °C), acetate (pK_a = 4.76, 25 °C), and phosphate (pK_a = 2.2, 7.2, and 12.4, 25 °C) were strong candidates for the limiting nutrient according to our soil nutrient release hypothesis, due to their anionic nature in mid-range pH (5-7). Because we have previously tested the effect of Na^+ and Cl^- to be negligible in nitrate dynamics, the effect of these amendments can be attributed solely to C/N/S/P nutrients other than Na^+ and Cl^-. We observed a transition from linear dynamics to exponential depletion of nitrate, when we amended the soil with a carbon source. Ammonium also made the nitrate consumption dynamics exponential in 1 mM amendment, but not in other amendment concentrations.

bioRxiv preprint doi: https://doi.org/10.1101/2024.03.15.584851; this version posted May 28, 2024. The copyright holder for this preprint (which was not certified by peer review) is the author/funder. This article is a US Government work. It is not subject to copyright under 17 USC 105 and is also made available for use under a CC0 license.

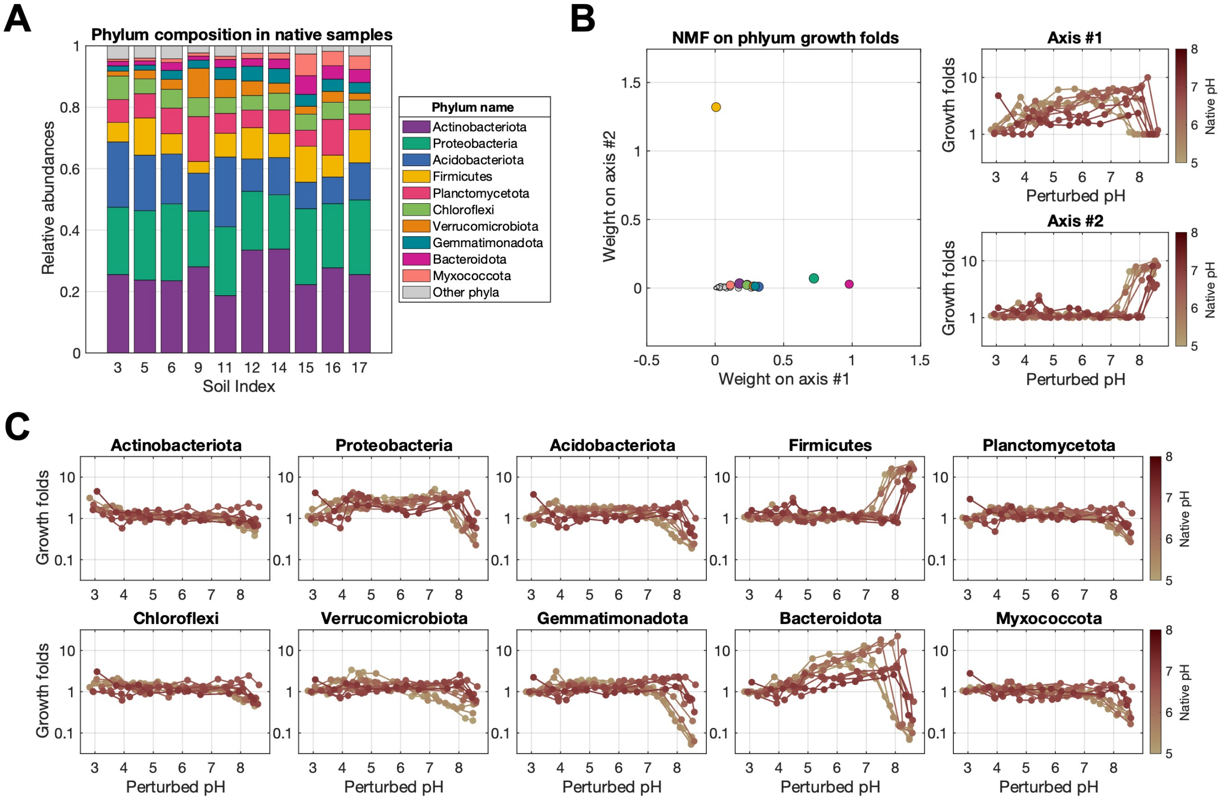

Figure S15: **NMF (Non-negative matrix factorization) reveals low-dimensional shifts in growth at the phylum level** **(A)** Initial community composition (T_0) of native soils in the phylum level. The x-axis indicates soils with different native pH levels (Soil 3, 5, 6, 9, 11, 12, 14, 15, 16, 17, see Table S3 for their properties). The y-axis represents the relative abundance (summed to 1) of the top 10 phyla out of 40, with the cumulative abundance of the remaining phyla depicted in gray as 'Other phyla'. **(C)** By using the absolute abundance of each taxon in chloramphenicol-treated (CHL+) conditions as a baseline value for growth in each perturbed pH condition, we computed the fold increase of each taxon's absolute abundance in chloramphenicol-untreated (CHL-) conditions, which we call growth fold (Abs_{CHL-}/Abs_{CHL+}). Ten different phyla showed idiosyncratic patterns of growth response along the varying perturbed pH. Soils with different native pH, indicated by the line color, showed relatively conserved growth trends in each phylum. **(B)** To systematically identify the underlying lower-dimensional growth response to pH, we used non-negative matrix factorization (NMF) on the growth fold values to decompose the growth response of all phyla into two modes (Axis #1 and Axis #2 in **B**, see Methods for details). Intriguingly, these two response patterns across pH matched the trend of functional parameters fitted with our consumer-resource model respectively for $\tilde{C}(0)$ and $\tilde{x}(0)$. The growth folds of each phylum are the linear combination of two modes whose weights are plotted on the left panel of **(B)** (points are colored by phylum as in **(A)**). Firmicutes phylum is mainly composed of mode #2, while other phyla are mainly composed of mode #1. Proteobacteria and Bacteroidota have higher weight #1 than other phyla. Therefore, this enabled us to focus our analysis on these phyla to explain the transition from the Nutrient-limiting regime (Regime II) to the Resurgent growth regime (Regime III).

bioRxiv preprint doi: https://doi.org/10.1101/2024.03.15.584851; this version posted May 28, 2024. The copyright holder for this preprint (which was not certified by peer review) is the author/funder. This article is a US Government work. It is not subject to copyright under 17 USC 105 and is also made available for use under a CC0 license.

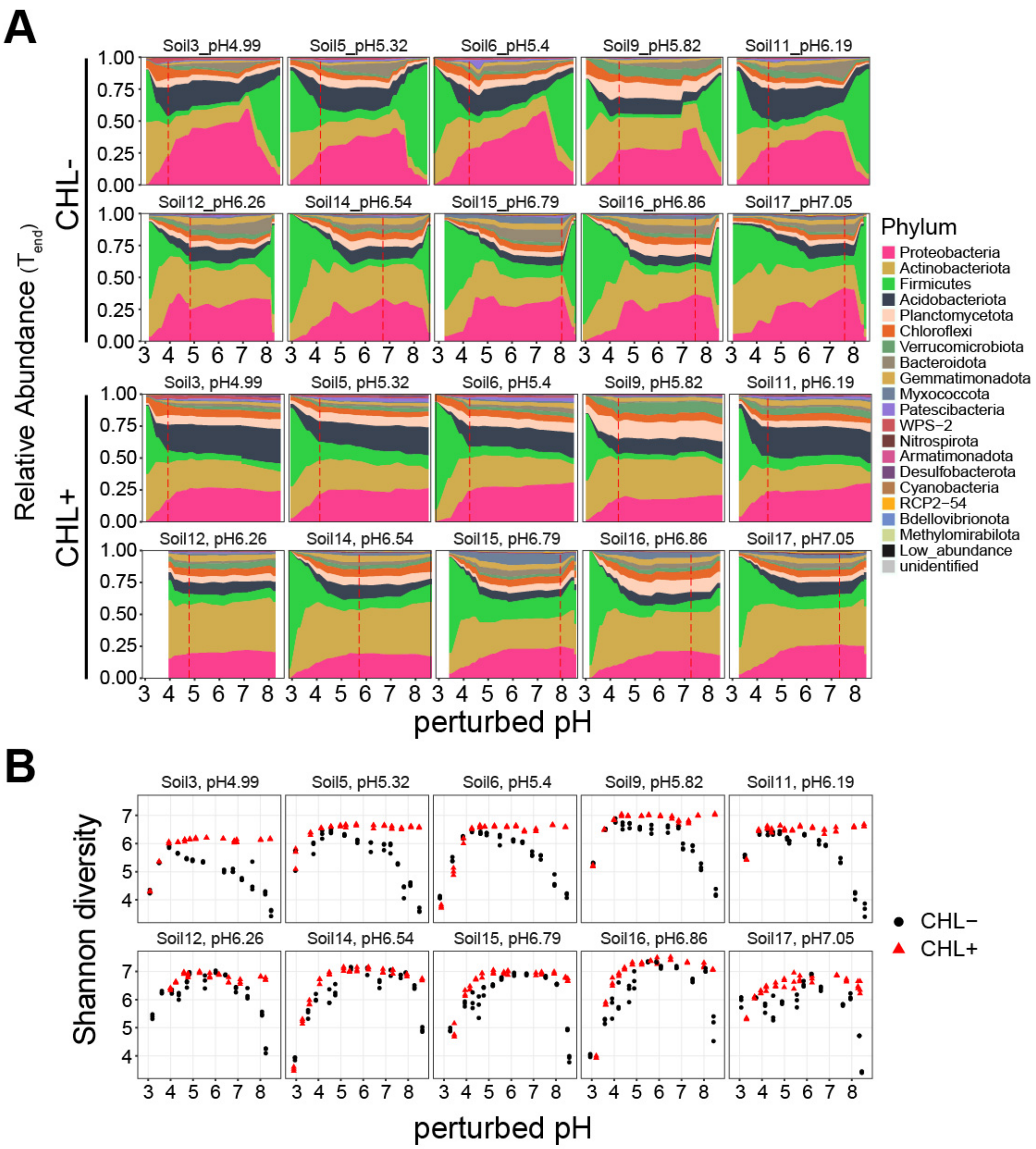

Figure S16: **Relative abundance and diversity of different soils across perturbed pH levels (A)** End-point relative abundance in the phylum level is plotted across the perturbed pH for ten different soils. CHL- indicates endpoint samples without chloramphenicol treatment. CHL+ indicates endpoint samples with chloramphenicol treatment. The alluvial plots were constructed by connecting the relative abundance values of 13 different pH perturbed levels. Red vertical dashed lines indicate the stabilized endpoint pH (1M KCl method) of the unperturbed samples. **(B)** Shannon diversity of the endpoint community is plotted across the perturbed pH for ten different soils in chloramphenicol-untreated(CHL-) and chloramphenicol-treated(CHL+) conditions.

bioRxiv preprint doi: https://doi.org/10.1101/2024.03.15.584851; this version posted May 28, 2024. The copyright holder for this preprint (which was not certified by peer review) is the author/funder. This article is a US Government work. It is not subject to copyright under 17 USC 105 and is also made available for use under a CC0 license.

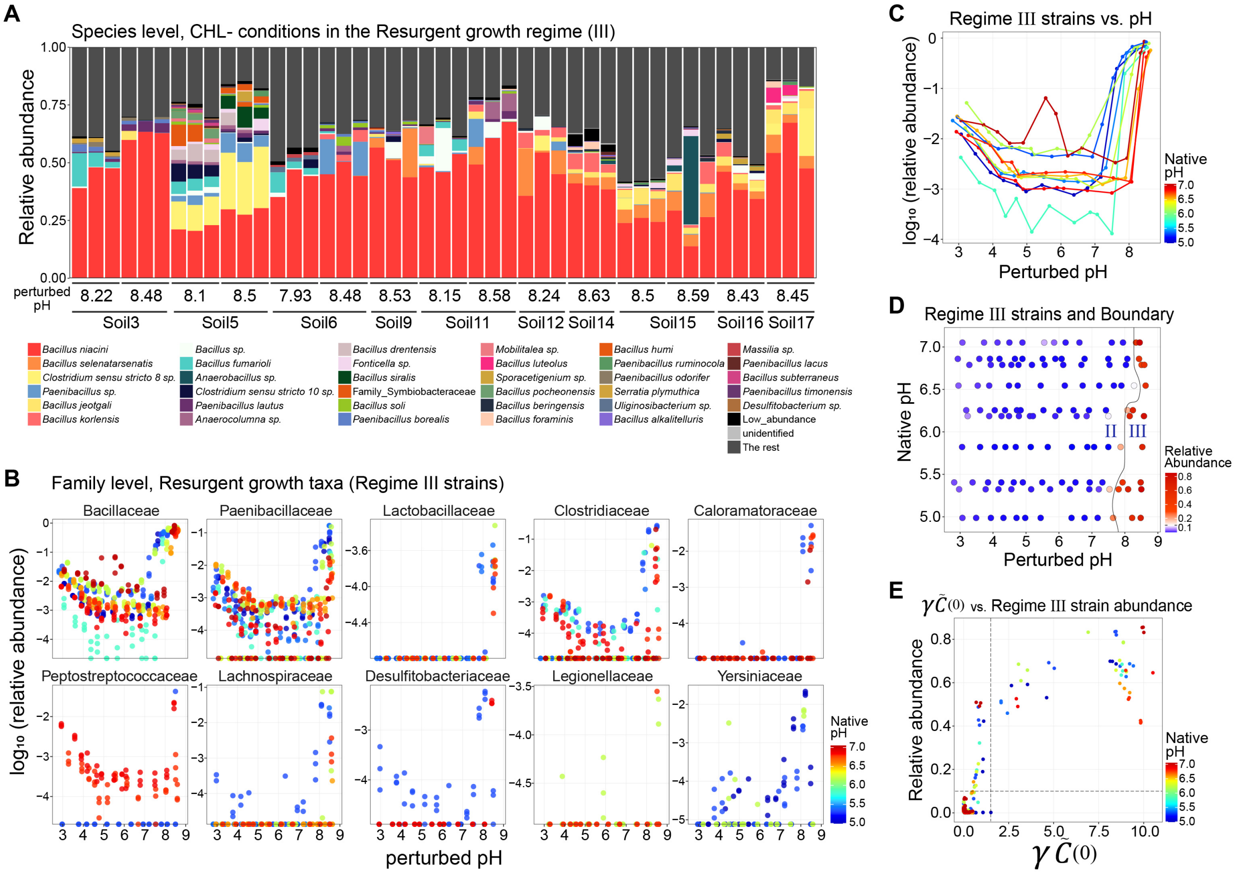

Figure S17: **Taxonomy of the identified Resurgent growth strains (Regime III strains) and their abundance agreeing with the functional Regime III** To identify the specific taxa accountable for the emergence of Regime III at a finer taxonomic level, we conducted a differential abundance analysis that statistically determined which Amplicon sequence variants (ASVs) were significantly more abundant in the Regime III CHL- samples, compared to CHL+ samples under same perturbed pH conditions (see Methods). **(A)** The relative abundance of the ASVs in all Regime III samples is highlighted and colored by their assigned species level. The ASVs not significantly enriched in Regime III samples are colored dark gray and labeled as ”The rest”. At the genus level, Bacillus, Clostridium, Paenibacillus, and others were identified as the primary contributors to the Resurgent growth regime (Regime III) as plotted. **(B)** The analysis revealed that 10 families belonging to Firmicutes (Bacillaceae, Paenibacillaceae, Clostridiaceae, Caloramatoraceae, Peptostreptococcaceae, etc.) and 2 families belonging to Proteobacteria phylum (Legionellaceae and Yersiniaceae) significantly enriched in the Resurgent growth regime (Regime III). Their relative abundance ($\log_{10}$ scale) increases at basic perturbed pH levels, patterns differing in soils with different native pH levels. Their relative abundance also slightly increases in Regime I, due to their high tolerance to pH perturbations. **(C-D)** Then, we aggregated the relative abundance of these differential ASVs (i.e., Regime III strains) to assess their contribution to the emergence of Regime III. Notably, their abundances ($\log_{10}$ scale) rise between pH 7-8, which aligns with or slightly precedes the transition between Regime II and III. **(E)** This increase in relative abundance corresponded with the rise of the nutrient growth parameter $\gamma\tilde{C}_0$ from zero.

bioRxiv preprint doi: https://doi.org/10.1101/2024.03.15.584851; this version posted May 28, 2024. The copyright holder for this preprint (which was not certified by peer review) is the author/funder. This article is a US Government work. It is not subject to copyright under 17 USC 105 and is also made available for use under a CC0 license.

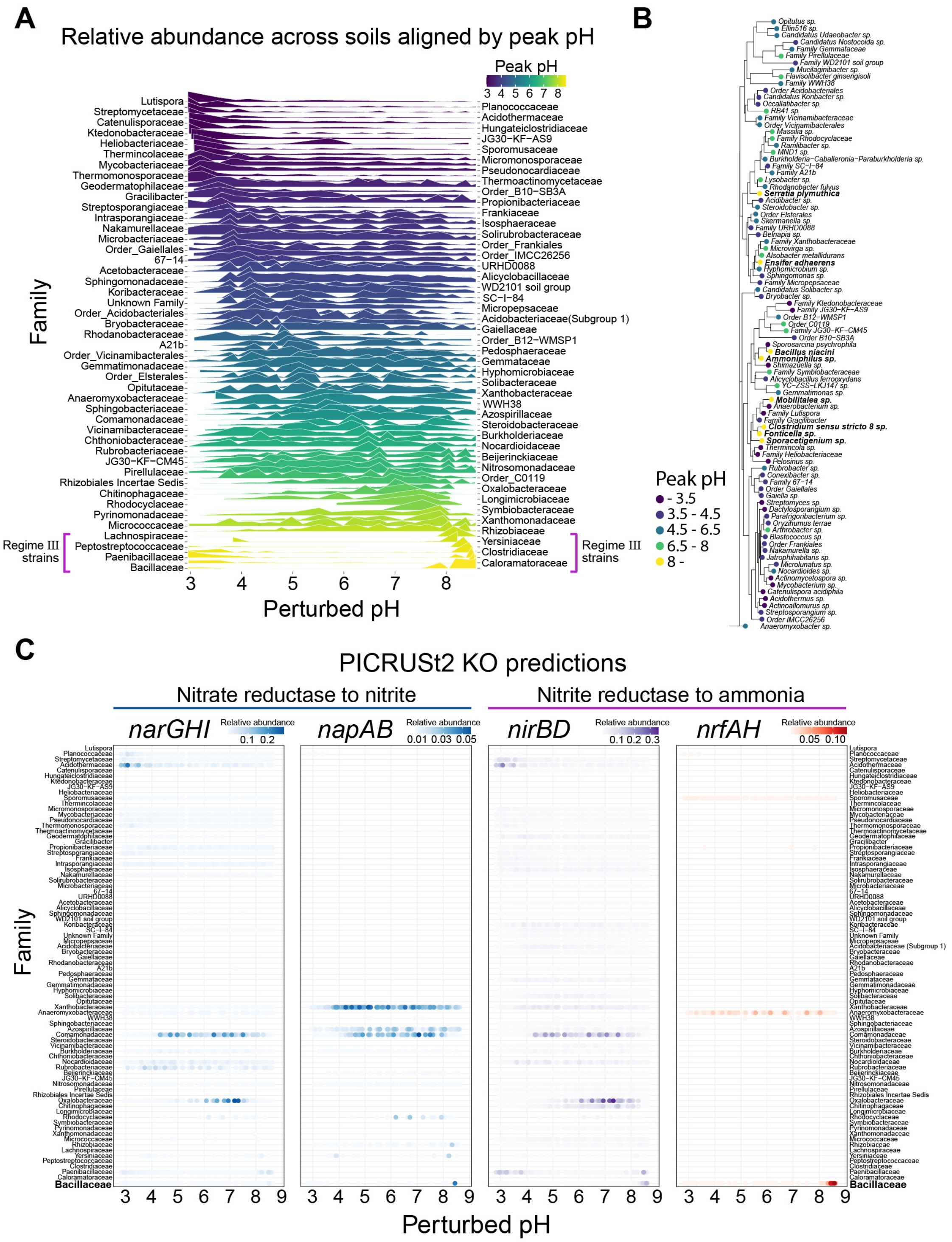

Figure S18:

bioRxiv preprint doi: https://doi.org/10.1101/2024.03.15.584851; this version posted May 28, 2024. The copyright holder for this preprint (which was not certified by peer review) is the author/funder. This article is a US Government work. It is not subject to copyright under 17 USC 105 and is also made available for use under a CC0 license.

Figure S18: **Traits of the Resurgent growth strains (Regime III strains) are analyzed through pH niche, phylogenetic distance, and gene predictions from PICRUSt2 (A)** To elucidate the pH niche of all taxa, we analyzed the relative abundance of amplicon sequence variants (ASVs) identified as being enriched in different pH levels (see Differential abundance analysis in Methods). We aggregated the relative abundance of ASVs in the same family for each sample, and then computed the median relative abundance of samples across different soils belonging to each pH bin (see Methods). The families were ordered by their peak pH (acidic peak pH on top with dark blue color and basic peak pH on the bottom with yellow color), which was the pH corresponding to the pH bin with the highest median relative abundance for each family. Indeed, the families belonging to the Resurgent growth strains had a peak pH over 8: Bacillaceae, Clostridiaceae, Paenibacillaceae, Caloramatoraceae, Peptostreptococcaceae, Lachnospiraceae (Firmicutes phylum), and Yersiniaceae (Proteobacteria phylum). **(B)** To see whether there was phylogenetic convergence among strains with similar pH niches, we used the 16S rRNA sequences of the ASVs to construct a phylogenetic tree. We selected one representative ASV with the largest relative abundance from each family to represent the family and used its 16S rRNA V34 region sequence to construct the phylogenetic tree. (see Method). Each node is labeled by the genus or species name and colored by its peak pH. The Resurgent growth strains (yellow-colored) did not cluster phylogenetically and were dispersed throughout the phylogenetic tree. **(C)** To infer genotypes of the Resurgent growth strains (Regime III strains), we used PICRUSt2 to predict the KEGG ortholog (KO) gene abundance from the 16S rRNA sequence of each ASV (see Methods). We focused on KOs/genes related to denitrification and Dissimilatory Nitrate Reduction to Ammonium (DNRA): nitrate reductase in denitrification (*narG*, *narH*, *narI*, *napA*, *napB*) and nitrite reductase to ammonium (*nirB*, *nirD*, *nrfA*, *nrfH*). To track which KOs were enriched at which pH in the 89 families used in the peak pH analysis in **A**, we summed the relative abundance (reads / total reads of each perturbed pH level in CHL-samples) of the ASVs belonging to each family that possessed at least 1 predicted gene respectively for *narGHI*, *napAB*, *nirBD*, and *nrfAH*. Then, we plotted their relative abundance values across pH for all soils, indicated by the intensity of the point's colors. For the Resurgent growth strain, Bacilli family exhibited a notable enrichment in *nrfAH* genes (indicated by red points), which are DNRA-related genes producing ammonium from nitrite.

bioRxiv preprint doi: https://doi.org/10.1101/2024.03.15.584851; this version posted May 28, 2024. The copyright holder for this preprint (which was not certified by peer review) is the author/funder. This article is a US Government work. It is not subject to copyright under 17 USC 105 and is also made available for use under a CC0 license.

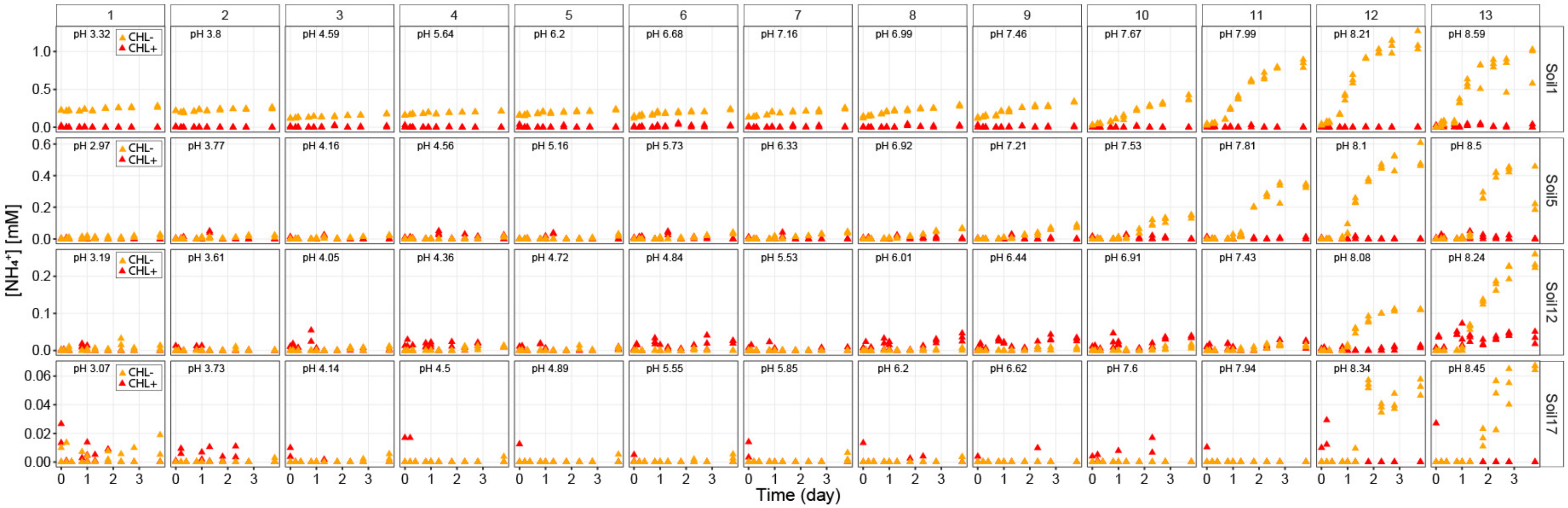

Figure S19: **Ammonium dynamics in chloramphenicol-untreated (CHL-) and treated (CHL+) samples in 4 soils with different native pH levels** For Soil 1, 5, 12, and 17 (for details of soils, refer to Table S3), the ammonium dynamics were measured colorimetrically using the Salicylate-hypochlorite assay [75]. Ammonium dynamics show that varying levels of NH_4^+ (from 3% (Soil17) to 50% (Soil1) of the provided 2mM NO_3^- converted to NH_4^+) accumulated in the Resurgent growth regime (Regime III). This indicates the activation of dissimilatory nitrate to ammonia (DNRA) pathway by a subset of strains responsible for the Resurgent growth regime. Chloramphenicol treatments in the samples (CHL+) led to consistent detection of 0.5mM NH_4^+ due to its N-H moiety. The NaOH concentration in perturbed samples also impacted NH_4^+ measurements, because the Salicylate-hypochlorite assay includes a step that OCl^- reacts with the N-H moiety resulting in N-Cl and OH^-, higher NaOH concentrations resulting in slightly lower detection of chloramphenicol in the CHL+ samples (0.45mM NH_4^+ in 100mM NaOH perturbations). Taking advantage of these control measurements, we used the constant NH_4^+ levels in the controls without 2mM NO_3^- (No-Nitrate controls) in the CHL+ conditions for each soil to offset the NaOH effect in the CHL- samples (by computing the conversion factor ratio of NH_4^+ levels of No-Nitrate controls in CHL+ conditions to the initial NH_4^+ levels of each condition with different NaOH additions in CHL+ samples) and to subtract NH_4^+ levels caused by chloramphenicol in CHL+ samples. pH indicated inside each panel is the endpoint stabilized pH (see Methods).

bioRxiv preprint doi: https://doi.org/10.1101/2024.03.15.584851; this version posted May 28, 2024. The copyright holder for this preprint (which was not certified by peer review) is the author/funder. This article is a US Government work. It is not subject to copyright under 17 USC 105 and is also made available for use under a CC0 license.

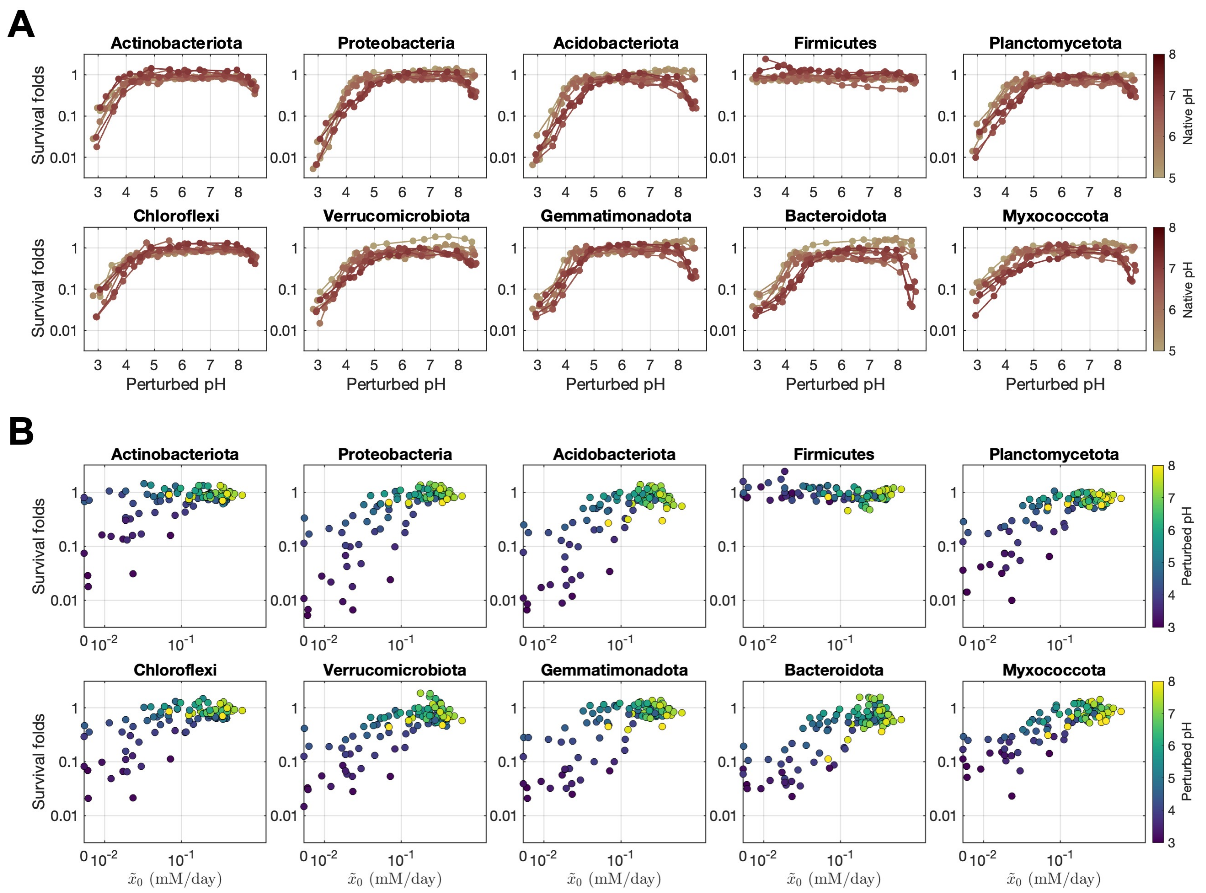

Figure S20: **Death of phyla during acidic pH perturbations explains Acidic death regime (Regime I)** **(A)** To infer death, the survival folds of the top 10 phyla (relative abundance-wise) among 40 taxa were determined for each perturbed pH condition by computing the fold difference in the endpoint absolute abundance of each Phylum under CHL+ conditions, relative to their baseline levels at the initial time point (Abs_{CHL+}/Abs_{T_0}). We used the abundance in CHL+ conditions to rule out growth and only compute the death effect of pH. A consistent drop of survival folds during acidic perturbation was observed across all phyla except for the Firmicutes phylum. **(B)** To check if the sequencing data supports our model parameter fitted from functional dynamics, we plotted the survival folds against the fitted $\tilde{x}(0)$ parameter (indigenous biomass activity) for each perturbed pH level denoted by the color gradient. We removed Regime III data points to focus on the Acidic death regime (Regime I). Indeed, during acidic perturbations (dark blue points), the survival folds decreased with the indigenous biomass activity $\tilde{x}(0)$ parameter fitted with functional data, except for only the Firmicutes. These results suggest that the impacts of acidic and basic pH perturbations on death are asymmetric rather than symmetric: while acidic conditions cause widespread death, death from basic conditions is less prominent.

bioRxiv preprint doi: https://doi.org/10.1101/2024.03.15.584851; this version posted May 28, 2024. The copyright holder for this preprint (which was not certified by peer review) is the author/funder. This article is a US Government work. It is not subject to copyright under 17 USC 105 and is also made available for use under a CC0 license.

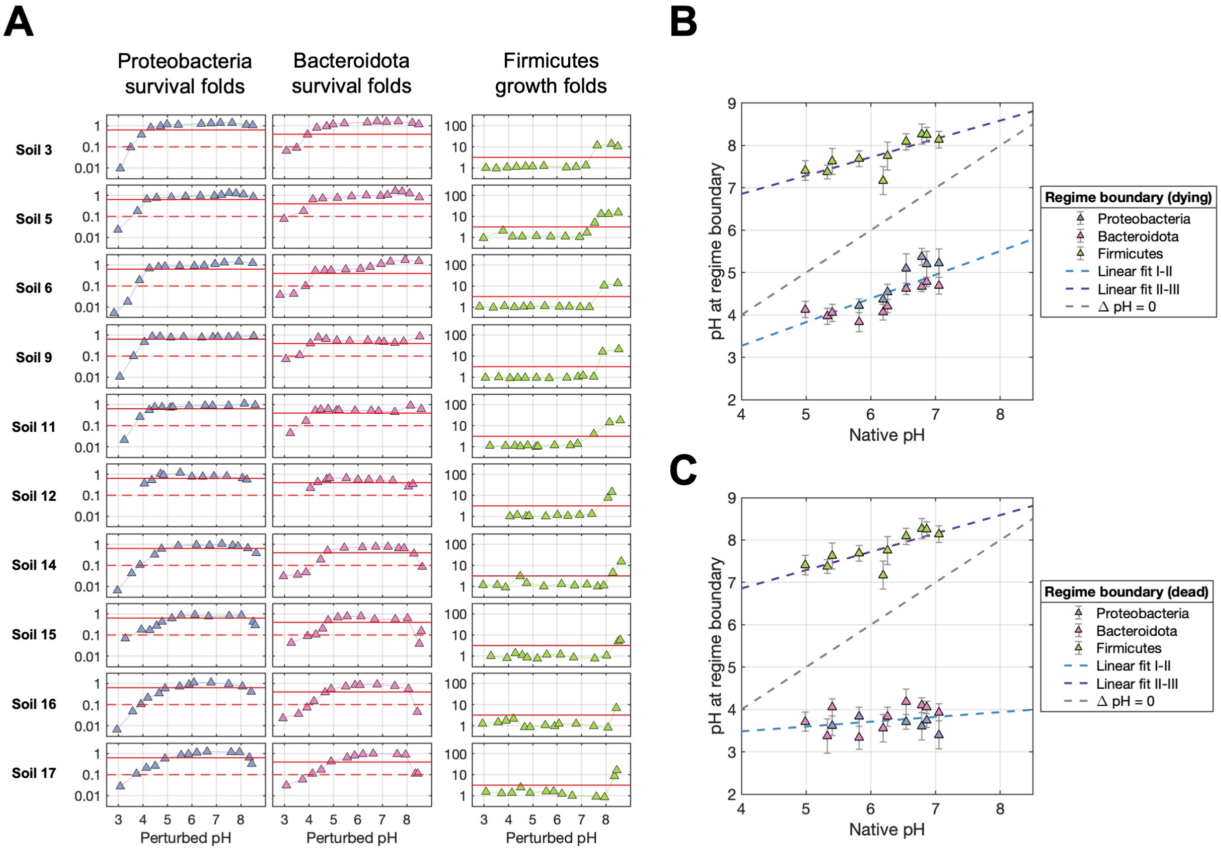

Figure S21: **Inferring regime boundary with phyla abundance dynamics** To understand the transition to the Acidic death regime (Regime I) with sequencing data, we computed the survival folds of Proteobacteria and Bacteroidota phyla across perturbed pH levels, which is the absolute abundance ratio of Abs_{CHL+}/Abs_{T_0} (endpoint absolute abundance in CHL+ conditions compared to the initial time point (T_0) for each perturbed pH level) (blue and pink data points in the left two panels). We set an identical survival fold threshold for all soils (red lines) to compute the pH at which the survival fold goes below that threshold during acidic perturbation. We used two distinct definitions to choose a threshold for the survival fold; (1) the "dying" definition where the taxa's abundance started to decline in abundance compared to T_0 (survival fold threshold < 1, red solid lines), and (2) the "dead" definition where the taxa's abundance was close to 0 (survival fold threshold $\rightarrow 0$, red dashed lines). To understand the transition to the Resurgent growth regime (Regime III) with sequencing data, we computed the growth folds of the Firmicutes phylum (green data point in the rightmost panel) by endpoint absolute abundance ratio of Abs_{CHL-}/Abs_{CHL+} (chloramphenicol untreated/treated conditions), and similarly computed the boundary pH at which the growth folds started increasing (threshold = 3, red solid lines). **(B)** These pH transition points were plotted against the native soil pH level. For the pH transition points in Proteobacteria and Bacteroidota, we used the "dying" definition. Employing the 'dying' definition with Proteobacteria, Bacteroidota allowed us to recapitulate the phenomenon observed in the functional data, where the fitted slope of Boundary I-II was less than 1, as shown in Fig. 6A. This suggests that these phyla in the relatively neutral soil are more tolerant of larger ΔpH change until they start to die than those in acidic soils, possibly due to variations in soil titration curves (Fig. 6B). (Continued)

bioRxiv preprint doi: https://doi.org/10.1101/2024.03.15.584851; this version posted May 28, 2024. The copyright holder for this preprint (which was not certified by peer review) is the author/funder. This article is a US Government work. It is not subject to copyright under 17 USC 105 and is also made available for use under a CC0 license.

Figure S21: (Continued from the previous page) Because the fitted slope is greater than 0, this also means that these phyla in relatively acidic soils can tolerate lower acidic pH conditions than those in neutral soils, which signals long-term pH adaptation. **(C)** The pH transition points were plotted against the native soil pH level using the ”dead” definition for Proteobacteria and Bacteroidota. The ”dead” definition threshold resulted in a flat slope close to 0. This suggests that, despite long-term adaptation to varying native soil pH levels, these taxa have similar pH thresholds at which complete decimation occurs. For **B and C**, error bars represent the pH difference between the two samples neighboring the survival or growth fold threshold. The points indicate the mid-point pH value of these boundary samples. The linear fit was determined using a least squares method (blue and purple dashed lines). The grey dashed line represents $y = x$, indicating hypothetical points where there is no difference between native and perturbed pH values ($\Delta pH = pH_{perturbed} - pH_{native} = 0$).

bioRxiv preprint doi: https://doi.org/10.1101/2024.03.15.584851; this version posted May 28, 2024. The copyright holder for this preprint (which was not certified by peer review) is the author/funder. This article is a US Government work. It is not subject to copyright under 17 USC 105 and is also made available for use under a CC0 license.

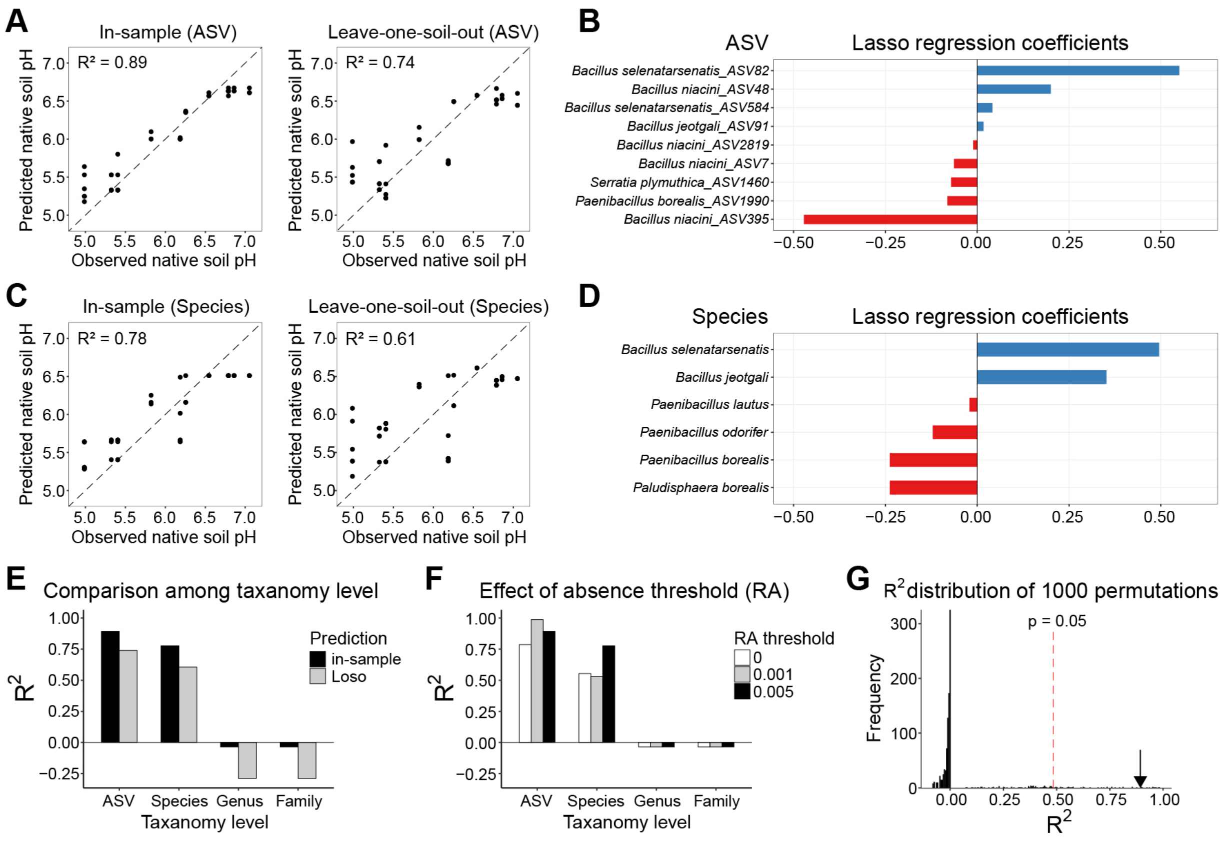

Figure S22: **Strain (ASV) and species-level variation of the Resurgent growth strains arise from the soil's natural pH environment** To investigate whether the taxonomic identity of Resurgent growth (Regime III) strains is determined by long-term adaptation to or selection from different natural pH environments, we performed a regularized regression analysis to see if we can predict the native pH level of the source soil from the presence or absence of Resurgent growth strains across different taxonomic levels (Amplicon sequence variant (ASV), Species, Genus, Family, or higher taxonomic levels) (see Methods). (Continued)

bioRxiv preprint doi: https://doi.org/10.1101/2024.03.15.584851; this version posted May 28, 2024. The copyright holder for this preprint (which was not certified by peer review) is the author/funder. This article is a US Government work. It is not subject to copyright under 17 USC 105 and is also made available for use under a CC0 license.

Figure S22: (Continued from the previous page) **(A)** Predicted and observed native soil pH with the Lasso regression from the presence and absence of the ASVs of Resurgent growth strains, using 0.005 (out of the relative abundance of 1) as a threshold for presence (see Methods). Left is the in-sample predictions, and the right is 'Leave-one-soil-out' (Loso) predictions where we leave out samples from one soil when we fit the regression model and then use the left-out samples to make predictions of their native pH as shown in the scatter plot. The prediction quality (R^2) was computed using the mean predicted and mean observed native pH levels for each soil. **(B)** Bar plots indicate the regression coefficients of all ASVs with non-zero coefficients from in-sample predictions in **A**. **(C)** Predicted and observed native soil pH from the Lasso regression from the presence and absence of the Resurgent growth strains in the species level. **(D)** Bar plots indicate the regression coefficients of all species with non-zero coefficients from in-sample predictions in **C**. **(E)** In-sample and Loso predictions are good only until the ASV and species level. From the genus level or higher, the predictions are worse than random (negative R^2 values). A relative abundance (RA) threshold of 0.005 was used for the presence and absence. **(F)** Effect of RA threshold values for the presence and absence (0, 0.001, and 0.005 out of 1). In the case of in-sample predictions, imposing the RA threshold improved the predictions at the ASV and species level. **(G)** To ascertain our prediction quality is not an artifact, we randomly permuted the native pH values 1000 times, and then predicted in-sample the native pH, showing that the R^2 value of 0.89 from in-sample predictions from **A** is greater than the top 50th R^2 value (0.475) out of 1000 shuffled predictions ($p = 0.05$).

bioRxiv preprint doi: https://doi.org/10.1101/2024.03.15.584851; this version posted May 28, 2024. The copyright holder for this preprint (which was not certified by peer review) is the author/funder. This article is a US Government work. It is not subject to copyright under 17 USC 105 and is also made available for use under a CC0 license.

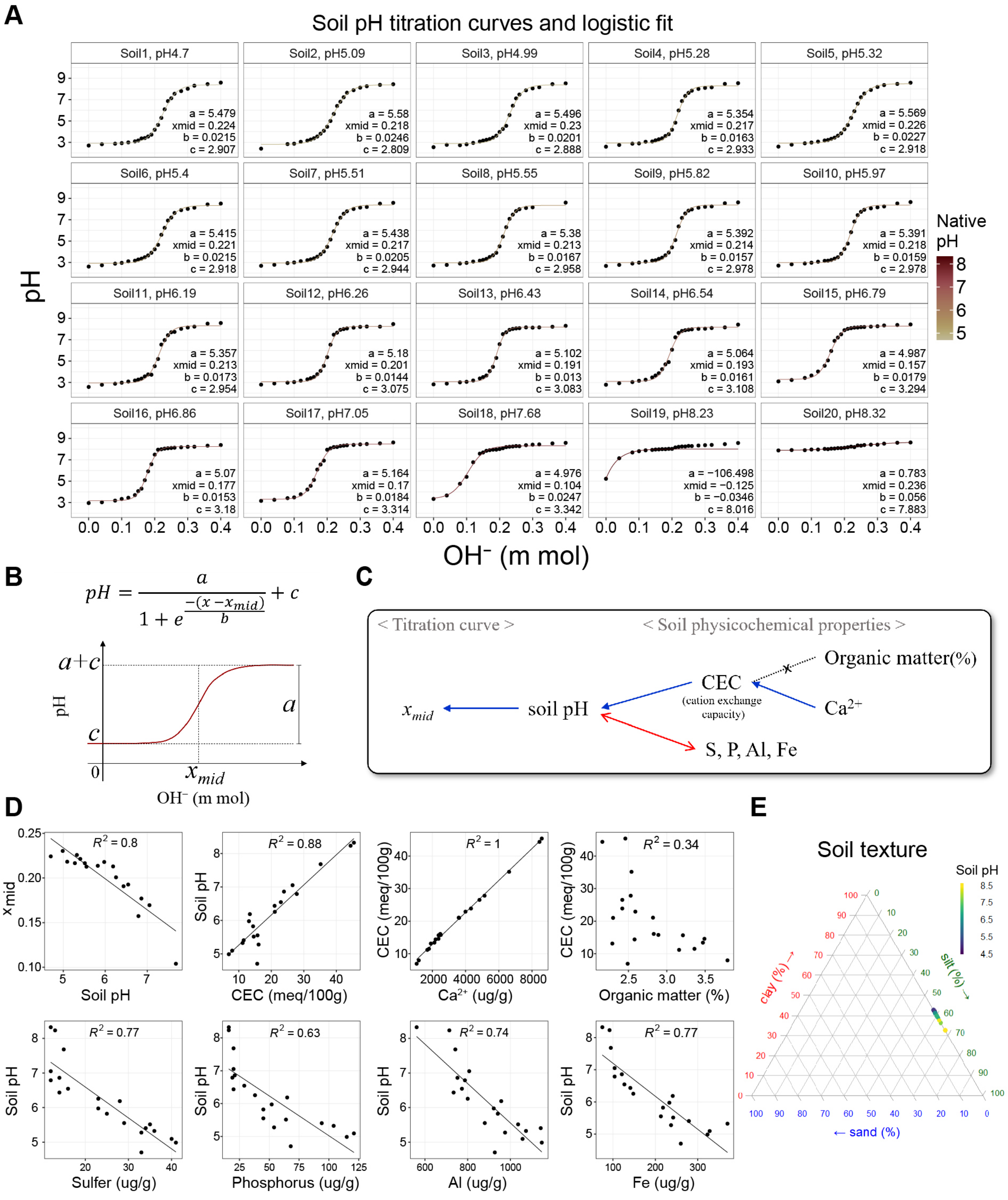

Figure S23:

bioRxiv preprint doi: https://doi.org/10.1101/2024.03.15.584851; this version posted May 28, 2024. The copyright holder for this preprint (which was not certified by peer review) is the author/funder. This article is a US Government work. It is not subject to copyright under 17 USC 105 and is also made available for use under a CC0 license.

Figure S23: **Relationship between pH titration curves and soil physicochemical properties** **(A)** Fitting logistic function to pH titration curves of the 20 soils from different native pH levels (see Methods). **(B)** Logistic function and parameters. **(C)** Summary of how soil's physicochemical properties can influence the soil pH titration curves. We can attribute the horizontal shift of the pH titration curves to their varying native soil pH levels, which are potentially determined by the Cation exchange capacity (CEC) and the Ca^{2+} ion concentrations. **(D)** The correlations that support the claim from the summary diagram **C** are shown with R^2 values. **(E)** Soil texture (sand, silt, clay percentage composition) was mostly identical for different soils, thereby not explaining the difference in soil pH levels and the titration curves.

bioRxiv preprint doi: https://doi.org/10.1101/2024.03.15.584851; this version posted May 28, 2024. The copyright holder for this preprint (which was not certified by peer review) is the author/funder. This article is a US Government work. It is not subject to copyright under 17 USC 105 and is also made available for use under a CC0 license.

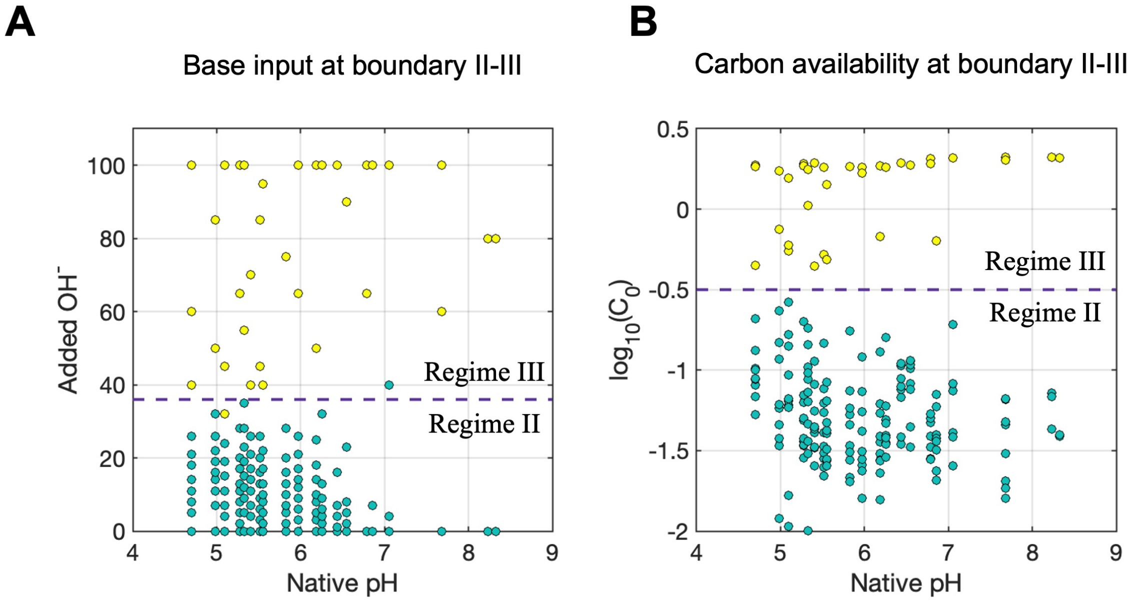

Figure S24: **Functional regime boundary II-III is dictated by the amount of NaOH and the available carbon nutrients. (A)** The relationship of the native soil pH and the amount of NaOH input (y-axis in mM) in Regime II samples (green data points) and Regime III (yellow data points). The dashed purple horizontal line indicates the NaOH input required to transition from Regime II to Regime III. The rather flat slope of pH boundary II-III vs. native pH in Fig. 6A can be explained by the fixed amount of NaOH input (dashed purple line in Fig. 6B). From our previous results, the amount of available carbon corresponds to the NaOH input. **(B)** Therefore, we plotted the relationship of the native soil pH and the fitted $\tilde{C}(0)$ values (log scale). We indeed observe the consistent amount of available carbon required to transition from Regime II to Regime III (dashed purple horizontal line).

bioRxiv preprint doi: https://doi.org/10.1101/2024.03.15.584851; this version posted May 28, 2024. The copyright holder for this preprint (which was not certified by peer review) is the author/funder. This article is a US Government work. It is not subject to copyright under 17 USC 105 and is also made available for use under a CC0 license.

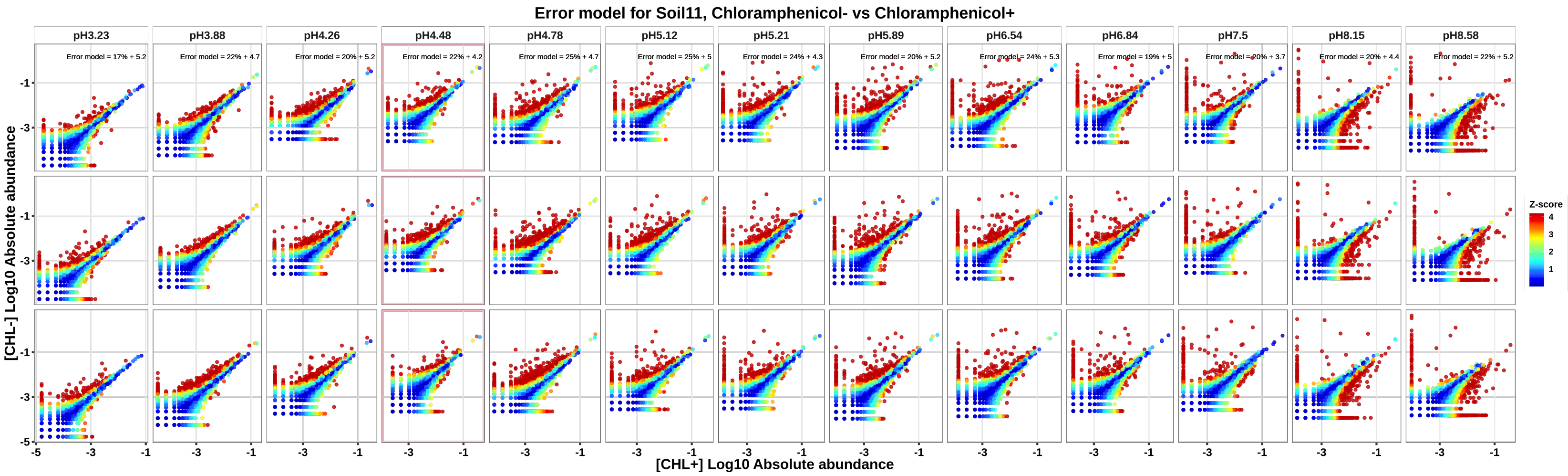

Figure S25: **Error model z score** To identify the ASVs enriched for each perturbed pH level, we empirically constructed a null model with the three biological replicates (see Methods). For each soil (Soil11 shown in the plot) and for each perturbed pH level (pH indicated in the header of each panel), we plot the log-scale absolute abundance of each ASV in chloramphenicol-treated samples (CHL+, x-axis) against the absolute abundance in chloramphenicol-untreated samples (CHL-, y-axis). Three rows of the panel indicate three biological replicate pairs. The deviations of replicate-replicate comparisons from 1:1 line are well-described by an effective model combining two independent contributions, a Gaussian noise of fractional magnitude c_{frac} and a constant Gaussian noise of magnitude c_0 reads, such that repeated measurements (over biological replicates) of an ASV with mean abundance n counts are approximately Gaussian-distributed with a standard deviation of $\sigma(c_0, c_{\mathrm{frac}}) = \sqrt{(c_{\mathrm{frac}} n)^2 + c_0^2}$ counts. In this expression, c_{frac} was estimated from moderate-abundance ASVs (> 50 counts) for which the other noise term is negligible; and c_0 was then determined as the value for which 67% of replicate-replicate comparisons are within $\pm\sigma(c_0, c_{\mathrm{frac}})$ of each other, as expected for 1-sigma deviations. This noise model was inferred separately for each soil and each perturbed pH level, as the corresponding samples were processed independently in different sequencing runs. The noise parameters are denoted inside the panel. The points are colored by the z-score computed from the Gaussian-distributed error model. The pink peripheral box indicates the condition without any acid/base addition.
